# Protective Effects of PGC-1α Activators on Ischemic Stroke in a Rat Model of Photochemically Induced Thrombosis

**DOI:** 10.3390/brainsci11030325

**Published:** 2021-03-04

**Authors:** Fatima M. Shakova, Yuliya I. Kirova, Denis N. Silachev, Galina A. Romanova, Sergey G. Morozov

**Affiliations:** 1 Institute of General Pathology and Pathophysiology, Baltiyskaya Str. 8, 125315 Moscow, Russia; bioenerg@mail.ru (Y.I.K.); romanovaga@mail.ru (G.A.R.); niiopp@mail.ru (S.G.M.); 2 A.N. Belozersky Research Institute of Physico-Chemical Biology, Moscow State University, Leninskye Gory 1, Bldg. 40, 119992 Moscow, Russia; silachevdn@genebee.msu.ru; 3 Histology, Embryology and Cytology Department, Peoples’ Friendship University of Russia, Miklukho-Maklaya Str. 6, 117198 Moscow, Russia

**Keywords:** focal ischemia, prefrontal cerebral cortex, PGC-1α, Mexidol, Semax, neuroprotection

## Abstract

The pharmacological induction and activation of peroxisome proliferator-activated receptor gamma coactivator 1 alpha (PGC-1α), a key regulator of ischemic brain tolerance, is a promising direction in neuroprotective therapy. Pharmacological agents with known abilities to modulate cerebral PGC-1α are scarce. This study focused on the potential PGC-1α-modulating activity of Mexidol (2-ethyl-6-methyl-3-hydroxypyridine succinate) and Semax (ACTH_(4–7)_ analog) in a rat model of photochemical-induced thrombosis (PT) in the prefrontal cortex. Mexidol (100 mg/kg) was administered intraperitoneally, and Semax (25 μg/kg) was administered intranasally, for 7 days each. The expression of PGC-1α and PGC-1α-dependent protein markers of mitochondriogenesis, angiogenesis, and synaptogenesis was measured in the penumbra via immunoblotting at Days 1, 3, 7, and 21 after PT. The nuclear content of PGC-1α was measured immunohistochemically. The suppression of PGC-1α expression was observed in the penumbra from 24 h to 21 days following PT and reflected decreases in both the number of neurons and PGC-1α expression in individual neurons. Administration of Mexidol or Semax was associated with preservation of the neuron number and neuronal expression of PGC-1α, stimulation of the nuclear translocation of PGC-1α, and increased contents of protein markers for PGC-1α activation. This study opens new prospects for the pharmacological modulation of PGC-1α in the ischemic brain.

## 1. Introduction

The high incidence of ischemic stroke and disability and the recurrence of acute impairments of cerebral circulation indicate the failure of therapeutic approaches used in the clinic and the need to develop an effective pathogenetically based targeted therapy [1]. One of the most promising approaches involves pharmacological stimulation of the expression/activity of the peroxisome proliferator-activated receptor-gamma coactivator (PGC-1α) [2,3,4,5,6].

**Figure 1:**
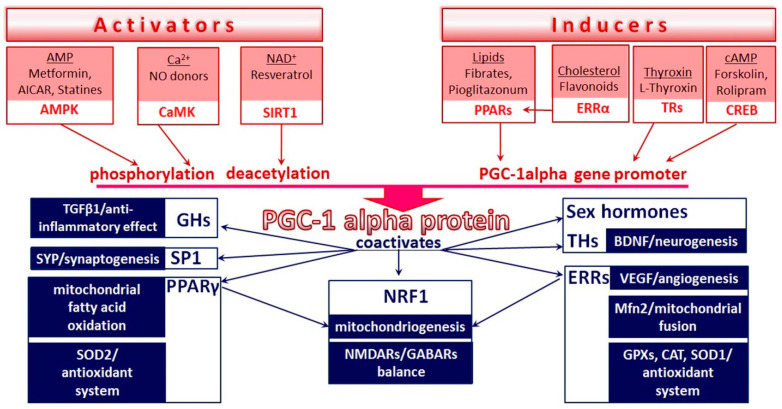
Mechanisms of positive regulation of PGC-1α by physiological/pharmacological modulators and basic PGC-1α-dependent molecular targets that determine neuroprotective effects. All arrows represent positive regulation (see explanations in text). Abbreviations: AICAR: 5-Aminoimidazole-4-carboxamide ribonucleotide; AMP: Adenosine monophosphate; AMPK: Adenosine monophosphate-activated protein kinase; BDNF: Brain-derived neurotrophic factor; CaMK: Ca^2+^/calmodulin-dependent protein kinase; cAMP: Cyclic adenosine monophosphate; CAT: Catalase; CREB: cAMP-responsive element-binding protein; ERRs: Estrogen-related receptors; GABARs: Gamma aminobutyric acid receptors; GHs: Glucocorticoid hormones; GPXs: Glutathione peroxidases; Mfn: Mitofusin; NAD: Nicotinamide adenine dinucleotide; NMDARs: N-methyl-D-aspartate receptors; NRF: Nuclear respiratory factor; PGC–1α: Peroxisome proliferator-activated receptor γ coactivator 1 α; PPARs: Peroxisome proliferator-activated receptors; SIRT: Silent information regulator two proteins (sirtuins); SOD: Superoxide dismutase; SP1: Specificity protein 1; SYP: Synaptophysin; TGF: Transforming growth factor; THs: Thyroid hormones; TRs: Thyroid hormone receptors; VEGF: Vascular endothelial growth factor.

PGC-1α was discovered in 1998 and has been widely studied as a key regulator of mitochondrial biogenesis and energy substrate delivery and utilization, as well as the main coordinator of the aerobic metabolism and energy homeostasis of cells [7,8,9]. The effects of PGC-1α are based on the coactivation of transcription factors controlling the expression of the proteins in the mitochondrial energy-producing system (NRF1/2; nuclear respiratory factor), fatty acid oxidation enzymes (PPARγ; peroxisome proliferator-activated receptor), transport proteins, mitochondrial dynamics factors, antioxidant enzymes, and transcription factors NRF1, PPARα, and ERRα (ERRα; estrogen-related receptor) [10,11,12,13,14]. NRF1/2, ERRα, and PPARγ form a transcription system for the regulation of tissue energy homeostasis orchestrated by PGC-1α (Figure 1).

The research over the last decade has revealed a novel aspect of the functional activity of PGC-1α, which involves the mediation of a wide range of neuroprotective effects, thereby maintaining neuron functionality and neuroplasticity. PGC–1α is expressed in all neuron populations in various cerebral regions, including the olfactory bulbs, the brain’s cortex, the hippocampus, the striatum, and the substantia nigra [15,16]. In the nervous system, PGC-1α-dependent mitochondrial biogenesis is coupled with dendritic sprouting, dendritic spine formation, and synaptogenesis [17,18]. By activating NRF1, PGC-1α regulates the expression of NMDAR, GABAR, and synaptic plasticity [19,20]. PGC–1α exerts antioxidant, antiapoptotic, anti-inflammatory, and trophic effects, mediates the reparative polarization of macrophages and microglia, and promotes angiogenesis and neurogenesis—the underlying mechanisms of neural cell survival that increase the brain’s tolerance to ischemia [21,22,23,24,25,26,27,28,29] (Figure 1). The crucial role of the CREB transcription factor in neuroreparative processes and the formation of neuron tolerance to ischemia, which is widely accepted in neurobiology, is based on this factor’s ability to bind to CRE sites in the promoter of the PGC-1α gene and activate its transcription [30]. PGC-1α is activated by the main sensors of the energy deficiency and redox state of the cell (AMPK, SIRT1), integrates metabolic and neurohormonal information, and modulates neuron survival pathways [31,32]. In the nervous system, synaptic activity and calcium influx through GluRs induce the activation of Ca^2+^-calmodulin (CaM)-dependent kinases, Ras/MAPK, and AMPK signaling cascades, which increase the expression, the stability, and the activity of PGC-1α [8,30,33,34] (Figure 1). The transcription coactivator PGC-1α mediates the neuroprotective effects of numerous endogenous signal molecules (hormones, neurotrophins, and metabolites) and is a promising target for pharmacological interventions in the treatment of various neurological and neurodegenerative diseases.

Despite the prospects for the pharmacological modulation of PGC-1α activity, there is a paucity of pharmacological agents capable of stimulating the activity and expression of PGC-1α in the brain. The clinically used drugs with known PGC-1α-modulating activity, such as fibrates, thiazolidinediones, metformin, and statins, display neurotoxicity and a limited ability to cross the blood–brain barrier (BBB) [35,36,37,38]. Therefore, efficient and safe PGC-1α inductors/activators are not actually present in neurological disease treatments. One of the possible approaches to extending the list of PGC-1α-modulating drugs may involve the search for PGC-1α inductors/activators among the clinically used preparations displaying multiple receptor-dependent neuroprotective effects that can potentially be mediated by the PGC-1α activity. The trend of searching for multitarget neuroprotectors that modulate PGC-1α is increasing in modern neuroscience [39]. The high therapeutic potential of stroke treatment is associated with neuropeptides (neurotrophins and neurohormones) exerting anti-apoptotic and anti-inflammatory effects and stimulating synaptogenesis and neurogenesis. PGC-1α activation has been shown to mediate the neuroprotective effects of BDNF [17]. Currently, studies are underway for the creation of neuropeptide mimetics that are resistant to the action of peptidases and can cross the BBB [40,41,42]. One of these drugs is the synthetic peptide Semax (a fragment of adrenocorticotrophin (ACTH)_4_–_7_ conjugated with the C-terminal tripeptide Pro-Gly-Pro), which has been used as a nootropic and neuroprotective drug [43,44]. It has been shown that Semax decreases ischemic brain damage in clinical and animal studies by inducing the expression of neurotrophins and their receptors [45,46,47,48,49,50]. Semax contains a fragment of an amino acid sequence critical for the ACTH function but without hormonal activity [51]. ACTH exerts neuroprotective and nootropic effects in the central nervous system through the activation of melanocortin receptors (MCRs), expressed by neurons, astrocytes, microgliocytes, and oligodendrocytes [52].

Another promising strategy for the stroke treatment consists of the use of metabolite-drugs aimed at maintaining energy production in neurons and increasing their tolerance to ischemia/hypoxia. Adaptogenic drugs include Mexidol (2-ethyl-6-methyl-3-hydroxypyridine succinate), used for the treatment of patients with cerebral ischemia [53,54]. In a clinical and experimental stroke, the Mexidol treatment was associated with a decrease in brain damage and the efficient recovery of cognitive and sensorimotor functions [55,56,57,58]. Mexidol can cross the BBB and exerts an antioxidant effect, as it contains a phenol antioxidant ethylmethylhydroxypyridine. Moreover, it has an energotropic mechanism, mediated by succinate, and a signaling mechanism (angiogenic and vasodilating effects), based on the activation of the succinate receptor SUCNR1/GPR91 [59,60,61].

This study aims to explore the PGC-1α-modulating activity of Semax and Mexidol belonging to different pharmacological groups on a rat model of focal ischemia in the prefrontal cortex.

## 2. Materials and Methods

### 2.1. Animals

We used 96 white outbred Wistar male rats weighing between 200 and 230 g and aged 8–9 weeks at the beginning of the experiment. The animals were obtained from the animal facility of the Institute of General Pathology and Pathophysiology, Russia. The animals were kept in a vivarium under natural light/dark cycles with free access to standard granular feed and water. The animal protocols used in this work were evaluated and approved by the institutional animal ethics committee (the project approval protocol no. 6 of 23 November 2018; the final approval protocol no. 3 of 16 June 2020) in accordance with the Russian National Standard R-53434-2009 “Principles of Good Laboratory Practice”, Directive 2010/63/EU of the European Parliament and the Council of the European Union for the Protection of Animals Used for Scientific Purposes.

### 2.2. Induction of Focal Ischemic Stroke

**Figure 2:**
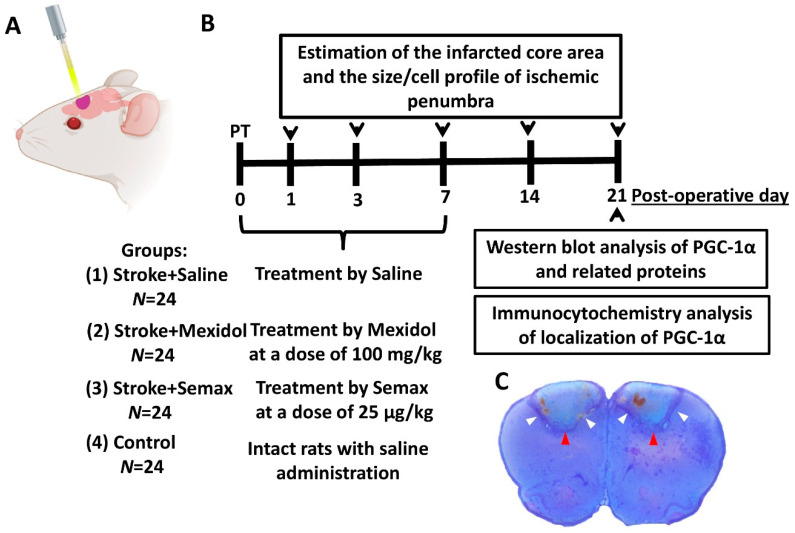
(**A**) The scheme of modeling of the photothrombotic stroke. Photothrombosis of blood vessels is achieved via intravenous injection of Rose Bengal (40 mg/kg) and illumination with a white light (250 W halogen lamp) through a light fiber (inner diameter is 3 mm) of the skull over the prefrontal cortex. (**B**) Experimental timeframe. (**C**) Brain section stained with cresyl violet illustrating the bilateral sites of photothrombotic stroke induction. White and red arrows represent the lateral and central sites of penumbra, correspondingly, selected for the size/cell profile of ischemic penumbra analysis. Created with BioRender.com.

Bilateral focal ischemic stroke was modeled in the rat prefrontal cortex (fields Fr1 and Fr2) by photochemically induced vascular thrombosis (PT) (Figure 2A) [62,63]. The rats were anesthetized with an intraperitoneal (i/p) injection of 300 mg/kg chloral hydrate. Additionally, to ensure proper pain relief in the perioperative and postoperative periods, we used repeated topical application of a long-acting local anesthetic, bupivacaine ointment. After the administration of the photosensitive dye rose Bengal (40 mg/kg intravenously; Sigma-Aldrich, St. Louis, MO, USA), the rat’s head was fixed in a stereotaxic frame, and the skull was exposed through a midline incision, cleared of the periosteum. The apparatus used for irradiation consisted of a source of cold light (250 W halogen lamp) and a light fiber with an inner diameter of 3 mm. The light fiber was placed at a distance of 1 mm from the cranial surface at 2 mm rostral to the bregma and 2 mm lateral to the sagittal suture. Each hemisphere of the brain was irradiated with cold light at λ = 560 nm for 15 min. The body temperatures of the rats during surgery were maintained at 37 ± 0.5 °C using a temperature-controlled heating blanket. After suturing the skin, the rats were placed in a cage under an infrared heating lamp until their recovery from anesthesia.

### 2.3. Study Design and Drug Treatment

The rats were randomly divided into the following four groups (Figure 2B): (1) “Stroke + Saline” (*n* = 24): Rats with photochemically induced thrombosis that received i/p saline; (2) “Stroke + Mexidol” (*n* = 24): Animals treated with Mexidol (LLC NPK Farmasoft, Russia) by i/p injection 2 h after PT and for 6 consecutive days (one injection per day) at a dose of 100 mg/kg; (3) “Stroke + Semax” (*n* = 24): Animals treated with Semax (CJSC Innovative Research and Production Center “Peptogen”, Russia) by i/n administration 2 h after PT and for 6 consecutive days (one instillation per day) at a dose of 25 μg/kg; (4) and “Control” (*n* = 24): Intact rats that received i/p saline. The animals in the Stroke + Saline and Control groups received saline according to the same regimen and at the same dosage as those in the Stroke + Mexidol group.

### 2.4. Estimation of the Infarcted Core Area and the Size/Cell Profile of Ischemic Penumbra

**Figure 3:**
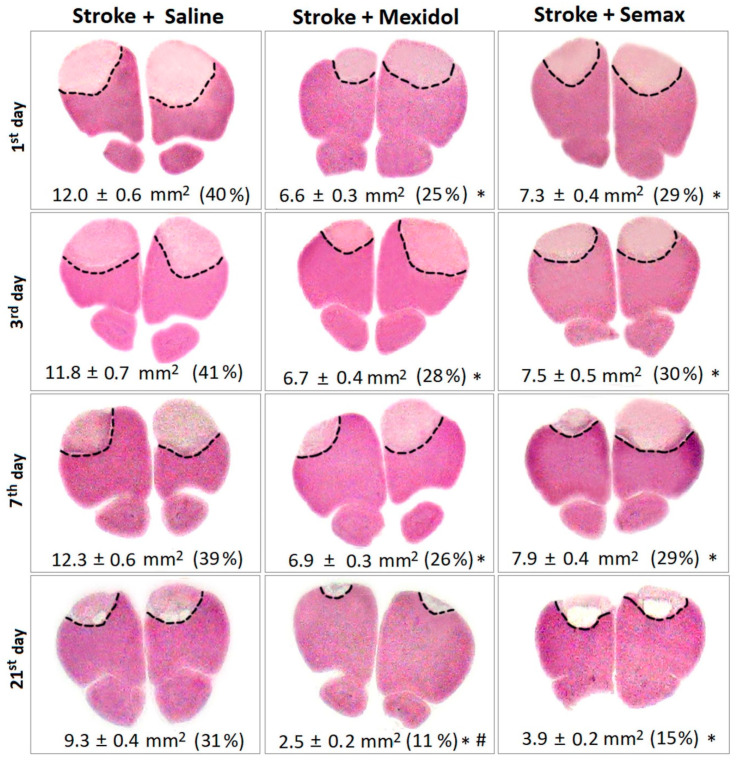
Dynamics of the development of ischemic damage of the prefrontal brain cortex in rats during the 7-day course of Mexidol or Semax treatment and 14 days after drug withdrawal. Photographs of rat prefrontal cortex slices were taken in the region with maximal cortex damage (at the level of 4.2 mm from the bregma) [62]. The slices were stained with hematoxylin and eosin (H&E). The border of the infarct area is marked with a dashed line. Under each image, the values of the sum of the damaged areas in both hemispheres are indicated, and the damaged area as a percentage of the prefrontal cortex is indicated in brackets. * *p* < 0.05 denotes a significant difference from the Stroke + Saline group at the appropriate time period; # *p* < 0.05 denotes a significant difference between the Stroke + Mexidol with Stroke + Semax groups at the appropriate time periods based on a two-way ANOVA with Bonferroni’s post-hoc multiple comparison test.

**Figure 4:**
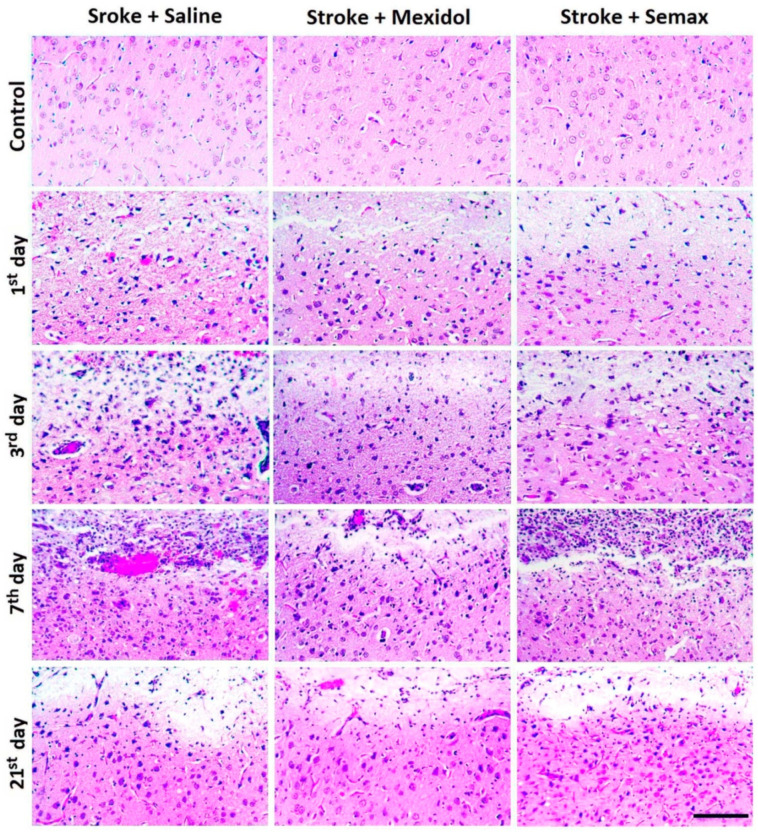
Representative microphotographs of pathomorphological alterations in the penumbral area within 21 post-ischemic days and a 7-day course of Mexidol and Semax treatments. Frontal section through the infarction and penumbra. A fragment of the infarction (pale-stained area) is shown at the top of the photomicrographs. The slices were stained with H&E. Scale bar: 100 μm.

For the animals in each group, the experiment was finished on Days 1, 3, 7, and 21 after stroke modeling by decapitation. The brain was immediately extracted, fixed in a 4% paraformaldehyde solution in phosphate-buffered saline (PBS) for 24 h, and subjected to standard histological processing through dehydration, delipidating, and paraffin embedding. To define the infarct area and the depth of penumbra spreading, we used frontal sections (Figures 3 and 4) at a level of 4.2 mm from the bregma [62] with maximal damage. The serial slices were 7 μm thick. They were mounted on glass, deparaffinized, and stained with hematoxylin and eosin (H&E). The brain slices were photographed with a Canon Power Short A550 camera (Canon, Japan) and analyzed using a fluorescent microscope Micromed 3 LUM (Ningbo Sheng Heng Optics & Electronics Co., Ltd.; Ningbo, China). We morphometrically defined the infarct area, the percentage of the affected prefrontal cortex, and the penumbral extension. The border of the penumbra was defined according to three criteria: (1) The presence of hyperchromatic and pyknotic neurons alongside unaltered neurons, (2) the presence of phagocyting cells (neutrophils and macrophages), the cytotoxicity of which may result in neuronal death progression, and (3) the development of post-ischemic gliosis (Figures S1–S3). The penumbral zone was distributed in an uneven manner around the ischemic core. We defined the most extended central site of penumbra that was adjoined to the maximal thickness of the infarction zone and the less extended lateral sites of penumbra that border the thinned infarcted area (Figure 2C). The planimetric calculations and morphometric measurements of the microphotographs were completed using the ImageJ software (W. Rasband, NIH, Bethesda, MD, USA, http://rsb.info.nih.gov/ij). The total score number of penumbral neurons and phagocytic/migrating leukocytes (neutrophils, monocytes/macrophages) in histologic H&E-stained sections was based on unique morphological features of the normal neurons (large cellular bodies and nuclei with single prominent nucleoli), the degenerating neurons (intensely stained eosinophilic cytoplasm, a small/shrunken darkly stained (pyknotic) nucleus), neutrophils (distinct multilobulated nucleus; pale, finely granular cytoplasm), and monocytes/macrophages (eccentric bi- or trilobed, reniform, or horseshoe-shaped nuclei have loose chromatin; abundant pale gray-blue cytoplasm contains vacuoles and occasional faintly eosinophilic granules) (Figures S1–S3) [64,65,66]. The data were acquired by processing six randomized microphotographs (the images corresponded to 0.1 mm^2^ of the penumbra).

### 2.5. Western Blot Analysis

The rats from the experimental and the control groups were decapitated. The brains were then quickly extracted and washed in ice-cold normal saline. The prefrontal cortex samples were separated (2 mm of the cortex near the ischemic core area and the corresponding regions in the control rats) and stored in liquid nitrogen until all the samples were collected. The frozen samples were ground in liquid nitrogen. The cells were next lysed in a cooled buffer (50 mmol HEPES pH 7.6, 150 mmol NaCl, 2 mmol EGTA, 1% triton-X-100, 10% glycerin, 1 mmol dithiothreitol, 1 mmol Na_3_VO_4_, 1 mmol AEBSF, 60 μg/mL aprotinin, 10 μg/mL leupeptin, and 1 μg/mL pepstatin A) for 30 min. The supernatant (the cytoplasmic extract) [67] containing the target proteins was collected after centrifugation (30 min, 14,000× *g*; 4 °C), mixed with the loading buffer (4× Laemmli Sample Buffer), incubated for 5 min at 95 °C, and stored at −80 °C. The protein concentration in the samples was defined spectrophotometrically by a Bradford assay. The proteins of the prepared samples were separated in 10% polyacrylamide gel and transferred into a nitrocellulose membrane via electroelution. Non-specific antibody binding was blocked by incubation in 5% defatted milk containing PBS and 0.1% Tween-20 for 1 h. The incubation was performed overnight at 4 °C in a solution of the primary monoclonal antibodies (Santa Cruz Biotechnology Inc., Santa Cruz, CA, USA, 1:500; mouse antibody against PGC-1α (peroxisome proliferator-activated receptor gamma coactivator 1 alpha; sc–518025), NRF1 (nuclear respiratory factor 1; sc–101102), TFAM (mitochondrial transcription factor A; sc–166965), NDUFV2 (NADH dehydrogenase [ubiquinone] flavoprotein 2; sc–515589), SDHA (a flavochrome subunit of succinate dehydrogenase; sc–166909), cyt c1 (cytochrome c1; sc–514435), COX2 (cytochrome c oxidase subunit II; sc–514489), ATP5A (ATP synthase alpha chain; sc–136178), VEGF (vascular endothelial growth factor; sc–365578), VE-cadherin (vascular endothelial cadherin; sc–52751), SYP (synaptophysin; sc–136271), β-actin (sc–376421)), and the first polyclonal antibody, rabbit anti-GPR91 (Abcam plc, Cambridge, UK, ab41505). After washing, the blots were incubated for 60 min in a solution of the secondary antibodies and conjugated with horseradish peroxidase (goat anti-mouse IgG-HRP (ab6789), goat anti-rabbit IgG-HRP (ab205718), and anti-mouse m-IgGk BP-HRP (sc-516102)) at 1:5000). The proteins were detected by the ECL reagent interaction (Pierce Biotechnology, Rockford, IL, USA) on a Kodak film followed by densitometry in Adobe Photoshop 7.0 (Adobe Systems, San Jose, CA, USA). The levels of the target proteins were estimated according to the density of the obtained bands. Each band’s intensity was normalized to the total protein loaded in the lane using the Ponceau S dye [68,69], since β-actin expression decreased in the penumbra (Figures S4 and S5). All images of blots are presented in Figures S4–S27. The results were obtained in relative densitometric units (RDUs) and then expressed in percentages relative to the Control group.

### 2.6. Immunohistochemistry

Immunohistochemistry was used to determine the PGC-1α levels and intracellular localization in paraffined slices of the prefrontal brain cortex. The slices were deparaffinized in xylol and rehydrated in a descending concentration of alcohols. The epitope retrieval was performed by boiling the slices in a heated bath in 0.01 M citrate buffer pH 6.0 for 30 min. The non-specific antibody binding was blocked by incubation with 4% bovine serum albumin in PBS containing 0.1% triton X-100 and 0.1% NaN_3_ for 1 h at 4 °C. After blocking, the sections were incubated overnight at 4 °C with a primary monoclonal mouse anti-PGC-1α (1:50; Santa Cruz Biotechnology, USA; sc–518025). After incubation, the slices were washed in PBS and incubated with the secondary antibodies conjugated with phycoerythrin at a 1:500 dilution (Santa Cruz Biotechnology, USA; m-IgGk BP-PE; sc–51614) for 2 h in the dark at 4 °C. Negative controls were obtained using the application of PBS rather than the primary antibody. After a wash step, the slices were dried and placed under a cover of glass in an anti-fade fluorescence mounting medium (Abcam, Cambridge, MA, USA; ab104135). The fluorescence signals were detected with a fluorescence microscope Micromed 3 LUM (Ningbo Sheng Heng Optics and Electronics Co., Ltd.; China). Identification of the neurons and neuronal nuclei was carried out using antibodies for a highly specific marker of neurons, nuclear protein NeuN (Neuronal nuclei) [70] (Figures S28–S30). The above-described immunohistochemistry staining procedure was applied. Anti-NeuN rabbit polyclonal antibodies (1:500; Abcam, Cambridge, MA, USA; ab104225) and secondary goat polyclonal antibodies for rabbit IgG H&L Alexa Fluor^®^ 488 (1:500; Abcam, Cambridge, MA, USA; ab150077) were used. The quantitative analysis of the immunohistochemical specimens was performed using the VideoTesT-Morphology 5.2 (LLC “VideoTesT”, Russia) software. In the quantitative assessment of PGC-1α levels in the nucleus and perinuclear cytoplasm, the difference of NeuN signal strength in the nucleus and cytoplasm plays a crucial role as described previously [71]. In merged images (PGC-1α/NeuN), the nuclear area was outlined, the NeuN image was then made transparent, the PGC-1α image was switched to a monochrome mode (gray), and the densitometry of the nuclear region was finally performed. For densitometry of the PGC-1α signal in the perinuclear cytoplasm, a similar algorithm was used, the difference being that the nuclear area was excluded in the colocalized images of neurons to obtain a ring-shaped figure of the perinuclear cytoplasm (Figure S31). We determined the total number of PGC-1α-immunoreactive neurons as well as the number of neurons with high/medium/low nuclear PGC-1α-immunoreactivity (IR).

### 2.7. Statistical Analysis

Data analysis was performed using the statistical package included with Microsoft Excel V16.11.1 (Microsoft Corporation, Redmond, WA, USA) and Statistica 7.0 (StatSoft, Inc., Palo Alto, CA, USA). Values are given as the mean ± standard error of the mean (SEM). Western blot (results of densitometry) were expressed as a percentage of the change from the mean of the Control group values, where the Control was defined at 100%. Variance inhomogeneity was assessed with Levene’s test. Statistical differences between the groups were analyzed using a two-way ANOVA with Bonferroni’s post-hoc multiple comparison test. Differences were considered significant at *p* ≤ 0.05.

## 3. Results

### 3.1. The Influence of Mexidol and Semax Treatment on the Brain Injury and the Size and the Cellular Composition of the Penumbra

Focal photochemically induced thrombosis (PT) of the blood vessels in the prefrontal cortex resulted in the formation of reproducible foci of ischemia in each hemisphere (Figure 3). Morphometric analysis of the brain slices revealed that, 1 day after PT, the ischemic core in the so-called “Stroke + Saline” group accounted for 40% of the prefrontal cortex area (Figure 3). Three and 7 days after PT, the size of the brain injury remained the same. On Day 21 after PT, we observed a retraction of the damage site by 25% and the formation of cysts resulting from massive phagocytosis of the damaged tissue. In the “Control” group, no morphological alterations were observed in the prefrontal cortex. A single administration of Mexidol or Semax after 2 h of PT significantly decreased the size of the necrotic area by 1.8-fold and 1.6-fold, respectively, compared to the Stroke + Saline group (*p* < 0.05; Figure 3). Over the following 6 days of the treatment course, the size of the necrotic area did not differ significantly in either group. In the “Stroke + Mexidol” and “Stroke + Semax” groups at 21 days, we observed a contraction of the necrotic zone by 60% and 50%, respectively, compared to the first postoperative day. Morphometric analysis of the damaged area at 21 days in the groups receiving courses of Mexidol or Semax treatment over 7 days showed a significant decrease in the damaged region by 3.5-fold and 2.4-fold, respectively, compared to the Stroke + Saline group (*p* < 0.05; Figure 3). In the Stroke + Mexidol group, the damaged area was 1.5-fold smaller than that in the Stroke + Semax group (*p* < 0.05; Figure 3) at 21 days.

We performed a morphological analysis of the cellular composition of the transitional zone (the penumbra), as well as a morphometric analysis of its extent. In the Stroke + Saline group, the penumbral zone was distributed in an uneven manner around the ischemic core, where the most extended central site (up to 1.5 mm) was adjoined to the maximal thickness of the infarction zone, and the less extended lateral sites of the penumbra (0.2–0.6 mm) bordered the thinned regions of the infarct zone (Figure 3 and Figure 4). The courses of drug treatment influenced the cellular profile and the size of the penumbra.

**Figure 5:**
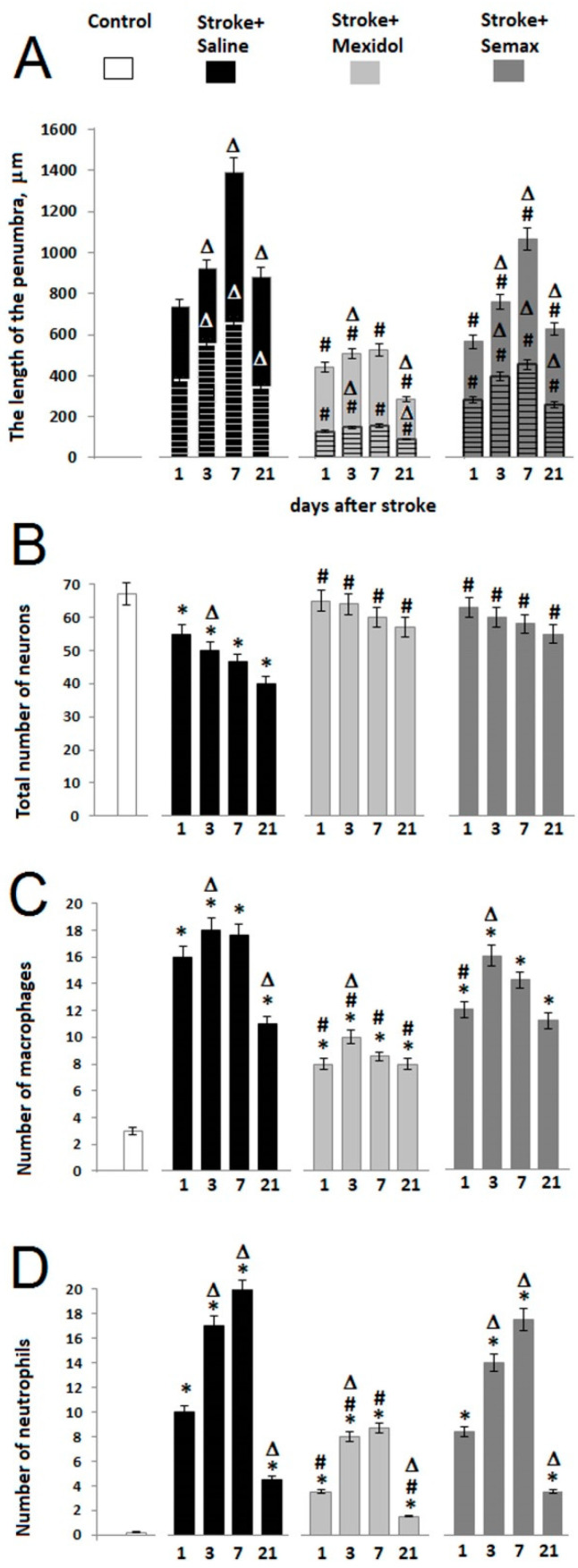
Effects of a 7-day Mexidol and Semax treatment course on the cellular composition of the penumbral zone at 1, 3, 7, and 21 days after PT. The diagram represents the quantitation of (**A**) the length of the penumbra, (**B**) neurons, (**C**) macrophages, and (**D**) neutrophils as the mean ± SEM. For Diagram A, the length (the distribution depth) of the central most extended site of the penumbra is represented by unhatched bars, while the lengths of the lateral sites are represented by the hatched bars. The experiments used six randomized microphotographs (images corresponding to the area of a penumbra of 0.1 mm^2^) of three slices from four rats. * *p* < 0.05 denotes a significant difference from the Control group; # *p* < 0.05 denotes a significant difference from the Stroke + Saline group in the same time period; Δ *p* < 0.05 denotes a significant difference from the previous observation time point based on a two-way ANOVA with Bonferroni’s post-hoc multiple comparison test.

One day after the induction of PT, the length of penumbra at the central site was 734 ± 42 μm, with 379 ± 28 μm in the lateral zone in the Stroke + Saline group (Figure 4 and Figure 5A). The number of neurons in the penumbra decreased by 20% compared to the relevant sites in the Control group, while the counts of macrophages and neutrophils increased 3- and 10-fold, respectively (*p* < 0.05; Figure 4 and Figure 5B–D, Figure S1). A single administration of Mexidol 2 h after PT resulted in a decrease of the penumbra adjacent to the central and lateral regions of the infarct area by 40% and 60% (*p* < 0.05), whereas Semax reduced those regions by 15% and 25%, respectively, compared to the Stroke + Saline group (*p* < 0.05; Figure 4 and Figure 5A). The treatment with drugs was not accompanied by any alterations in the number of penumbral neurons compared to the Control group. The infiltration of the penumbral zone by macrophages and neutrophils decreased 2- and 3-fold in the Stroke + Mexidol group and by 25% and 15% in the Stroke + Semax group, respectively, compared to the Stroke + Saline group (*p* < 0.05; Figure 4 and Figure 5C,D, Figures S2 and S3).

Three days after PT in the Stroke + Saline group, the extension of the penumbra increased by 30% (*p* < 0.05) in its central site and by 50% (*p* < 0.05) in the lateral sites adjacent to the necrotic zone compared to the first postoperative day. Moreover, the number of neurons decreased by 10% (*p* < 0.05), and the infiltration by macrophages and neutrophils increased by 15% and 2-fold, respectively (*p* < 0.05; Figure 4 and Figure 5). These alterations were coupled with the observed microcirculatory degenerative signs: Pronounced vasodilatation, erythrocyte stasis, an increase in blood vessel permeability, and multiple hemorrhages (Figure 4 and Figure S1). In the Stroke + Mexidol group at 3 days of treatment, the length of the penumbra increased by 15% (*p* < 0.05) compared to the results after 1 day in the same group. In rats treated by Semax, the treatment increased the central and lateral regions by 20% and 40%, respectively (*p* < 0.05). The size of the penumbra in the rats receiving treatment significantly decreased by 30% in the Stroke + Mexidol group and by 20% in the Stroke + Semax group compared to the Stroke + Saline group (Figure 5A). The number of neurons in the penumbra after a 3-day treatment of both Mexidol and Semax did not differ from that of the Control group. The intragroup comparison of macrophage and neutrophil counts in the penumbra between 1 and 3 days in the treated groups revealed average increases of 25%–30% and 2-fold (*p* < 0.05), respectively. Compared to the Stroke + Saline group, the macrophage and the neutrophil infiltration of the penumbra was statistically significantly lower by 2-fold (*p* < 0.05) only in the Stroke + Mexidol group (Figure 4 and Figure 5, Figures S2 and S3).

Further observations into the Stroke + Saline group after 7 postoperative days revealed the progress of microvascular degeneration related to the extension of the penumbral outer border by 50% and 20% in the central and the lateral regions, respectively, compared to the observations at 3 days (*p* < 0.05). We also observed an increase in neutrophil infiltration of the penumbra by 30% (*p* < 0.05), whereas the counts of neurons and macrophages did not change significantly compared to the 3-day endpoint (Figure 4 and Figure 5, Figure S1). The completed 7-day course of treatment with Mexidol was accompanied by a restriction in the spread of the penumbral zone and the absence of an increase in the quantity of macrophage and neutrophil infiltration compared to the 3-day post-stroke period. However, in animals treated with Semax, there was an increase in the size of the penumbra by 40% and 15% for central and lateral areas, respectively, and a 20% increase in neutrophil infiltration (*p* < 0.05) in comparison with the results at 3 days (Figure 4 and Figure 5). A comparison of Stroke + Mexidol and Stroke + Semax with the Stroke + Saline group revealed a decrease in the length of the penumbral zone by 60% and 30% (*p* < 0.05), respectively. The number of neurons in the penumbra was 30% higher (*p* < 0.05) in both treated groups, the number of macrophages decreased by 50% in the Mexidol-treated group and 25% in the Semax-treated group, and the number of neutrophils decreased by 2.5-fold only in the Stroke + Mexidol group (*p* < 0.05; Figure 4 and Figure 5, Figures S2 and S3).

On Day 21 after PT induction, in the Stroke + Saline group, we observed a 40% reduction in the transitional zone between the intact tissue and the infarct core (*p* < 0.05), a 30% and 80% decrease in the number of macrophages (*p* < 0.05) and neutrophils, respectively (*p* < 0.05). Moreover, the number of neurons did not change in comparison with Day 7 of the post-ischemic period (Figure 4 and Figure 5, Figure S1). On Day 14 after Mexidol treatment (Postoperative Day 21), the penumbra was reduced by 50%, the number of neurons and macrophages remained unchanged, and the number of neutrophils decreased by 85% compared to Day 7 of the post-ischemic period. At the same time point in the Stroke + Semax group, the penumbra size decreased by 40% (*p* < 0.05), the number of neurons and macrophages remained unchanged, and the number of neutrophils decreased by 80% compared to Observation Day 7. The macrophage and neutrophil counts were 30% and 70% lower, respectively (*p* < 0.05), in the Stroke + Mexidol group compared to the Stroke + Saline group but not in the Stroke + Semax group. The number of neurons was higher by 40% (*p* < 0.05) in the drug-treated groups compared to the rats given PT treated with saline (Figure 4 and Figure 5, Figures S2 and S3).

Thus, the use of a 7-day course of Mexidol and Semax treatment was associated with the preservation of neurons in the penumbra. Mexidol showed greater protective efficiency, which may be related to the pronounced restriction of penumbral infiltration by pro-inflammatory cells, whose cytotoxic activity triggers the mechanisms of neuronal death.

### 3.2. The Influence of Mexidol and Semax Treatment on the Expression and Activity of PGC-1α in the Penumbral Zone

**Figure 6:**
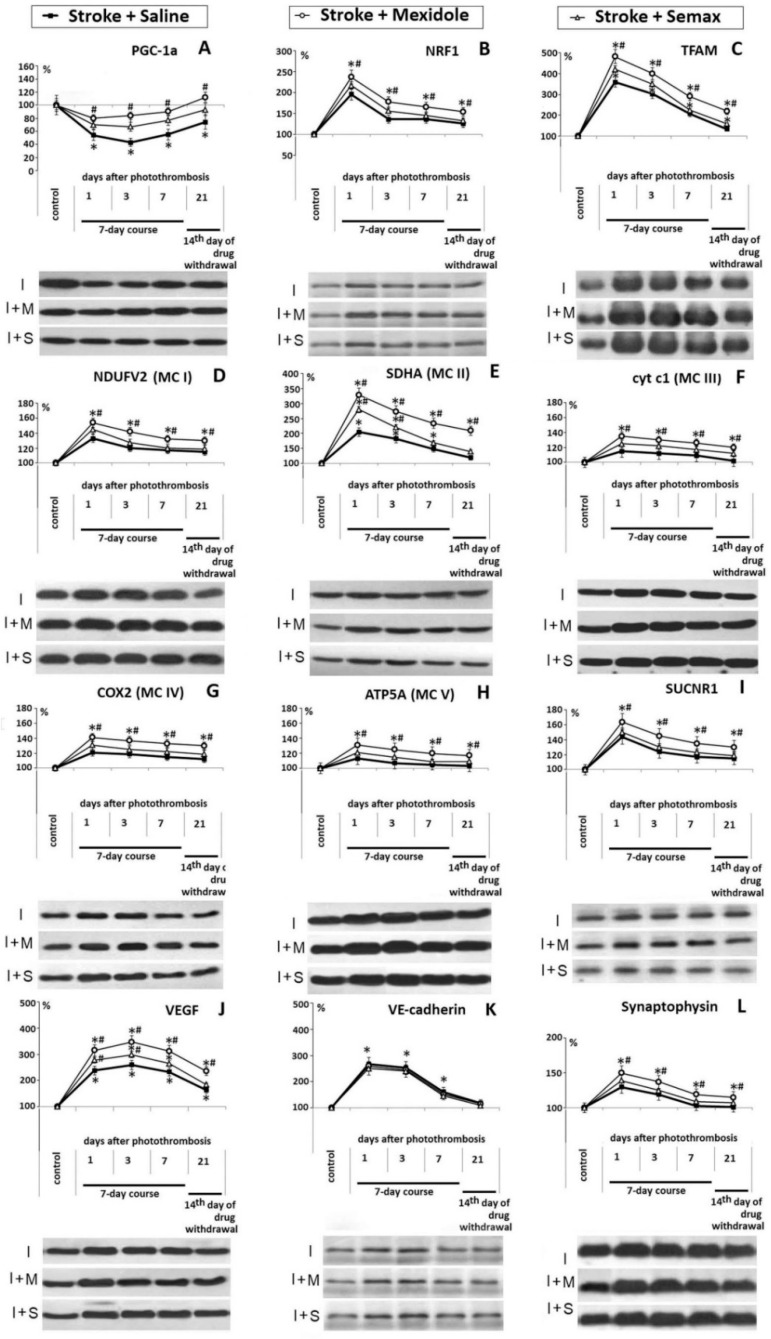
Influence of a 7-day course of Mexidol and Semax treatment on PGC-1α expression and the levels of protein markers of PGC-1α activity in the penumbral zone for 21 days after ischemia. The diagrams represent the results of a densitometry analysis, expressed in percentages, of the corresponding protein bands on the Western blot images. Representative immunoblots of PGC-1α (**A**), NRF1 (**B**), TFAM (**C**), NDUFV2 (**D**), SDHA (**E**), cyt c1 (**F**), COX2 (**G**), ATP5A (**H**), SUCNR1 (**I**), VEGF (**J**), VE-cadherin (**K**), and Synaptophysin (**L**) target proteins are included in the figure. The number of animals used for Western blot densitometry was *n* = 3 per each experimental episode. * *p* < 0.01 denotes a significant difference from the Control group; # *p* < 0.01 denotes a significant difference from the Stroke + Saline group; statistical analysis: A two-way ANOVA with Bonferroni’s post-hoc multiple comparison test. I (Ischemia): Stroke + Saline group; I + M: Stroke + Mexidol group; I + S: Stroke + Semax group.

The focal ischemia was accompanied by a decrease of the PGC-1α protein level in the cytoplasmic extract of the tissue from the penumbral zone at 1, 3, 7, and 21 days (Figure 6A). A maximal decrease in the PGC-1α level by 50% (*p* < 0.01) was detected on Days 1 and 3 of the post-ischemic period compared to the Control group. This decrease might be due to the following causes: (1) A reduction in the number of neurons expressing PGC-1α in the penumbral zone, as shown in Section 2.1, (2) a reduction in PGC-1α synthesis in the remaining neurons of the penumbra, and/or (3) translocation of the activated PGC-1α into the neuronal nuclei and a decrease of its level in the cytoplasm. Indeed, the PGC-1α activity peaked at 1 day after ischemia, which was evidenced by the increased levels of PGC-1α-dependent proteins (Figure 6B–H,J,L). The most significant increase was observed for transcription factors NRF1 (200%; *p* < 0.01; Figure 6B) and TFAM (350%; *p* < 0.01; Figure 6C), catalytic subunits of the substrate binding sites of the respiratory chain NDUFV2 (140%; *p* < 0.05; Figure 6D) and SDHA (200%; *p* < 0.01; Figure 6E), VEGF (250%; *p* < 0.01; Figure 6J), and SYP (130%; *p* < 0.01; Figure 6L), which are widely used as markers of PGC-1α activation [72]. The content of the catalytic subunits of the cytochrome-containing site in the mitochondrial respiratory chain (cyt c1, COX2) and ATP-synthase (ATP5A) increased by 20% (*p* < 0.01; Figure 6F–H). The PGC-1α activity was increased in conjunction with the upregulation of succinate receptor SUCNR1 expression (Figure 6I).

By Days 7 and 21 of the post-ischemic period, the content of the PGC-1α activity marker proteins gradually decreased, reaching the Control group level by the end of the observations (Figure 6). In the same time period, the level of PGC-1α in the cytosol extract of the penumbra was increased by 10% (Day 7) and 20% (*p* < 0.01, Day 21), which may suggest a decrease in PGC-1α translocation to the nuclei of neurons, given its reduced activation.

In the Stroke + Mexidol group, 24 h after ischemia induction, the level of PGC-1α expression in the penumbral tissue was comparable to that in the control animals, which was 30% higher than the PGC-1α expression level in the Stroke + Saline group (*p* < 0.01; Figure 6A). The effect of maintaining the PGC-1α expression at the Control group level persisted throughout the 7-day therapeutic course and 14 days after withdrawal of the drug (Figure 6A). The Mexidol course was accompanied by an increase of PGC-1α-dependent proteins content in the penumbral tissues, and the expression of these proteins was significantly higher than that in the Stroke + Saline group (Figure 6). The most pronounced effects of Mexidol on the PGC-1α activity were observed over the 7 days of the course, where we observed a significant increase of 40% in the NRF1 expression level (*p* < 0.01), 100% in TFAM (*p* < 0.01), 30% in NDUFV2 (*p* < 0.01), 100% in SDHA (*p* < 0.01), 80% in VEGF (*p* < 0.01), and 20% in SYP (*p* < 0.01) compared to the Stroke + Saline group. The expression levels of cyt c1, COX2, ATP5A, and SUCNR1 were also significantly elevated by 20% (*p* < 0.01) compared to the Stroke + Saline group (*p* < 0.01; Figure 6).

In the Stroke + Semax group, analogous to the effects of Mexidol, we observed a statistically significantly increased level of PGC-1α (by 20%, *p* < 0.01) compared to the Stroke + Saline group in the acute and delayed post-ischemic periods (Figure 6A). The activity of PGC-1α, estimated by the levels of PGC-1α-dependent proteins, was substantially higher compared to that in the Stroke + Saline group, but not as pronounced as that in the Stroke + Mexidol group. The effects of Semax on the PGC-1α activity were manifested within 3 days of the post-ischemic period. The significant increase of NRF1 expression level was 20% (*p* < 0.01), that of TFAM was 50% (*p* < 0.01), that of NDUFV2 was 20% (*p* < 0.01), that of SDHA was 50% (*p* < 0.01), and that of VEGF was 40% (*p* < 0.01), compared to the Stroke + Saline group (Figure 6).

In the present research, we also studied the dynamics of the expression of proteins associated with angiogenesis and synaptic plasticity, such as VEGF, VE-cadherin, and synaptophysin. VE-cadherin (VEC) and VEGF play a crucial role in the angiogenesis, stabilization, and maturation of newly formed blood vessels [73,74]. VEGF expression is controlled by PGC-1α, as well as by HIF-1α, one of the crucial factors that determines the acquisition of the adaptive reaction in the brain to ischemia [21,75,76,77]. VEC expression, unlike that of VEGF, is not PGC-1α-dependent and is regulated explicitly by HIF-1α [75,78,79]. The studied drugs did not influence the expression of VE-cadherin but statistically significantly increased VEGF levels (Figure 6). In the Stroke + Mexidol group, the synaptophysin level in the penumbra was increased compared to that in the Stroke + Saline group but not that in the Stroke + Semax group (*p* < 0.01, Figure 6).

### 3.3. Effects of Mexidol and Semax Courses on the Expression and Nuclear Translocation of PGC-1α in the Neurons of the Penumbral Zone

**Figure 7:**
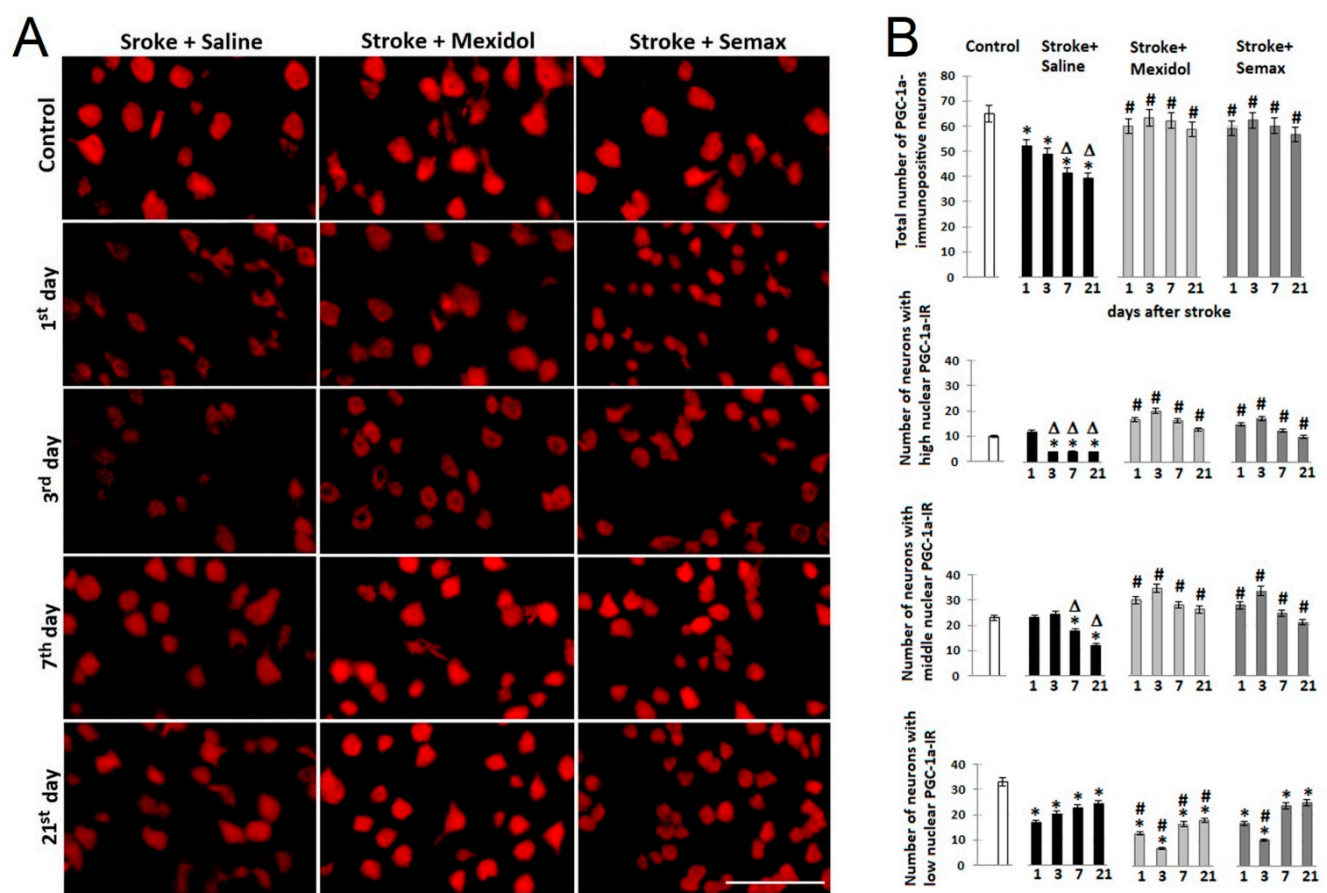
Effect of a 7-day treatment course of Mexidol and Semax on the expression and nuclear localization of PGC-1α in penumbral neurons at 1, 3, 7, and 21 days after PT of the prefrontal cortex. (**A**) Representative microphotographs of the immunohistochemical staining of rat prefrontal cortex slices. Bar: 50 μm. (**B**) Diagrams showing the number of PGC-1α-immunopositive neurons and neurons with high, middle, and low nuclear PGC-1α-immunoreactivity. The mean values of the number of cells were obtained by counting six randomized micrographs (images corresponded to the area of a penumbra 0.1 mm^2^) in three different sections from four rats for each experimental episode; data represent the mean ± SEM. * *p* < 0.05 denotes a significant difference from the Control group; # *p* < 0.05 denotes a significant difference from the Stroke + Saline group; Δ *p* < 0.05 denotes a significant difference from the first postoperative day (intra-group comparisons) based on a two-way ANOVA with Bonferroni’s post-hoc multiple comparison test. PGC-1α-IR: PGC-1α-immunoreactivity.

To estimate the expression levels of PGC-1α in individual neurons and the PGC-1α levels in neuronal nuclei (which reflect PGC-1α activation), we used immunohistochemical staining of the prefrontal cortex slices with the subsequent analysis of immunoreactive (IR) neurons in the penumbra. In the brain slices of the Control group, the overall number of neurons with high and medium nuclear PGC-1α-immunoreactivity was 15% and 35% of the total number of IR-neurons, respectively (Figure 7). In the Stroke + Saline group, 24 h after PT induction, the total number of PGC-1α-immunopositive neurons decreased by 20% (*p* < 0.05; Figure 7A,B, Figure S28), while the number of neurons with high and medium nuclear PGC-1α-immunoreactivity did not change. Nevertheless, the proportions of such neurons among the total number of PGC-1α-expressing neurons increased by 7% and 10%, respectively. The changes in the quantitative ratio of neurons differing at the level of PGC-1α expression indicated the early (within 24 h) post-ischemic death of neurons with low nuclear PGC-1α immunoreactivity, which are potentially the least resistant neurons to ischemia.

In the Stroke + Mexidol and Stroke + Semax groups, within 24 h after ischemia, the number of PGC-1α-immunopositive neurons in the penumbra remained on a level comparable to that of the Control group but 15% higher than that in the Stroke + Saline group (*p* < 0.05). However, in the Stroke + Mexidol group, the numbers of neurons with high and medium nuclear PGC-1α immunoreactivity were higher by 40% and 30% (*p* < 0.05), respectively, and those in the Stroke + Semax group were higher by 25% and 20% (*p* < 0.05) in comparison to the Stroke + Saline group (Figure 7A,B, Figures S29 and S30). Therefore, the treatment with Mexidol and Semax contributed to maintaining the total number of PGC-1α-expressing neurons at the control level, as well as to a significant increase in the proportion of neurons with high and medium nuclear PGC-1α immunoreactivity—i.e., PGC-1α activity.

Three days after PT, the total number of PGC-1α-immunopositive neurons decreased by 10%, and the number of neurons with high nuclear PGC-1α immunoreactivity decreased by 70% (*p* < 0.05) compared to the first day after stroke modeling in the Stroke + Saline group (Figure 7A,B, Figure S28). A 3-day course of Mexidol and Semax treatment did not significantly affect the total number of PGC-1α-immunopositive neurons, neurons with high and medium nuclear PGC-1α-immunoreactivity, compared to a single administration of drugs, but their numbers were higher by 30%, 4- and 1.4-fold, respectively, compared to those in the Stroke + Saline group (*p* < 0.05; Figure 7A,B, Figures S29 and S30).

At Days 7 and 21 after PT in the Stroke + Saline group, the total number of PGC-1α-expressing neurons and neurons with medium nuclear PGC-1α immunoreactivity progressively decreased and, at Day 21, achieved 75% and 50% (*p* < 0.05), respectively, compared to Day 1 after ischemia induction (Figure 7A,B, Figure S28). The steady post-ischemic (Days 3–21) decrease in the levels of active nuclear PGC-1α, mediating multiple neuroprotector mechanisms, was coupled with progressive neuronal death in the penumbral zone within 21 days after the stroke (see Section 3.1). Animals treated with Mexidol or Semax had significantly more neurons with high nuclear PGC-1α immunoreactivity in the penumbra: 3-fold more in the Stroke + Mexidol group and 2.5-fold more in the Stroke + Semax group compared to the Stroke + Saline group at 7 days postoperation, as well as at 21 days. The total number of PGC-1α-expressing neurons was also significantly higher in the Stroke + Mexidol and Stroke + Semax groups at Day 21 of the observations: 1.5-fold and 1.4-fold (*p* < 0.05) increases, respectively, compared to the Stroke + Saline group (Figure 7A,B, Figures S29 and S30).

Thus, the 7-day course of Mexidol and Semax treatment increased the expression and stimulated the nuclear translocation (activation) of PGC-1α, which was associated with the survival of penumbral neurons.

## 4. Discussion

The present study revealed novel effective and safe pharmacological modulators of the transcription coactivator PGC-1α, which determines increases in cellular energy production, neuron functionality, and viability [2,5,17,80]. We are the first to show that the succinate-containing drug Mexidol (2-ethyl-6-methyl-3-hydroxypyridine succinate) and the peptide mimetic of ACTH (4–7), Semax, activate and induce PGC-1α in the penumbral neurons in a model of focal ischemia and exert neuroprotective effects through these mechanisms.

Mexidol and Semax have been studied as potential positive modulators of PGC-1α since, despite their different chemical natures, they exhibit similar patterns of neuroprotective effects, such as reduced brain damage, cognitive and sensorimotor deficits, and the ability to exert anti-exitotoxic, antioxidant, anti-inflammatory, and neurotrophic effects [45,47,48,49,50,55,56,57,58,81]. The multi-target neuroprotective activity of these drugs suggests that they have a common intracellular target molecule—the transcriptional coactivator PGC-1α, known for its pleiotropic potentiating effect on neuron viability and functionality.

This study was performed on a model of photochemically induced thrombosis of prefrontal cortex blood vessels, mimicking the pathogenesis of acute ischemic stroke, which accounts for 85% of acute cerebrovascular disease incidence, according to recent estimates [1]. Using the methods of Western blot analysis and immunohistochemistry, we revealed a persistent suppression of PGC-1α in the penumbra, which was detected throughout the 21-day post-ischemic period and reflected a progressive decrease in the number of PGC-1α-expressing neurons and PGC-1α levels in individual neurons.

The mechanism of suppression of PGC-1α expression in long-term ischemia/hypoxia has been intensively studied. The sustained suppression of PGC-1α in ischemic brain tissue without reperfusion was reported in models of permanent occlusion in the middle cerebral artery, in an endothelin model of stroke, and in chronic cerebral hypoperfusion [82,83]. The hypoxia-inducible transcription factor HIF-1α plays a primary role in the negative regulation of PGC-1α [84]. It is known that HIF-1α is a critical factor in the formation of gene-mediated adaptation mechanisms under conditions of oxygen deficiency, including angiogenesis, the glycolytic oxidation pathway, and erythropoiesis [85]. HIF-1α is also a key factor in suppressing mitochondrial biogenesis and PGC-1α expression through mechanisms of negative regulation of the transcription factor MYC and the MYC-dependent transcription factors (PPARs and ERRs) that activate the PGC-1α gene [14,86,87]. Thus, conditions of prolonged ischemia/hypoxia cause HIF-1α-dependent negative regulation of PGC-1α and PGC-1α-related processes (mitochondrial biogenesis). In this regard, potential neuroprotective drugs should have vasodilator, angiogenic, and angioprotective effects, enhance the perfusion/oxygenation of the ischemic area of the brain, limit the activity of HIF-1α, and potentiate PGC-1α induction.

The treatment with Mexidol and Semax 2 h after PT, and for 6 subsequent days, was accompanied by a reduction in brain injury, maintaining the number of penumbral neurons and the level of PGC-1α expression in individual neurons in the penumbra at the control level, as well as stimulating the nuclear translocation (activation) of PGC-1α. Our findings are in line with previous data on the neuroprotective effects of Semax and Mexidol. Intranasal administration of Semax over the 7-day treatment course resulted in a 25% decrease of infarct volume in the prefrontal cortex and cognitive function recovery over the long term [45,81]. In the middle cerebral artery occlusion model, the delayed administration of Mexidol 6 h after ischemia induction and for 6 subsequent days resulted in a 30% decrease in brain damage [88].

The morphological analysis of histological prefrontal cortex slices of rats in the Stroke + Saline group revealed a significant tendency of increasing infiltration of the core ischemic zone and the penumbra by inflammatory immune cells (macrophages and neutrophils). Their cytotoxicity may have contributed to the progressive neuronal death (the mechanism of secondary inflammatory neuronal death) observed within 21 days after PT induction. Seven-day courses of Mexidol and Semax exerted an immunosuppressive effect by limiting the infiltration of the penumbra by inflammatory cells, as well as the dynamics and severity of the inflammatory response. Mexidol had a more pronounced effect: Its administration decreased the macrophage and neutrophil counts in the penumbra by 2- and 3-fold, respectively, over 21 days after the ischemia. The modulation of inflammation under the studied drugs may be mediated by their interactions with specific receptors (SUCNR1 for Mexidol and MCRs for Semax), expressed in the microglia, macrophages, and lymphocytes. Previously, the direct receptor-mediated anti-inflammatory effects of ACTH and succinate on immune cells associated with blockages of pro-inflammatory polarization have been shown [51,52,89]. The results of Filippenkov et al. are consistent with our data on the anti-inflammatory effects of Semax. Using RNA-Seq analysis, the authors showed that Semax suppressed the inflammatory pathways and activated the brain’s neurotransmitter signaling pathways after focal ischemia [48]. Maintaining the anti-inflammatory (reparative) M2 phenotype of macrophages and microglia is crucial for PGC-1α induction in penumbral neurons since the M2 phenotype is the main source of neurotrophins, which are positive modulators of PGC-1α [17,90].

The anti-inflammatory effects of these drugs, in conjunction with their angioprotective activities, have been developed. Indeed, on the third day after PT, signs of degenerative changes in the microcirculatory bed were detected, such as multiple hemorrhages, pronounced vasodilation, and erythrocyte stasis. Secondary post-stroke perfusion disturbances led to a significant progression of the penumbra, while the administration of Semax limited, and Mexidol eliminated, the disturbances of peri-infarction microcirculation observed after PT. The powerful angio- and neuroprotective effects of Mexidol may be related to Mexidol’s influence on the rheologic properties of blood due to the presence of phenolic antioxidant ethylmethylhydroxypyridine in the composition of the molecule [58]. Moreover, the effects of Semax on the anticoagulant, fibrinolytic, and platelet components of the anticoagulation system were reported [91]. Improvement of microcirculation and penumbral tissue oxygenation mediated by the drugs Mexidol and Semax may be crucial to reverse the HIF-1α-dependent blockade of PGC-1α expression [14,86,87].

The drugs’ abilities to exert neurotrophic effects may be mediated by the activation of specific receptors: SUCNR1 for Mexidol and receptors of the melanocortin family MCRs for Semax [51,52,59,60]. Receptor stimulation is accompanied by the activation of G proteins and intracellular signal cascades. Their key elements are kinases (CaMK, MAPK, and PI3K), which are known to exert multiple neuroprotective effects—in particular, the activation of transcriptional factor CREB, controlling the expression of anti-inflammatory (IL-10, TGF-β), antiapoptotic (Bcl–2), neurotrophic (BDNF), and antioxidant (SOD2) factors, as well as the transcriptional co-activator PGC-1α [30,33,92]. Indeed, immunohistochemical staining of the rat prefrontal cortex slices revealed high levels of neuronal PGC-1α expression in the Stroke + Mexidol and Stroke + Semax groups.

Semax and Mexidol treatment courses promoted the induction and activation of PGC-1α. In our stroke model, we observed a pronounced short-term PGC-1α activation, evidenced by a significant upregulation of PGC-1α-dependent proteins: NRF1, TFAM, catalytic subunits of the substrate-binding sites of the respiratory chain (NDUFV2 and SDHA), VEGF, and SYP. The highest level of protein markers for the PGC-1α activity was detected 24 h after PT, after which, the intensity of the induction of PGC-1α-dependent proteins decreased and disappeared by the 7th day. The putative activators of PGC-1α in the acute period of ischemia can be considered a flow of calcium ions initiated via hyperstimulation of the ionotropic glutamate receptors (NMDA and AMPA) [93] through the activation of Ca^2+^/CaM kinase, thereby phosphorylating and activating PGC-1α, as well as the products of necrotic cell degradation, known as damage-associated molecular patterns (DAMPs), which are recognized by the TLRs of neurons and other cells of the central nervous system [94,95]. During the post-ischemic period, when cell death slowed, the degree of PGC-1α activation also significantly declined. The treatment with Mexidol and Semax, especially Mexidol, prolonged the period of PGC-1α activation, which may be related to the receptor-mediated (by SUCNR1 and MCRs) initiation of calcium signaling cascades in neurons [51,52,59,60].

Based on these findings, we conclude that the succinate-containing drug Mexidol has a pronounced potential for PGC-1α induction and activation in the hypoperfusion area adjacent to the infarct core (the penumbra). Succinate can be described as an endogenous danger signal released by damaged cells and cells under the extreme conditions of ischemia/hypoxia. Succinate may be of interest in the development of low-molecular-weight DAMP mimetics that stimulate reparative processes, the development of which is being actively carried out by modern pharmacology [96,97]. Succinate is an energy substrate oxidized by the complex II of the mitochondrial respiratory chain. Under hypoxia conditions, succinate is intensely produced by mitochondria in the reverse Krebs cycle, which contributes to maintaining the pool of oxidized NAD^+^, NAD^+^-dependent glycolysis, and energy production by the mitochondria [98]. In 2004, when the highly specific succinate receptor SUCNR1/GPR91 was discovered, it became evident that succinate plays a regulatory signaling role in the formation of adaptive mechanisms of tolerance to ischemia/hypoxia at the cellular, tissue, and organismic levels [59,99,100,101]. The succinate/SUCNR1 signaling system activates angiogenic processes in the ischemic brain and exerts a vasodilatory effect [59]. Recent research shows that succinate is intensely produced by activated macrophages [102]. Therefore, succinate is a unique signaling adaptogenic molecule whose production significantly increases under conditions of ischemia/hypoxia, unlike peptide signaling molecules, whose synthesis is blocked by ischemia/hypoxia [103]. The ischemic suppression of translational processes is accompanied by the downregulation of peptide neurohormones and neurotransmitters, as well as their receptors [49,103]. This is why in the protection of penumbral cells, the priority role may belong to the non-peptide signaling molecules exerting adaptogenic effects through the activation of constitutively expressed receptors. The present study shows that ischemia is associated with elevated SUCNR1 levels in the penumbra. The administration of succinate-containing Mexidol resulted in more pronounced and prolonged SUCNR1 expression. We showed previously that a treatment course of Mexidol at a dose of 100 mg/kg elevates the expression of SUCNR1 in the brain cortex of healthy young and aged rats and yields elevated PGC-1α expression and activation [61]. Importantly, PGC-1α induction may not be related only to SUCNR1 activation in neurons. This effect can be realized indirectly through the mechanism of the SUCNR1-dependent increase in the cerebral secretion of catecholamines involved in PGC-1α induction or as a result of neurotrophin secretion by M2 macrophages/microglia also expressing SUCNR1 [89,104]. These mechanisms need further study.

## 5. Conclusions

This study is the first to demonstrate that sufficient PGC-1α induction/activation in the neurons located in the ischemic penumbra might be realized by the combined manifestation of anti-inflammatory, angioprotective, and neurotrophic effects of pharmacological agents, mediated by specific receptors (SUCNR1 for Mexidol and MCRs for Semax in the presented study). These properties are typical of the succinate/SUCNR1 signaling system. This study shows that succinate-containing drugs may be of particular interest as positive PGC-1α modulators.

## Data Availability

Not applicable.

## Supplementary Materials

The following are available online at https://www.mdpi.com/2076-3425/11/3/325/s1. Figure S1: Photomicrographs from sections of the prefrontal cortex of rats with photochemically induced thrombosis (PT) that received i/p saline injection 2 h after PT and for the consecutive 6 days (one injection per day) (“Stroke + Saline” experimental group). Hematoxylin and eosin staining. Lens x100. Sections show the distribution of resident cells of the nervous system and leukocytes recruited from the blood in the structure of the ischemic penumbra. Designations: N: Neuron; A: Astrocyte; M: Microglia; O: Oligodendrocyte; dN: Degenerating neuron; rA: Reactive astrocyte; n: Neutrophil; m: Monocyte/macrophage; Figure S2: Photomicrographs from sections of the prefrontal cortex of rats with photochemically induced thrombosis (PT) that were treated with Mexidol by i/p injection 2 h after PT and for the consecutive 6 days (one injection per day) at a dose of 100 mg/kg (“Stroke + Mexidol” experimental group). Hematoxylin and eosin staining. Lens x100. Sections show the distribution of resident cells of the nervous system and leukocytes recruited from the blood in the structure of the ischemic penumbra. Designations: N: Neuron; A: Astrocyte; M: Microglia; O: Oligodendrocyte; dN: Degenerating neuron; rA: Reactive astrocyte; n: Neutrophil; m: Monocyte/macrophage; Figure S3: Photomicrographs from sections of the prefrontal cortex of rats with photochemically induced thrombosis (PT) that were treated with Semax by i/n administration 2 h after PT and for the consecutive 6 days (one instillation per day) at a dose of 25 μg/kg (“Stroke + Semax” experimental group). Hematoxylin and eosin staining. Lens x100. Sections show the distribution of resident cells of the nervous system and leukocytes recruited from the blood in the structure of the ischemic penumbra. Designations: N: Neuron; A: Astrocyte; M: Microglia; O: Oligodendrocyte; dN: Degenerating neuron; rA: Reactive astrocyte; n: Neutrophil; m: Monocyte/macrophage; Figure S4: Representative immunoblot image (Rg-film combined with nitrocellulose membrane) showing expression of PGC-1α in the prefrontal cortex penumbra from rats with photochemically induced thrombosis (PT) (“Stroke + Saline” group), rats were treated with Mexidol by i/p injection 2 h after PT and for the consecutive 6 days (one injection per day) at a dose of 100 mg/kg (“Stroke + Mexidol” group), and intact rats were treated with a 7-day course of Mexidol, for 21 days of observation. Top sequence of blots (first gel; one animal corresponds to each blot), from left to right: 1,2: “Control” (rats received saline according to the same regimen as in the “Stroke + Mexidol” group); 3,4: “Stroke + Saline”, 24 h after PT; 5,6: “Stroke + Mexidol”, 24 h after PT; 7,8: Intact rats receiving a single injection of Mexidol; 9,10: “Stroke + Saline”, 3 days after PT; 11,12: “Stroke + Mexidol”; 3 days after PT; 13,14: Intact rats receiving a 3-day course of Mexidol. Bottom sequence of blots (second gel; one animal corresponds to each blot), from left to right: 1,2: “Control”; 3,4: “Stroke + Saline”, 7 days after PT; 5,6: “Stroke + Mexidol”, 7 days after PT; 7,8: Intact rats receiving a 7-day course of Mexidol; 9,10: “Stroke + Saline”, 21 days after PT; 11,12: “Stroke + Mexidol”, 14 days after withdrawal of Mexidol treatment; 13,14: Intact rats, 14 days after withdrawal of Mexidol treatment; Figure S5: Representative immunoblot image (Rg-film combined with nitrocellulose membrane) showing expression of PGC-1α in the prefrontal cortex penumbra from rats with photochemically induced thrombosis (PT) (“Stroke + Saline” group), rats were treated with Semax by i/n administration 2 h after PT and for the consecutive 6 days (one instillation per day) at a dose of 25 μg/kg (“Stroke + Semax” group), and intact rats were treated with a 7-day course of Semax, for 21 days of observation. Top sequence of blots (first gel; one animal corresponds to each blot), from left to right: 1,2: “Control” (rats received saline); 3,4: “Stroke + Saline”, 24 h after PT; 5,6: “Stroke + Semax”, 24 h after PT; 7,8: Intact rats receiving a single injection of Semax; 9,10: “Stroke + Saline”, 3 days after PT; 11,12: “Stroke + Semax”; 3 days after PT; 13,14: Intact rats receiving a 3-day course of Semax. Bottom sequence of blots (second gel; one animal corresponds to each blot), from left to right: 1,2: “Control”; 3,4: “Stroke + Saline”, 7 days after PT; 5,6: “Stroke + Semax”, 7 days after PT; 7,8: Intact rats receiving a 7-day course of Semax; 9,10: “Stroke + Saline”, 21 days after PT; 11,12: “Stroke + Semax”, 14 days after withdrawal of Semax treatment; 13,14: Intact rats, 14 days after withdrawal of Semax treatment; Figure S6: Representative immunoblot image (Rg-film combined with the nitrocellulose membrane) showing expression of NRF1 in the prefrontal cortex penumbra from rats with photochemically induced thrombosis (PT) (“Stroke + Saline” group), rats treated with Mexidol by i/p injection 2 h after PT and for the consecutive 6 days (one injection per day) at a dose of 100 mg/kg (“Stroke + Mexidol” group), and intact rats were treated with a 7-day course of Mexidol, for 21 days of observation. Top sequence of blots (first gel; one animal corresponds to each blot), from left to right: 1,2: “Control” (rats received saline according to the same regimen as in the “Stroke + Mexidol” group); 3,4: “Stroke + Saline”, 24 h after PT; 5,6: “Stroke + Mexidol”, 24 h after PT; 7,8: Intact rats receiving a single injection of Mexidol; 9,10: “Stroke + Saline”, 3 days after PT; 11,12: “Stroke + Mexidol”; 3 days after PT; 13,14: Intact rats receiving a 3-day course of Mexidol. Bottom sequence of blots (second gel; one animal corresponds to each blot), from left to right: 1,2: “Control”; 3,4: “Stroke + Saline”, 7 days after PT; 5,6: “Stroke + Mexidol”, 7 days after PT; 7,8: Intact rats receiving a 7-day course of Mexidol; 9,10: “Stroke + Saline”, 21 days after PT; 11,12: “Stroke + Mexidol”, 14 days after withdrawal of Mexidol treatment; 13,14: Intact rats, 14 days after withdrawal of Mexidol treatment; Figure S7: Representative immunoblot image (Rg-film combined with nitrocellulose membrane) showing expression of TFAM in the prefrontal cortex penumbra from rats with photochemically induced thrombosis (PT) (“Stroke + Saline” group), rats were treated with Mexidol by i/p injection 2 h after PT and for the consecutive 6 days (one injection per day) at a dose of 100 mg/kg (“Stroke + Mexidol” group), and intact rats treated with a 7-day course of Mexidol, for 21 days of observation. Top sequence of blots (first gel; one animal corresponds to each blot), from left to right: 1,2: “Control” (rats received saline according to the same regimen as in the “Stroke + Mexidol” group); 3,4: “Stroke + Saline”, 24 h after PT; 5,6: “Stroke + Mexidol”, 24 h after PT; 7,8: Intact rats receiving a single injection of Mexidol; 9,10: “Stroke + Saline”, 3 days after PT; 11,12: “Stroke + Mexidol”; 3 days after PT; 13,14: Intact rats receiving a 3-day course of Mexidol. Bottom sequence of blots (second gel; one animal corresponds to each blot), from left to right: 1,2: “Control”; 3,4: “Stroke + Saline”, 7 days after PT; 5,6: “Stroke + Mexidol”, 7 days after PT; 7,8: Intact rats receiving a 7-day course of Mexidol; 9,10: “Stroke + Saline”, 21 days after PT; 11,12: “Stroke + Mexidol”, 14 days after withdrawal of Mexidol treatment; 13,14: Intact rats, 14 days after withdrawal of Mexidol treatment; Figure S8: Representative immunoblot image (Rg-film combined with nitrocellulose membrane) showing expression of NDUFV2 in the prefrontal cortex penumbra from rats with photochemically induced thrombosis (PT) (“Stroke + Saline” group), rats treated with Mexidol by i/p injection 2 h after PT and for the consecutive 6 days (one injection per day) at a dose of 100 mg/kg (“Stroke + Mexidol” group), and intact rats treated with a 7-day course of Mexidol, for 21 days of observation. Top sequence of blots (first gel; one animal corresponds to each blot), from left to right: 1,2: “Control” (rats received saline according to the same regimen as in the “Stroke + Mexidol” group); 3,4: “Stroke + Saline”, 24 h after PT; 5,6: “Stroke + Mexidol”, 24 h after PT; 7,8: Intact rats receiving a single injection of Mexidol; 9,10: “Stroke + Saline”, 3 days after PT; 11,12: “Stroke + Mexidol”; 3 days after PT; 13,14: Intact rats receiving a 3-day course of Mexidol. Bottom sequence of blots (second gel; one animal corresponds to each blot), from left to right: 1,2: “Control”; 3,4: “Stroke + Saline”, 7 days after PT; 5,6: “Stroke + Mexidol”, 7 days after PT; 7,8: Intact rats receiving a 7-day course of Mexidol; 9,10: “Stroke + Saline”, 21 days after PT; 11,12: “Stroke + Mexidol”, 14 days after withdrawal of Mexidol treatment; 13,14: Intact rats, 14 days after withdrawal of Mexidol treatment; Figure S9: Representative immunoblot image (Rg-film combined with nitrocellulose membrane) showing expression of SDHA in the prefrontal cortex penumbra from rats with photochemically induced thrombosis (PT) (“Stroke + Saline” group), rats treated with Mexidol by i/p injection 2 h after PT and for the consecutive 6 days (one injection per day) at a dose of 100 mg/kg (“Stroke + Mexidol” group), and intact rats treated with a 7-day course of Mexidol, for 21 days of observation. Top sequence of blots (first gel; one animal corresponds to each blot), from left to right: 1,2: “Control” (rats received saline according to the same regimen as in the “Stroke + Mexidol” group); 3,4: “Stroke + Saline”, 24 h after PT; 5,6: “Stroke + Mexidol”, 24 h after PT; 7,8: Intact rats receiving a single injection of Mexidol; 9,10: “Stroke + Saline”, 3 days after PT; 11,12: “Stroke + Mexidol”; 3 days after PT; 13,14: Intact rats receiving a 3-day course of Mexidol. Bottom sequence of blots (second gel; one animal corresponds to each blot), from left to right: 1,2: “Control”; 3,4: “Stroke + Saline”, 7 days after PT; 5,6: “Stroke + Mexidol”, 7 days after PT; 7,8: Intact rats receiving a 7-day course of Mexidol; 9,10: “Stroke + Saline”, 21 days after PT; 11,12: “Stroke + Mexidol”, 14 days after withdrawal of Mexidol treatment; 13,14: Intact rats, 14 days after withdrawal of Mexidol treatment; Figure S10: Representative immunoblot image (Rg-film combined with nitrocellulose membrane) showing expression of cyt c1 in the prefrontal cortex penumbra from rats with photochemically induced thrombosis (PT) (“Stroke + Saline” group), rats treated with Mexidol by i/p injection 2 h after PT and for the consecutive 6 days (one injection per day) at a dose of 100 mg/kg (“Stroke + Mexidol” group), and intact rats treated with a 7-day course of Mexidol, for 21 days of observation. Top sequence of blots (first gel; one animal corresponds to each blot), from left to right: 1,2: “Control” (rats received saline according to the same regimen as in the “Stroke + Mexidol” group); 3,4: “Stroke + Saline”, 24 h after PT; 5,6: “Stroke + Mexidol”, 24 h after PT; 7,8: Intact rats receiving a single injection of Mexidol; 9,10: “Stroke + Saline”, 3 days after PT; 11,12: “Stroke + Mexidol”; 3 days after PT; 13,14: Intact rats receiving a 3-day course of Mexidol. Bottom sequence of blots (second gel; one animal corresponds to each blot), from left to right: 1,2: “Control”; 3,4: “Stroke + Saline”, 7 days after PT; 5,6: “Stroke + Mexidol”, 7 days after PT; 7,8: Intact rats receiving a 7-day course of Mexidol; 9,10: “Stroke + Saline”, 21 days after PT; 11,12: “Stroke + Mexidol”, 14 days after withdrawal of Mexidol treatment; 13,14: Intact rats, 14 days after withdrawal of Mexidol treatment; Figure S11: Representative immunoblot image (Rg-film combined with nitrocellulose membrane) showing expression of COX2 in the prefrontal cortex penumbra from rats with photochemically induced thrombosis (PT) (“Stroke + Saline” group), rats treated with Mexidol by i/p injection 2 h after PT and for the consecutive 6 days (one injection per day) at a dose of 100 mg/kg (“Stroke + Mexidol” group), and intact rats treated with a 7-day course of Mexidol, for 21 days of observation. Top sequence of blots (first gel; one animal corresponds to each blot), from left to right: 1,2: “Control” (rats received saline according to the same regimen as in the “Stroke + Mexidol” group); 3,4: “Stroke + Saline”, 24 h after PT; 5,6: “Stroke + Mexidol”, 24 h after PT; 7,8: Intact rats receiving a single injection of Mexidol; 9,10: “Stroke + Saline”, 3 days after PT; 11,12: “Stroke + Mexidol”; 3 days after PT; 13,14: Intact rats receiving a 3-day course of Mexidol. Bottom sequence of blots (second gel; one animal corresponds to each blot), from left to right: 1,2: “Control”; 3,4: “Stroke + Saline”, 7 days after PT; 5,6: “Stroke + Mexidol”, 7 days after PT; 7,8: Intact rats receiving a 7-day course of Mexidol; 9,10: “Stroke + Saline”, 21 days after PT; 11,12: “Stroke + Mexidol”, 14 days after withdrawal of Mexidol treatment; 13,14: Intact rats, 14 days after withdrawal of Mexidol treatment; Figure S12: Representative immunoblot image (Rg-film combined with nitrocellulose membrane) showing expression of ATP5A in the prefrontal cortex penumbra from rats with photochemically induced thrombosis (PT) (“Stroke + Saline” group), rats treated with Mexidol by i/p injection 2 h after PT and for the consecutive 6 days (one injection per day) at a dose of 100 mg/kg (“Stroke + Mexidol” group), and intact rats treated with a 7-day course of Mexidol, for 21 days of observation. Top sequence of blots (first gel; one animal corresponds to each blot), from left to right: 1,2: “Control” (rats received saline according to the same regimen as in the “Stroke + Mexidol” group); 3,4: “Stroke + Saline”, 24 h after PT; 5,6: “Stroke + Mexidol”, 24 h after PT; 7,8: Intact rats receiving a single injection of Mexidol; 9,10: “Stroke + Saline”, 3 days after PT; 11,12: “Stroke + Mexidol”; 3 days after PT; 13,14: Intact rats receiving a 3-day course of Mexidol. Bottom sequence of blots (second gel; one animal corresponds to each blot), from left to right: 1,2: “Control”; 3,4: “Stroke + Saline”, 7 days after PT; 5,6: “Stroke + Mexidol”, 7 days after PT; 7,8: Intact rats receiving a 7-day course of Mexidol; 9,10: “Stroke + Saline”, 21 days after PT; 11,12: “Stroke + Mexidol”, 14 days after withdrawal of Mexidol treatment; 13,14: Intact rats, 14 days after withdrawal of Mexidol treatment; Figure S13: Representative immunoblot image (Rg-film combined with nitrocellulose membrane) showing expression of SUCNR1 in the prefrontal cortex penumbra from rats with photochemically induced thrombosis (PT) (“Stroke + Saline” group), rats treated with Mexidol by i/p injection 2 h after PT and for the consecutive 6 days (one injection per day) at a dose of 100 mg/kg (“Stroke + Mexidol” group), and intact rats treated with a 7-day course of Mexidol, for 21 days of observation. Top sequence of blots (first gel; one animal corresponds to each blot), from left to right: 1,2: “Control” (rats received saline according to the same regimen as in the “Stroke + Mexidol” group); 3,4: “Stroke + Saline”, 24 h after PT; 5,6: “Stroke + Mexidol”, 24 h after PT; 7,8: Intact rats receiving a single injection of Mexidol; 9,10: “Stroke + Saline”, 3 days after PT; 11,12: “Stroke + Mexidol”; 3 days after PT; 13,14: Intact rats receiving a 3-day course of Mexidol. Bottom sequence of blots (second gel; one animal corresponds to each blot), from left to right: 1,2: “Control”; 3,4: “Stroke + Saline”, 7 days after PT; 5,6: “Stroke + Mexidol”, 7 days after PT; 7,8: Intact rats receiving a 7-day course of Mexidol; 9,10: “Stroke + Saline”, 21 days after PT; 11,12: “Stroke + Mexidol”, 14 days after withdrawal of Mexidol treatment; 13,14: Intact rats, 14 days after withdrawal of Mexidol treatment; Figure S14: Representative immunoblot image (Rg-film combined with nitrocellulose membrane) showing expression of VEGF in the prefrontal cortex penumbra from rats with photochemically induced thrombosis (PT) (“Stroke + Saline” group), rats treated with Mexidol by i/p injection 2 h after PT and for the consecutive 6 days (one injection per day) at a dose of 100 mg/kg (“Stroke + Mexidol” group), and intact rats treated with a 7-day course of Mexidol, for 21 days of observation. Top sequence of blots (first gel; one animal corresponds to each blot), from left to right: 1,2: “Control” (rats received saline according to the same regimen as in the “Stroke + Mexidol” group); 3,4: “Stroke + Saline”, 24 h after PT; 5,6: “Stroke + Mexidol”, 24 h after PT; 7,8: Intact rats receiving a single injection of Mexidol; 9,10: “Stroke + Saline”, 3 days after PT; 11,12: “Stroke + Mexidol”; 3 days after PT; 13,14: Intact rats receiving a 3-day course of Mexidol. Bottom sequence of blots (second gel; one animal corresponds to each blot), from left to right: 1,2: “Control”; 3,4: “Stroke + Saline”, 7 days after PT; 5,6: “Stroke + Mexidol”, 7 days after PT; 7,8: Intact rats receiving a 7-day course of Mexidol; 9,10: “Stroke + Saline”, 21 days after PT; 11,12: “Stroke + Mexidol”, 14 days after withdrawal of Mexidol treatment; 13,14: Intact rats, 14 days after withdrawal of Mexidol treatment; Figure S15: Representative immunoblot image (Rg-film combined with nitrocellulose membrane) showing expression of VE-cadherin in the prefrontal cortex penumbra from rats with photochemically induced thrombosis (PT) (“Stroke + Saline” group), rats treated with Mexidol by i/p injection 2 h after PT and for the consecutive 6 days (one injection per day) at a dose of 100 mg/kg (“Stroke + Mexidol” group), and intact rats treated with a 7-day course of Mexidol, for 21 days of observation. Top sequence of blots (first gel; one animal corresponds to each blot), from left to right: 1,2: “Control” (rats received saline according to the same regimen as in the “Stroke + Mexidol” group); 3,4: “Stroke + Saline”, 24 h after PT; 5,6: “Stroke + Mexidol”, 24 h after PT; 7,8: Intact rats receiving a single injection of Mexidol; 9,10: “Stroke + Saline”, 3 days after PT; 11,12: “Stroke + Mexidol”; 3 days after PT; 13,14: Intact rats receiving a 3-day course of Mexidol. Bottom sequence of blots (second gel; one animal corresponds to each blot), from left to right: 1,2: “Control”; 3,4: “Stroke + Saline”, 7 days after PT; 5,6: “Stroke + Mexidol”, 7 days after PT; 7,8: Intact rats receiving a 7-day course of Mexidol; 9,10: “Stroke + Saline”, 21 days after PT; 11,12: “Stroke + Mexidol”, 14 days after withdrawal of Mexidol treatment; 13,14: Intact rats, 14 days after withdrawal of Mexidol treatment; Figure S16: Representative immunoblot image (Rg-film combined with nitrocellulose membrane) showing expression of Synaptophysin in the prefrontal cortex penumbra from rats with photochemically induced thrombosis (PT) (“Stroke + Saline” group), rats treated with Mexidol by i/p injection 2 h after PT and for the consecutive 6 days (one injection per day) at a dose of 100 mg/kg (“Stroke + Mexidol” group), and intact rats treated with a 7-day course of Mexidol, for 21 days of observation. Top sequence of blots (first gel; one animal corresponds to each blot), from left to right: 1,2: “Control” (rats received saline according to the same regimen as in the “Stroke + Mexidol” group); 3,4: “Stroke + Saline”, 24 h after PT; 5,6: “Stroke + Mexidol”, 24 h after PT; 7,8: Intact rats receiving a single injection of Mexidol; 9,10: “Stroke + Saline”, 3 days after PT; 11,12: “Stroke + Mexidol”; 3 days after PT; 13,14: Intact rats receiving a 3-day course of Mexidol. Bottom sequence of blots (second gel; one animal corresponds to each blot), from left to right: 1,2: “Control”; 3,4: “Stroke + Saline”, 7 days after PT; 5,6: “Stroke + Mexidol”, 7 days after PT; 7,8: Intact rats receiving a 7-day course of Mexidol; 9,10: “Stroke + Saline”, 21 days after PT; 11,12: “Stroke + Mexidol”, 14 days after withdrawal of Mexidol treatment; 13,14: Intact rats, 14 days after withdrawal of Mexidol treatment; Figure S17: Representative immunoblot image (Rg-film combined with nitrocellulose membrane) showing expression of NRF1 in the prefrontal cortex penumbra from rats with photochemically induced thrombosis (PT) (“Stroke + Saline” group), rats treated with Semax by i/n administration 2 h after PT and for the consecutive 6 days (one instillation per day) at a dose of 25 μg/kg (“Stroke + Semax” group), and intact rats treated with a 7-day course of Semax, for 21 days of observation. Top sequence of blots (first gel; one animal corresponds to each blot), from left to right: 1,2: “Control” (rats received saline); 3,4: “Stroke + Saline”, 24 h after PT; 5,6: “Stroke + Semax”, 24 h after PT; 7,8: Intact rats receiving a single injection of Semax; 9,10: “Stroke + Saline”, 3 days after PT; 11,12: “Stroke + Semax”; 3 days after PT; 13,14: Intact rats receiving a 3-day course of Semax. Bottom sequence of blots (second gel; one animal corresponds to each blot), from left to right: 1,2: “Control”; 3,4: “Stroke + Saline”, 7 days after PT; 5,6: “Stroke + Semax”, 7 days after PT; 7,8: Intact rats receiving a 7-day course of Semax; 9,10: “Stroke + Saline”, 21 days after PT; 11,12: “Stroke + Semax”, 14 days after withdrawal of Semax treatment; 13,14: Intact rats, 14 days after withdrawal of Semax treatment; Figure S18: Representative immunoblot image (Rg-film combined with nitrocellulose membrane) showing expression of TFAM in the prefrontal cortex penumbra from rats with photochemically induced thrombosis (PT) (“Stroke + Saline” group), rats treated with Semax by i/n administration 2 h after PT and for the consecutive 6 days (one instillation per day) at a dose of 25 μg/kg (“Stroke + Semax” group), and intact rats treated with a 7-day course of Semax, for 21 days of observation. Top sequence of blots (first gel; one animal corresponds to each blot), from left to right: 1,2: “Control” (rats received saline); 3,4: “Stroke + Saline”, 24 h after PT; 5,6: “Stroke + Semax”, 24 h after PT; 7,8: Intact rats receiving a single injection of Semax; 9,10: “Stroke + Saline”, 3 days after PT; 11,12: “Stroke + Semax”; 3 days after PT; 13,14: Intact rats receiving a 3-day course of Semax. Bottom sequence of blots (second gel; one animal corresponds to each blot), from left to right: 1,2: “Control”; 3,4: “Stroke + Saline”, 7 days after PT; 5,6: “Stroke + Semax”, 7 days after PT; 7,8: Intact rats receiving a 7-day course of Semax; 9,10: “Stroke + Saline”, 21 days after PT; 11,12: “Stroke + Semax”, 14 days after withdrawal of Semax treatment; 13,14: Intact rats, 14 days after withdrawal of Semax treatment; Figure S19: Representative immunoblot image (Rg-film combined with nitrocellulose membrane) showing expression of NDUFV2 in the prefrontal cortex penumbra from rats with photochemically induced thrombosis (PT) (“Stroke + Saline” group), rats treated with Semax by i/n administration 2 h after PT and for the consecutive 6 days (one instillation per day) at a dose of 25 μg/kg (“Stroke + Semax” group), and intact rats treated with a 7-day course of Semax, for 21 days of observation. Top sequence of blots (first gel; one animal corresponds to each blot), from left to right: 1,2: “Control” (rats received saline); 3,4: “Stroke + Saline”, 24 h after PT; 5,6: “Stroke + Semax”, 24 h after PT; 7,8: Intact rats receiving a single injection of Semax; 9,10: “Stroke + Saline”, 3 days after PT; 11,12: “Stroke + Semax”; 3 days after PT; 13,14: Intact rats receiving a 3-day course of Semax. Bottom sequence of blots (second gel; one animal corresponds to each blot), from left to right: 1,2: “Control”; 3,4: “Stroke + Saline”, 7 days after PT; 5,6: “Stroke + Semax”, 7 days after PT; 7,8: Intact rats receiving a 7-day course of Semax; 9,10: “Stroke + Saline”, 21 days after PT; 11,12: “Stroke + Semax”, 14 days after withdrawal of Semax treatment; 13,14: Intact rats, 14 days after withdrawal of Semax treatment; Figure S20: Representative immunoblot image (Rg-film combined with nitrocellulose membrane) showing expression of SDHA in the prefrontal cortex penumbra from rats with photochemically induced thrombosis (PT) (“Stroke + Saline” group), rats treated with Semax by i/n administration 2 h after PT and for the consecutive 6 days (one instillation per day) at a dose of 25 μg/kg (“Stroke + Semax” group), and intact rats treated with a 7-day course of Semax, for 21 days of observation. Top sequence of blots (first gel; one animal corresponds to each blot), from left to right: 1,2: “Control” (rats received saline); 3,4: “Stroke + Saline”, 24 h after PT; 5,6: “Stroke + Semax”, 24 h after PT; 7,8: Intact rats receiving a single injection of Semax; 9,10: “Stroke + Saline”, 3 days after PT; 11,12: “Stroke + Semax”; 3 days after PT; 13,14: Intact rats receiving a 3-day course of Semax. Bottom sequence of blots (second gel; one animal corresponds to each blot), from left to right: 1,2: “Control”; 3,4: “Stroke + Saline”, 7 days after PT; 5,6: “Stroke + Semax”, 7 days after PT; 7,8: Intact rats receiving a 7-day course of Semax; 9,10: “Stroke + Saline”, 21 days after PT; 11,12: “Stroke + Semax”, 14 days after withdrawal of Semax treatment; 13,14: Intact rats, 14 days after withdrawal of Semax treatment; Figure S21: Representative immunoblot image (Rg-film combined with nitrocellulose membrane) showing expression of cyt c1 in the prefrontal cortex penumbra from rats with photochemically induced thrombosis (PT) (“Stroke + Saline” group), rats treated with Semax by i/n administration 2 h after PT and for the consecutive 6 days (one instillation per day) at a dose of 25 μg/kg (“Stroke + Semax” group), and intact rats treated with a 7-day course of Semax, for 21 days of observation. Top sequence of blots (first gel; one animal corresponds to each blot), from left to right: 1,2: “Control” (rats received saline); 3,4: “Stroke + Saline”, 24 h after PT; 5,6: “Stroke + Semax”, 24 h after PT; 7,8: Intact rats receiving a single injection of Semax; 9,10: “Stroke + Saline”, 3 days after PT; 11,12: “Stroke + Semax”; 3 days after PT; 13,14: Intact rats receiving a 3-day course of Semax. Bottom sequence of blots (second gel; one animal corresponds to each blot), from left to right: 1,2: “Control”; 3,4: “Stroke + Saline”, 7 days after PT; 5,6: “Stroke + Semax”, 7 days after PT; 7,8: Intact rats receiving a 7-day course of Semax; 9,10: “Stroke + Saline”, 21 days after PT; 11,12: “Stroke + Semax”, 14 days after withdrawal of Semax treatment; 13,14: Intact rats, 14 days after withdrawal of Semax treatment; Figure S22: Representative immunoblot image (Rg-film combined with nitrocellulose membrane) showing expression of COX2 in the prefrontal cortex penumbra from rats with photochemically induced thrombosis (PT) (“Stroke + Saline” group), rats treated with Semax by i/n administration 2 h after PT and for the consecutive 6 days (one instillation per day) at a dose of 25 μg/kg (“Stroke + Semax” group), and intact rats treated with a 7-day course of Semax, for 21 days of observation. Top sequence of blots (first gel; one animal corresponds to each blot), from left to right: 1,2: “Control” (rats received saline); 3,4: “Stroke + Saline”, 24 h after PT; 5,6: “Stroke + Semax”, 24 h after PT; 7,8: Intact rats receiving a single injection of Semax; 9,10: “Stroke + Saline”, 3 days after PT; 11,12: “Stroke + Semax”; 3 days after PT; 13,14: Intact rats receiving a 3-day course of Semax. Bottom sequence of blots (second gel; one animal corresponds to each blot), from left to right: 1,2: “Control”; 3,4: “Stroke + Saline”, 7 days after PT; 5,6: “Stroke + Semax”, 7 days after PT; 7,8: Intact rats receiving a 7-day course of Semax; 9,10: “Stroke + Saline”, 21 days after PT; 11,12: “Stroke + Semax”, 14 days after withdrawal of Semax treatment; 13,14: Intact rats, 14 days after withdrawal of Semax treatment; Figure S23: Representative immunoblot image (Rg-film combined with nitrocellulose membrane) showing expression of ATP5A in the prefrontal cortex penumbra from rats with photochemically induced thrombosis (PT) (“Stroke + Saline” group), rats treated with Semax by i/n administration 2 h after PT and for the consecutive 6 days (one instillation per day) at a dose of 25 μg/kg (“Stroke + Semax” group), and intact rats treated with a 7-day course of Semax, for 21 days of observation. Top sequence of blots (first gel; one animal corresponds to each blot), from left to right: 1,2: “Control” (rats received saline); 3,4: “Stroke + Saline”, 24 h after PT; 5,6: “Stroke + Semax”, 24 h after PT; 7,8: Intact rats receiving a single injection of Semax; 9,10: “Stroke + Saline”, 3 days after PT; 11,12: “Stroke + Semax”; 3 days after PT; 13,14: Intact rats receiving a 3-day course of Semax. Bottom sequence of blots (second gel; one animal corresponds to each blot), from left to right: 1,2: “Control”; 3,4: “Stroke + Saline”, 7 days after PT; 5,6: “Stroke + Semax”, 7 days after PT; 7,8: Intact rats receiving a 7-day course of Semax; 9,10: “Stroke + Saline”, 21 days after PT; 11,12: “Stroke + Semax”, 14 days after withdrawal of Semax treatment; 13,14: Intact rats, 14 days after withdrawal of Semax treatment; Figure S24: Representative immunoblot image (Rg-film combined with nitrocellulose membrane) showing expression of SUCNR1 in the prefrontal cortex penumbra from rats with photochemically induced thrombosis (PT) (“Stroke + Saline” group), rats treated with Semax by i/n administration 2 h after PT and for the consecutive 6 days (one instillation per day) at a dose of 25 μg/kg (“Stroke + Semax” group), and intact rats treated with a 7-day course of Semax, for 21 days of observation. Top sequence of blots (first gel; one animal corresponds to each blot), from left to right: 1,2: “Control” (rats received saline); 3,4: “Stroke + Saline”, 24 h after PT; 5,6: “Stroke + Semax”, 24 h after PT; 7,8: Intact rats receiving a single injection of Semax; 9,10: “Stroke + Saline”, 3 days after PT; 11,12: “Stroke + Semax”; 3 days after PT; 13,14: Intact rats receiving a 3-day course of Semax. Bottom sequence of blots (second gel; one animal corresponds to each blot), from left to right: 1,2: “Control”; 3,4: “Stroke + Saline”, 7 days after PT; 5,6: “Stroke + Semax”, 7 days after PT; 7,8: Intact rats receiving a 7-day course of Semax; 9,10: “Stroke + Saline”, 21 days after PT; 11,12: “Stroke + Semax”, 14 days after withdrawal of Semax treatment; 13,14: Intact rats, 14 days after withdrawal of Semax treatment; Figure S25: Representative immunoblot image (Rg-film combined with nitrocellulose membrane) showing expression of VEGF in the prefrontal cortex penumbra from rats with photochemically induced thrombosis (PT) (“Stroke + Saline” group), rats treated with Semax by i/n administration 2 h after PT and for the consecutive 6 days (one instillation per day) at a dose of 25 μg/kg (“Stroke + Semax” group), and intact rats treated with a 7-day course of Semax, for 21 days of observation. Top sequence of blots (first gel; one animal corresponds to each blot), from left to right: 1,2: “Control” (rats received saline); 3,4: “Stroke + Saline”, 24 h after PT; 5,6: “Stroke + Semax”, 24 h after PT; 7,8: Intact rats receiving a single injection of Semax; 9,10: “Stroke + Saline”, 3 days after PT; 11,12: “Stroke + Semax”; 3 days after PT; 13,14: Intact rats receiving a 3-day course of Semax. Bottom sequence of blots (second gel; one animal corresponds to each blot), from left to right: 1,2: “Control”; 3,4: “Stroke + Saline”, 7 days after PT; 5,6: “Stroke + Semax”, 7 days after PT; 7,8: Intact rats receiving a 7-day course of Semax; 9,10: “Stroke + Saline”, 21 days after PT; 11,12: “Stroke + Semax”, 14 days after withdrawal of Semax treatment; 13,14: Intact rats, 14 days after withdrawal of Semax treatment; Figure S26: Representative immunoblot image (Rg-film combined with nitrocellulose membrane) showing expression of VE-cadherin in the prefrontal cortex penumbra from rats with photochemically induced thrombosis (PT) (“Stroke + Saline” group), rats treated with Semax by i/n administration 2 h after PT and for the consecutive 6 days (one instillation per day) at a dose of 25 μg/kg (“Stroke + Semax” group), and intact rats treated with a 7-day course of Semax, for 21 days of observation. Top sequence of blots (first gel; one animal corresponds to each blot), from left to right: 1,2: “Control” (rats received saline); 3,4: “Stroke + Saline”, 24 h after PT; 5,6: “Stroke + Semax”, 24 h after PT; 7,8: Intact rats receiving a single injection of Semax; 9,10: “Stroke + Saline”, 3 days after PT; 11,12: “Stroke + Semax”; 3 days after PT; 13,14: Intact rats receiving a 3-day course of Semax. Bottom sequence of blots (second gel; one animal corresponds to each blot), from left to right: 1,2: “Control”; 3,4: “Stroke + Saline”, 7 days after PT; 5,6: “Stroke + Semax”, 7 days after PT; 7,8: Intact rats receiving a 7-day course of Semax; 9,10: “Stroke + Saline”, 21 days after PT; 11,12: “Stroke + Semax”, 14 days after withdrawal of Semax treatment; 13,14: Intact rats, 14 days after withdrawal of Semax treatment; Figure S27: Representative immunoblot image (Rg-film combined with nitrocellulose membrane) showing expression of Synaptophysin in the prefrontal cortex penumbra from rats with photochemically induced thrombosis (PT) (“Stroke + Saline” group), rats treated with Semax by i/n administration 2 h after PT and for the consecutive 6 days (one instillation per day) at a dose of 25 μg/kg (“Stroke + Semax” group), and intact rats treated with a 7-day course of Semax, for 21 days of observation. Top sequence of blots (first gel; one animal corresponds to each blot), from left to right: 1,2: “Control” (rats received saline); 3,4: “Stroke + Saline”, 24 h after PT; 5,6: “Stroke + Semax”, 24 h after PT; 7,8: Intact rats receiving a single injection of Semax; 9,10: “Stroke + Saline”, 3 days after PT; 11,12: “Stroke + Semax”; 3 days after PT; 13,14: Intact rats receiving a 3-day course of Semax. Bottom sequence of blots (second gel; one animal corresponds to each blot), from left to right: 1,2: “Control”; 3,4: “Stroke + Saline”, 7 days after PT; 5,6: “Stroke + Semax”, 7 days after PT; 7,8: Intact rats receiving a 7-day course of Semax; 9,10: “Stroke + Saline”, 21 days after PT; 11,12: “Stroke + Semax”, 14 days after withdrawal of the Semax treatment; 13,14: Intact rats, 14 days after withdrawal of the Semax treatment; Figure S28: Representative fluorescence micrographs of PGC-1α (red) and NeuN (green) staining of the prefrontal cortex histological sections from rats with photochemically induced thrombosis (PT) that received an i/p saline injection 2 h after PT and for the consecutive 6 days (one injection per day) (“Stroke + Saline” experimental group). The penumbra area is shown in the larger rectangle, and the ischemic zone is shown in the smaller rectangle. Designations: nN: Nucleus of neuron, cN: Cytoplasm of neuron. Scale bar = 50 µm; Figure S29: Representative fluorescence micrographs of PGC-1α (red) and NeuN (green) staining of the prefrontal cortex histological sections from rats with photochemically induced thrombosis (PT) treated with Mexidol by an i/p injection 2 h after PT and for the consecutive 6 days (one injection per day) at a dose of 100 mg/kg (“Stroke + Mexidol” experimental group). The penumbra area is shown in the larger rectangle, and the ischemic zone is shown in the smaller rectangle. Designations: nN: Nucleus of neuron, cN: Cytoplasm of neuron. Scale bar = 50 µm; Figure S30: Representative fluorescence micrographs of PGC-1α (red) and NeuN (green) staining of the prefrontal cortex histological sections from rats with photochemically induced thrombosis (PT) treated with Semax by i/n administration 2 h after PT and for the consecutive 6 days (one instillation per day) at a dose of 25 μg/kg (“Stroke + Semax” experimental group). The penumbra area is shown in the larger rectangle, and the ischemic zone is shown in the smaller rectangle. Designations: nN: Nucleus of neuron, cN: Cytoplasm of neuron. Scale bar = 50 µm; Figure S31: (A) Representative photomicrographs of the prefrontal cortex sections immunostained with antibodies against PGC-1α (red) and NeuN (green). The nuclei of neurons and glial cells were stained with DAPI (blue). a,b,d,e: Original images; c,f: Merged images. (B) Algorithm for the quantitative assessment of the PGC-1α immunoreactivity in the nucleus and perinuclear cytoplasm of neurons. Screenshots obtained during the programmed quantitative analysis (VideoTesT-Morphology 5.2 (LLC «VideoTesT», Russia) software) of fluorescent microphotographs are demonstrated. a,e: Merged images (yellow) of representative fluorescence micrographs of PGC-1α (red) and NeuN (green) staining; b,f: In merged images, the nuclear area (b) and perinuclear cytoplasm (f) were outlined (see dashed lines); c,g: NeuN images are made transparent; d,h: PGC-1α images are switched to a monochrome mode (gray) for the final densitometry of the nuclear region (d) and ring-shaped figure of the perinuclear cytoplasm (h).

## Abbreviations

ACTH	adrenocorticotropic hormone
AICAR	5-aminoimidazole-4-carboxamide ribonucleotide
AMP	adenosine monophosphate
AMPAR	α-amino-3-hydroxy-5-methyl-4-isoxazolepropionic acid receptor
AMPK	AMP-activated protein kinase
ATP5A	ATP sythase (F1-ATPase) alpha subunit
BBB	blood–brain barrier
Bcl-2	B-cell lymphoma 2 anti-apoptotic protein
BDNF	brain-derived neurotrophic factor
CaMK	Ca^2+^/calmodulin-dependent protein kinase
cAMP	cyclic adenosine monophosphate
CAT	catalase
CNS	central nervous system
COX2	cytochrome c oxidase subunit 2
CRE	cyclic AMP response element
CREB	cyclic AMP response element binding protein
cyt c1	cytochrome c1
DAMP	damage-associated molecular patterns
ERR	estrogen-related receptor
GABAR	gamma-aminobutyric acid receptor
GHs	glucocorticoid hormones
GPR 91	G-protein-coupled receptor
GPXs	glutathione peroxidases
HIF-1α	hypoxia-inducible factor 1-alpha
IL-10	interleukin-10
IR	immunoreactivity
MAPK	mitogen-activated protein kinase
MCR	melanocortin receptor
Mfn	mitofusin
MYC	cellular homolog of the oncogene (v-myc) of avian myelocytomatosis virus strain
NAD	nicotinamide adenine dinucleotide
NDUFV2	NADH dehydrogenase [ubiquinone] flavoprotein 2, mitochondrial
NMDAR	N-methyl-D-aspartate receptor
NRF	nuclear respiratory factor
PBS	phosphate buffered saline
PC	prefrontal cortex
PGC-1α	peroxisome proliferator-activated receptor gamma coactivator 1 alpha
PI	photothrombotic ischemia
PI3K	phosphoinositide 3-kinases
PPAR	peroxisome proliferator-activated receptor
SDHA	succinate dehydrogenase, subunit A
SIRT1	silent information regulator two proteins (sirtuins)
SOD	superoxide dismutase
SP1	specificity protein 1
SUCNR1	succinate receptor 1
SYP	synaptophysin
TFAM	mitochondrial transcription factor A
TGF-β	transforming growth factor beta
THs	thyroid hormones
TRs	thyroid hormone receptors
VEC	vascular endothelial cadherin
VEGF	vascular endothelial growth factor
