# Supplementary material for: Protective Effects of PGC-1α Activators on Ischemic Stroke in a Rat Model of Photochemically Induced Thrombosis
Source: Brain Sci. 2021 Mar 4;11(3):325. doi: 10.3390/brainsci11030325 (PMC8002020; doi:10.3390/brainsci11030325)
Supplement: Supplementary file 1 [file brainsci-11-00325-s001.pdf]

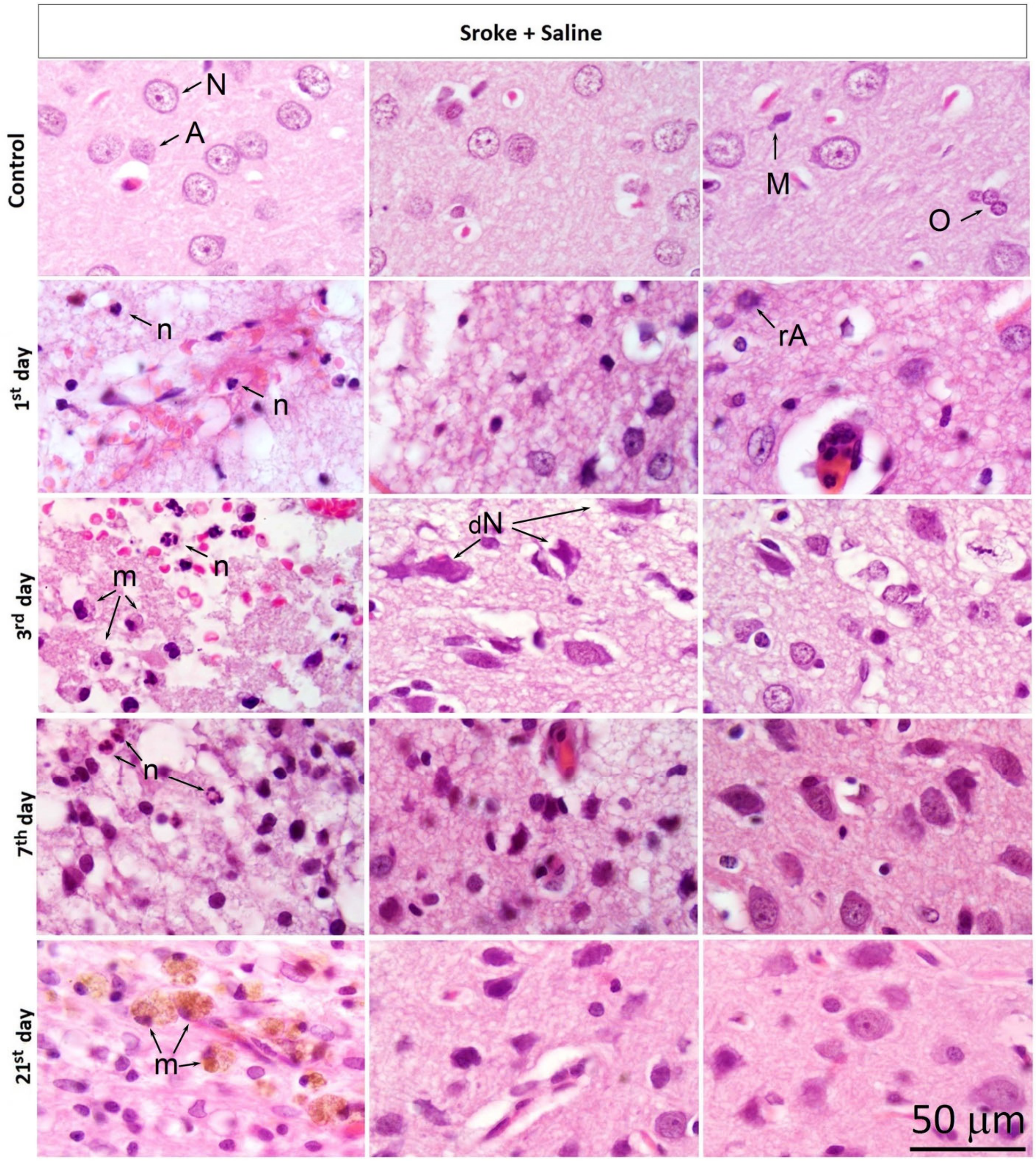

Supplementary Figure 1. Photomicrographs from sections of the prefrontal cortex of rats with photochemically induced thrombosis (PT) that received i/p saline injection 2 h after PT and for the consecutive 6 days (1 injection per day) ("Stroke+Saline" experimental group). Hematoxylin and eosin staining. Lens x100. Sections show the distribution of resident cells of the nervous system and leukocytes recruited from the blood in the structure of the ischemic penumbra. Designations: N – neuron, A – astrocyte, M – microglia, O – oligodendrocyte, dN – degenerating neuron, rA – reactive astrocyte, n – neutrophil, m – monocyte/macrophage.

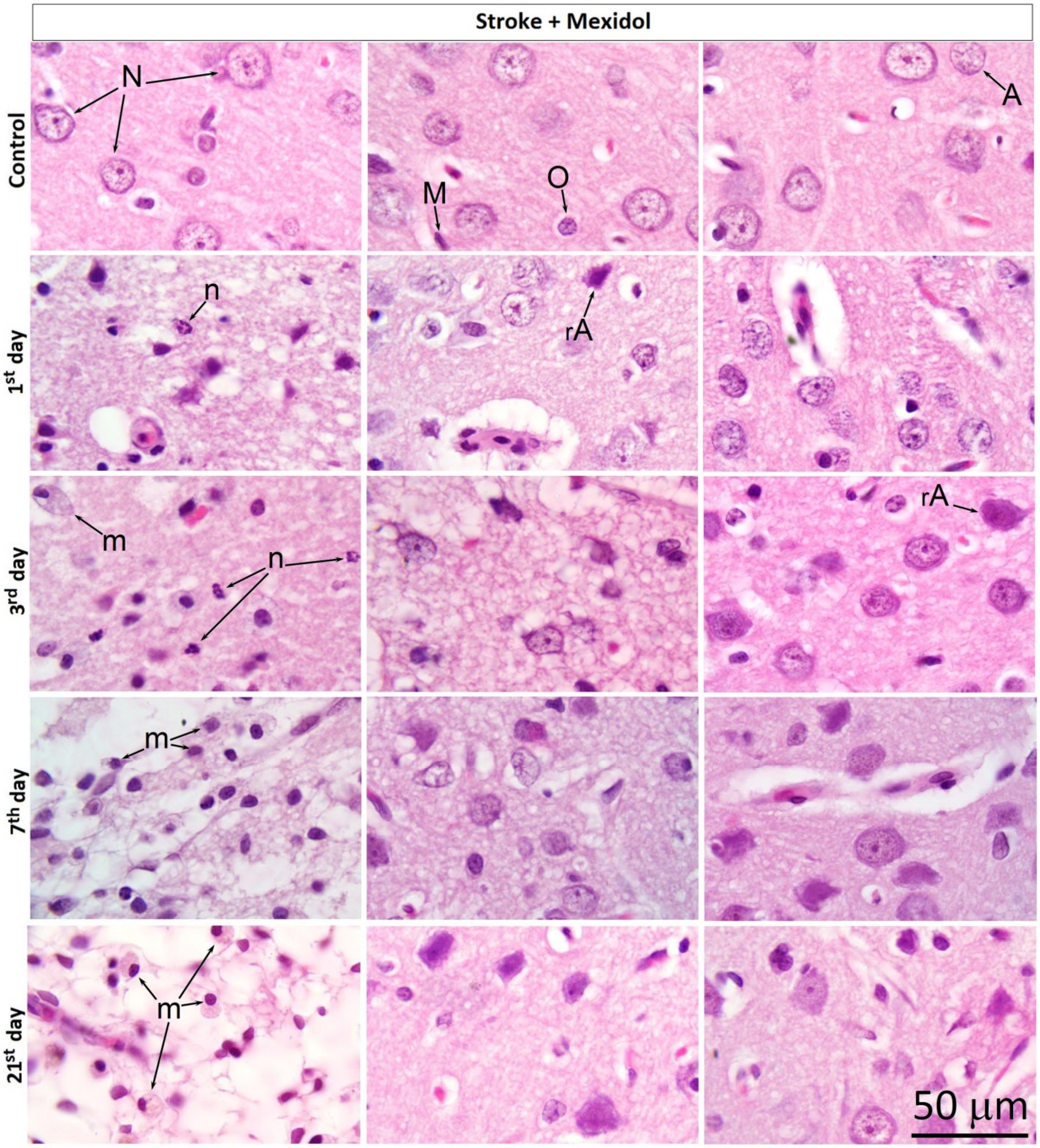

Supplementary Figure 2. Photomicrographs from sections of the prefrontal cortex of rats with photochemically induced thrombosis (PT) that treated with Mexidol by i/p injection 2 h after PT and for the consecutive 6 days (1 injection per day) at a dose 100 mg/kg ("Stroke+Mexidol" experimental group). Hematoxylin and eosin staining. Lens x100. Sections show the distribution of resident cells of the nervous system and leukocytes recruited from the blood in the structure of the ischemic penumbra. Designations: N – neuron, A – astrocyte, M – microglia, O – oligodendrocyte, dN – degenerating neuron, rA – reactive astrocyte, n – neutrophil, m – monocyte/macrophage.

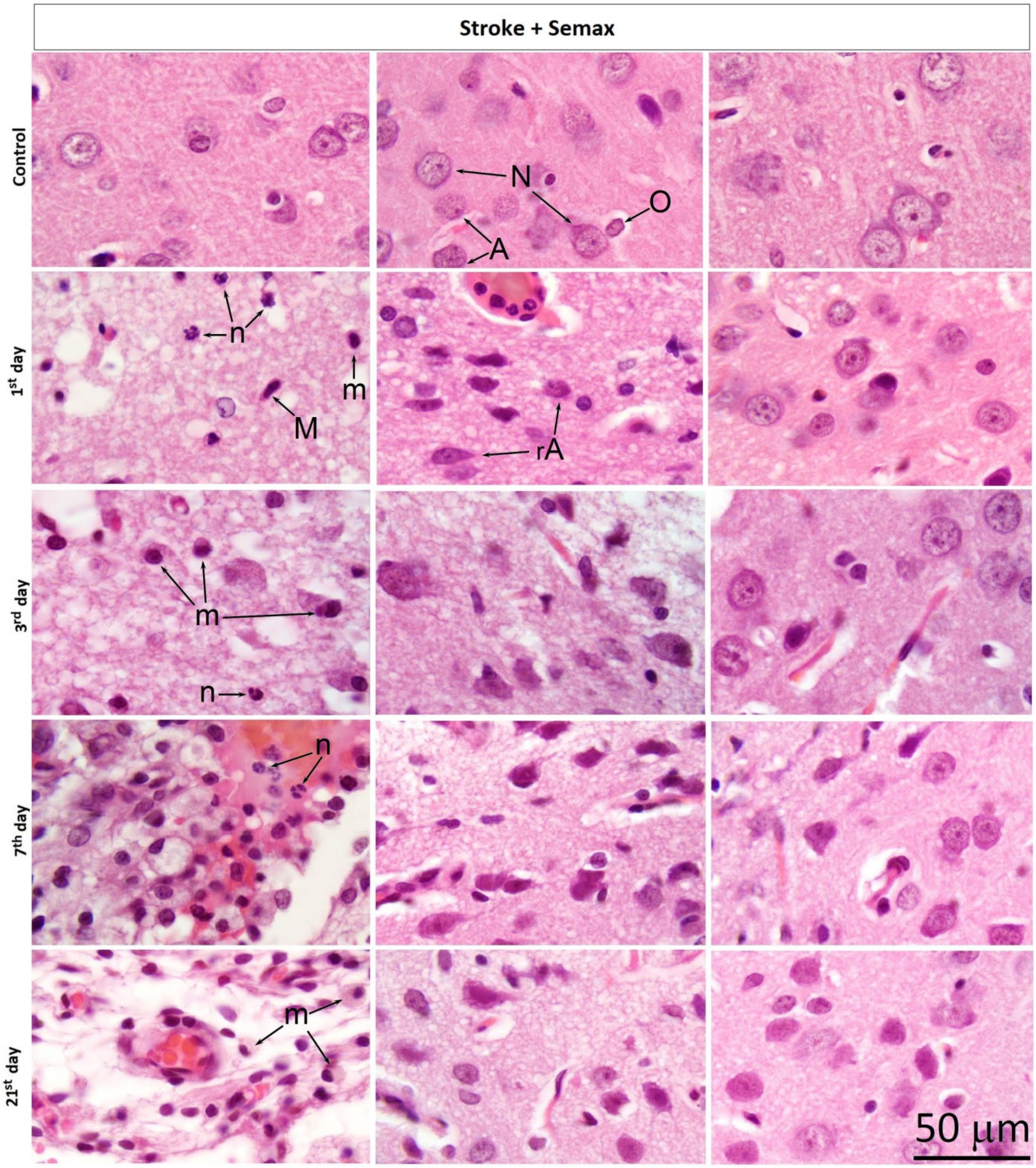

Supplementary Figure 3. Photomicrographs from sections of the prefrontal cortex of rats with photochemically induced thrombosis (PT) that treated with Semax by i/n administration 2 h after PT and for the consecutive 6 days (1 instillation per day) at a dose 25 µg/kg ("Stroke+Semax" experimental group). Hematoxylin and eosin staining. Lens x100. Sections show the distribution of resident cells of the nervous system and leukocytes recruited from the blood in the structure of the ischemic penumbra. Designations: N – neuron, A – astrocyte, M – microglia, O – oligodendrocyte, dN – degenerating neuron, rA – reactive astrocyte, n – neutrophil, m – monocyte/macrophage.

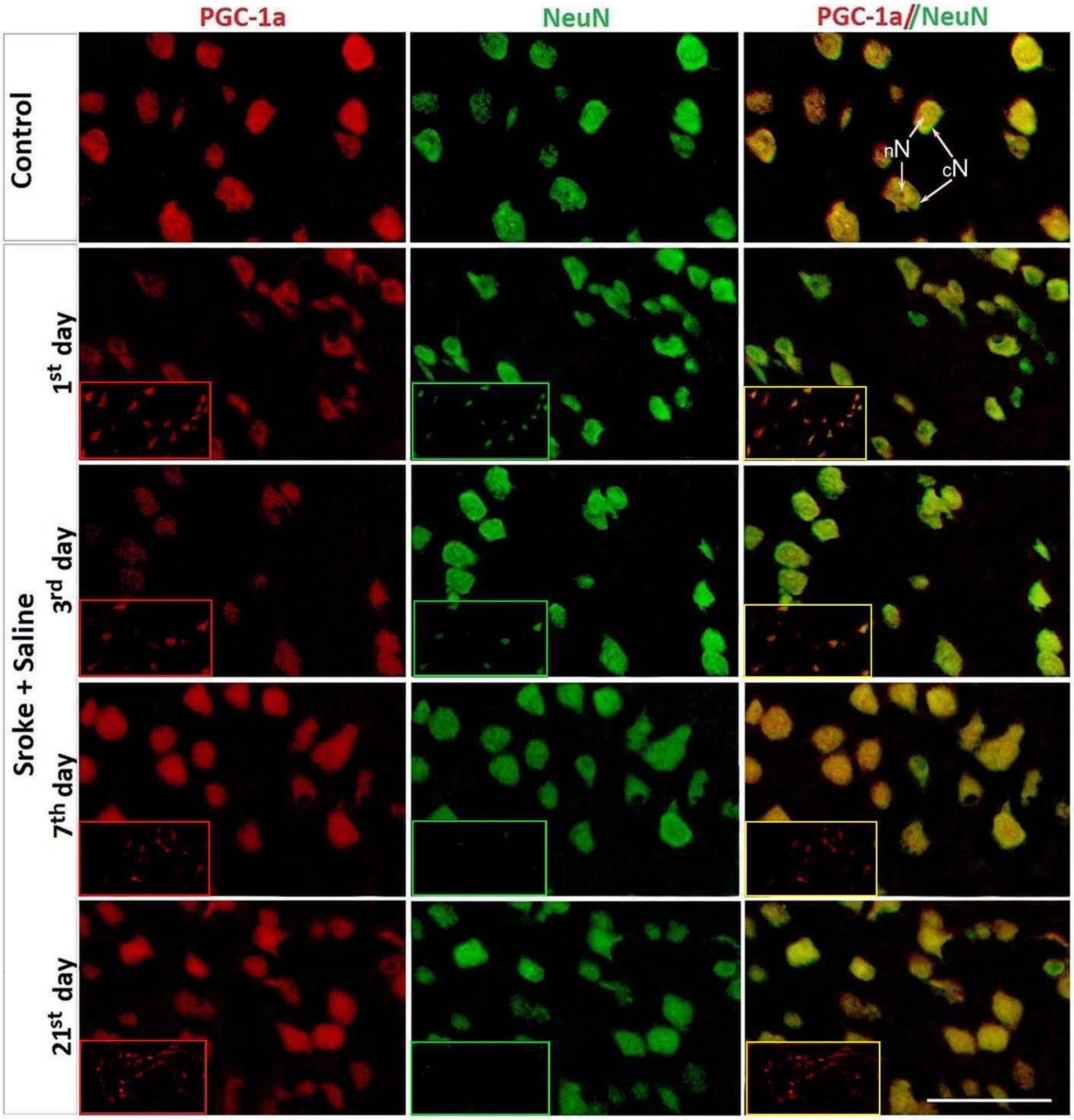

Supplementary Figure 4. Representative fluorescence micrographs of PGC-1α (red) and NeuN (green) staining of the prefrontal cortex histological sections from rat with photochemically induced thrombosis (PT) that received i/p saline injection 2 h after PT and for the consecutive 6 days (1 injection per day) ("Stroke+Saline" experimental group). The penumbra area is shown in the larger rectangle, and the ischemic zone is shown in the smaller rectangle. Designations: nN – nucleus of neuron, cN – cytoplasm of neuron. Scale bar = 50 μm.

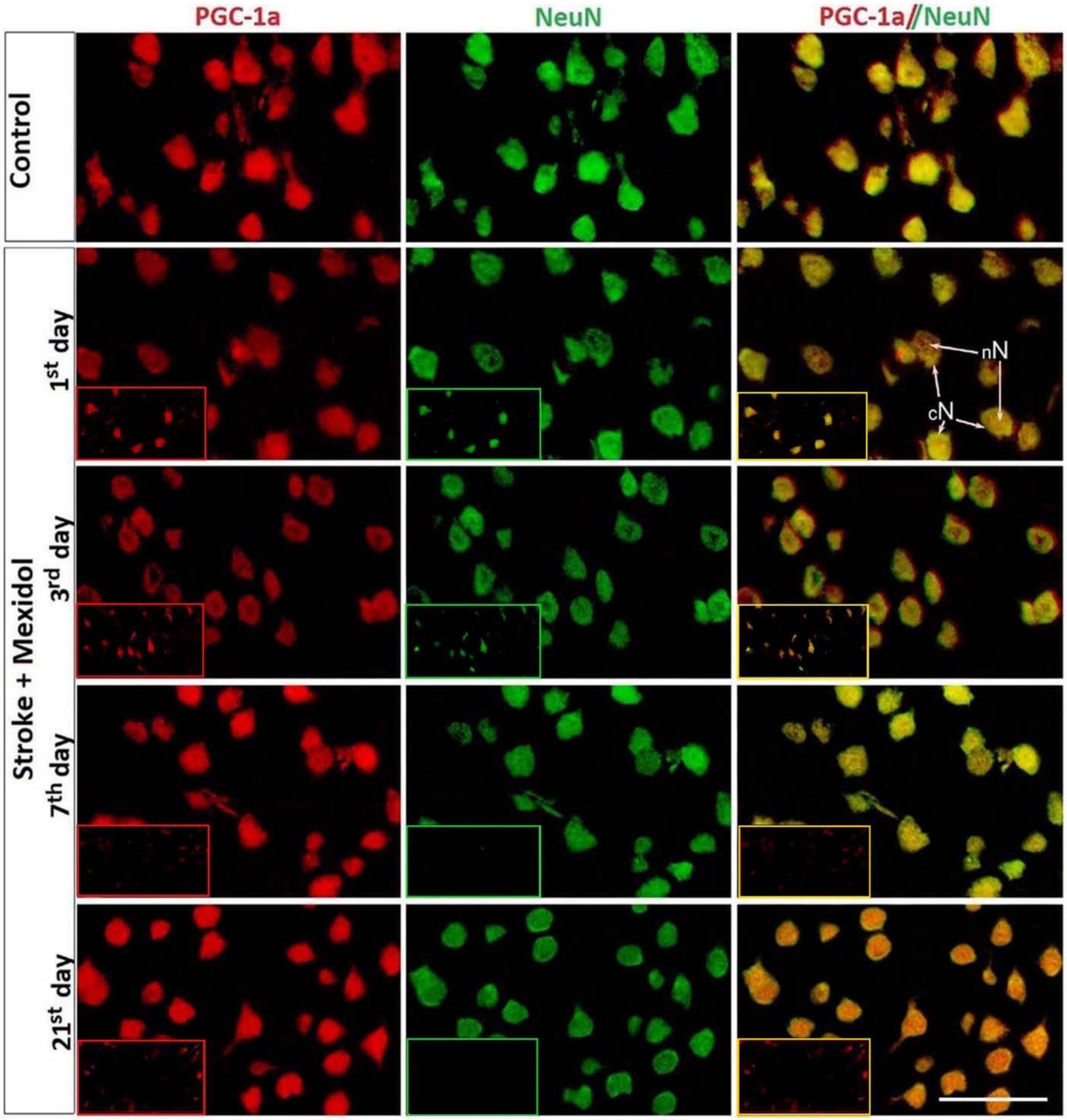

Supplementary Figure 5. Representative fluorescence micrographs of PGC-1α (red) and NeuN (green) staining of the prefrontal cortex histological sections from rat with photochemically induced thrombosis (PT) that treated with Mexidol by i/p injection 2 h after PT and for the consecutive 6 days (1 injection per day) at a dose 100 mg/kg ("Stroke+Mexidol" experimental group). The penumbra area is shown in the larger rectangle, and the ischemic zone is shown in the smaller rectangle. Designations: nN – nucleus of neuron, cN – cytoplasm of neuron. Scale bar = 50 μm.

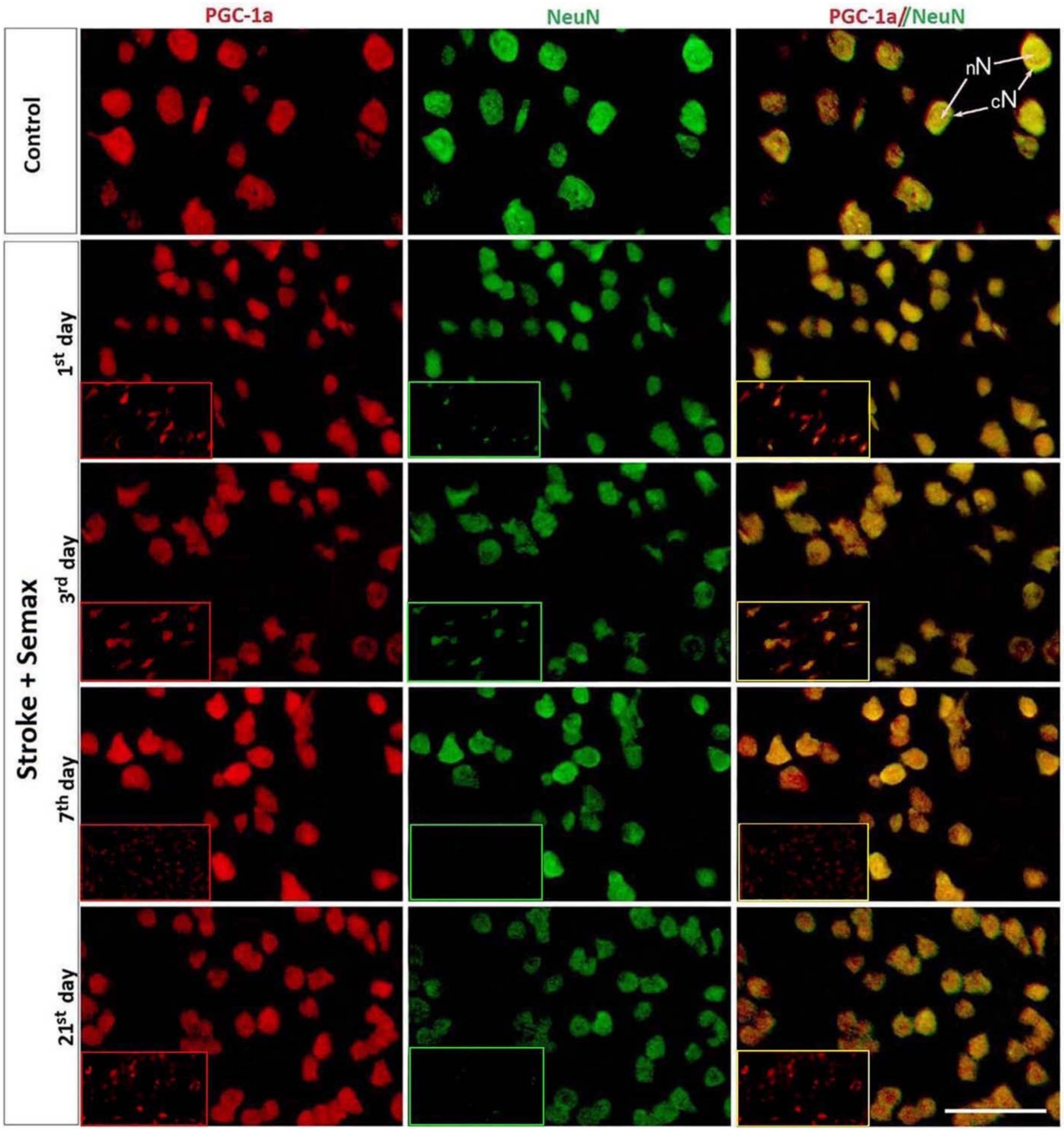

Supplementary Figure 6. Representative fluorescence micrographs of PGC-1α (red) and NeuN (green) staining of the prefrontal cortex histological sections from rat with photochemically induced thrombosis (PT) that treated with Semax by i/n administration 2 h after PT and for the consecutive 6 days (1 instillation per day) at a dose 25 μg/kg) ("Stroke+Semax" experimental group). The penumbra area is shown in the larger rectangle, and the ischemic zone is shown in the smaller rectangle. Designations: nN – nucleus of neuron, cN – cytoplasm of neuron. Scale bar = 50 μm.

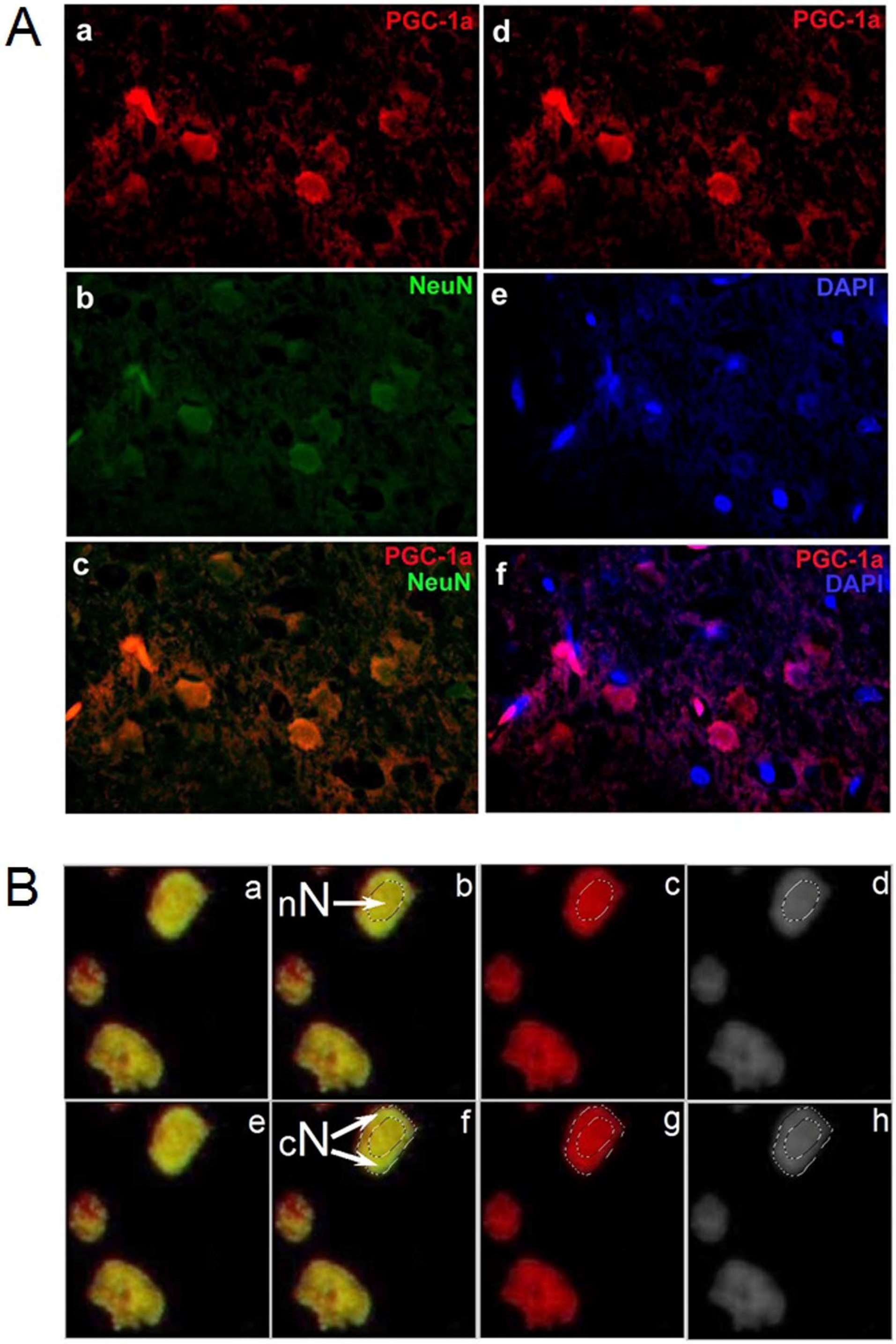

Supplementary Figure 7. (A) Representative photomicrographs of the prefrontal cortex sections immunostained with antibodies against PGC-1α (red) and NeuN (green). The nuclei of neurons and glial cells were stained with DAPI (blue). a, b, d, e - original images; c, f - merged images. (B) Algorithm for quantitative assessment of the PGC-1α immunoreactivity in the nucleus and perinuclear cytoplasm of neurons. Screenshots obtained during the programmed quantitative analysis (VideoTesT-Morphology 5.2 (LLC «VideoTesT», Russia) software) of fluorescent microphotographs are demonstrated. a,e – Merged images (yellow) of representative fluorescence micrographs of PGC-1α (red) and NeuN (green) staining. b,f – In merged images, the nuclear area (b) and perinuclear cytoplasm (f) was outlined (see dashed lines). c,g - NeuN images is made transparent. d,h - PGC-1α images is switched to monochrome mode (gray) for the final densitometry of the nuclear region (d) and ring-shaped figure of perinuclear cytoplasm (h).

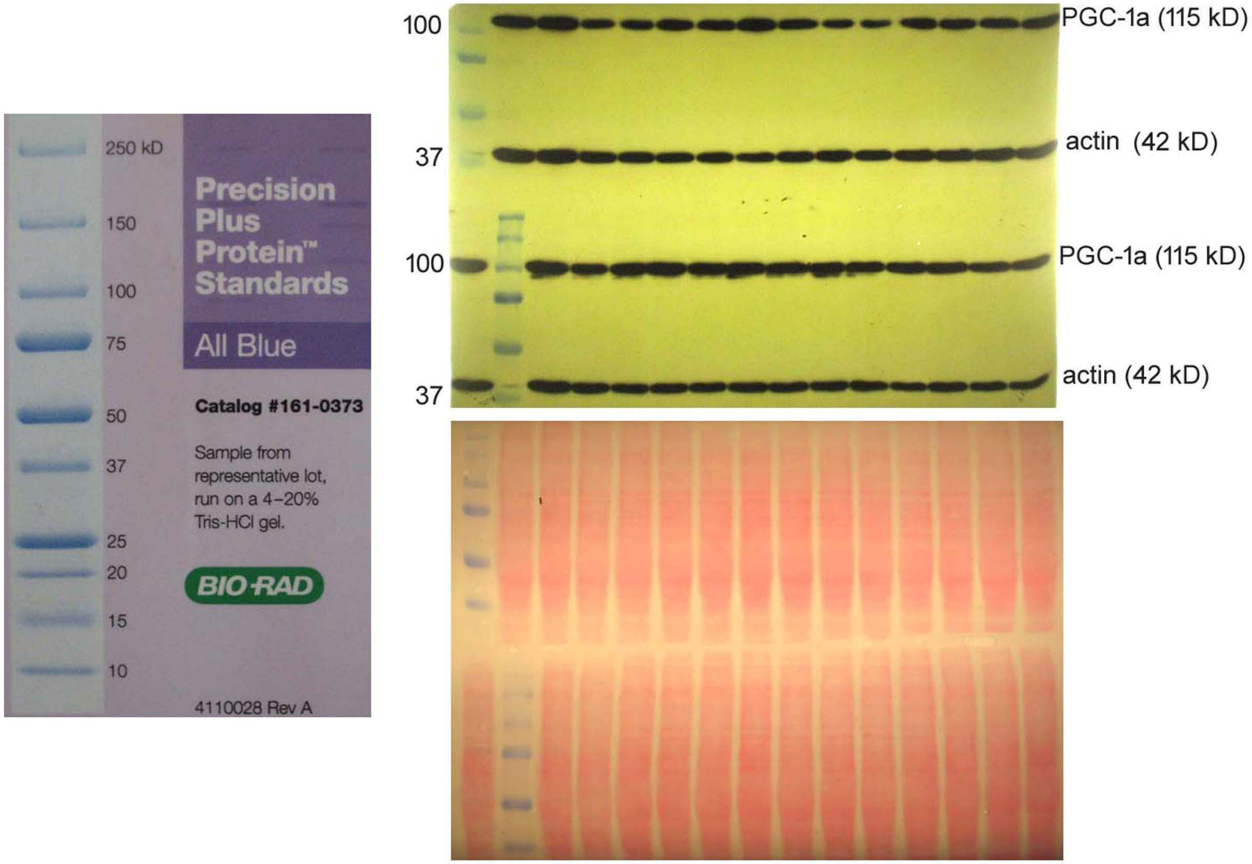

Supplementary Figure 8. Representative immunoblot image (Rg-film combined with nitrocellulose membrane) showing expression of PGC-1α in the prefrontal cortex penumbra from rats with photochemically induced thrombosis (PT) ("Stroke + Saline" group), rats treated with Mexidol by i/p injection 2 h after PT and for the consecutive 6 days (1 injection per day) at a dose 100 mg/kg ("Stroke + Mexidol" group), and intact rats treated with a 7-day course of Mexidol, for 21 days of observation. **Top sequence of blots** (first gel; one animal corresponds to each blot), from left to right: **1, 2** - "Control" (rats received saline according to the same regimen as in the "Stroke+Mexidol" group); **3, 4** - "Stroke+Saline", 24 h after PT; **5, 6** - "Stroke+Mexidol", 24 h after PT; **7, 8** - intact rats receiving a single injection of Mexidol; **9, 10** - "Stroke+Saline", 3 days after PT; **11, 12** - "Stroke+Mexidol"; 3 days after PT; **13, 14** - intact rats receiving a 3-days course of Mexidol; **Bottom sequence of blots** (second gel one; animal corresponds to each blot), from left to right: **1, 2** - "Control"; **3, 4** - "Stroke+Saline", 7 days after PT; **5, 6** - "Stroke+Mexidol", 7 days after PT; **7, 8** - intact rats receiving a 7-days course of Mexidol; **9, 10** - "Stroke+Saline", 21 days after PT; **11, 12** - "Stroke+Mexidol", 14 days after withdrawal of Mexidol treatment; **13, 14** - intact rats, 14 days after withdrawal of Mexidol treatment.

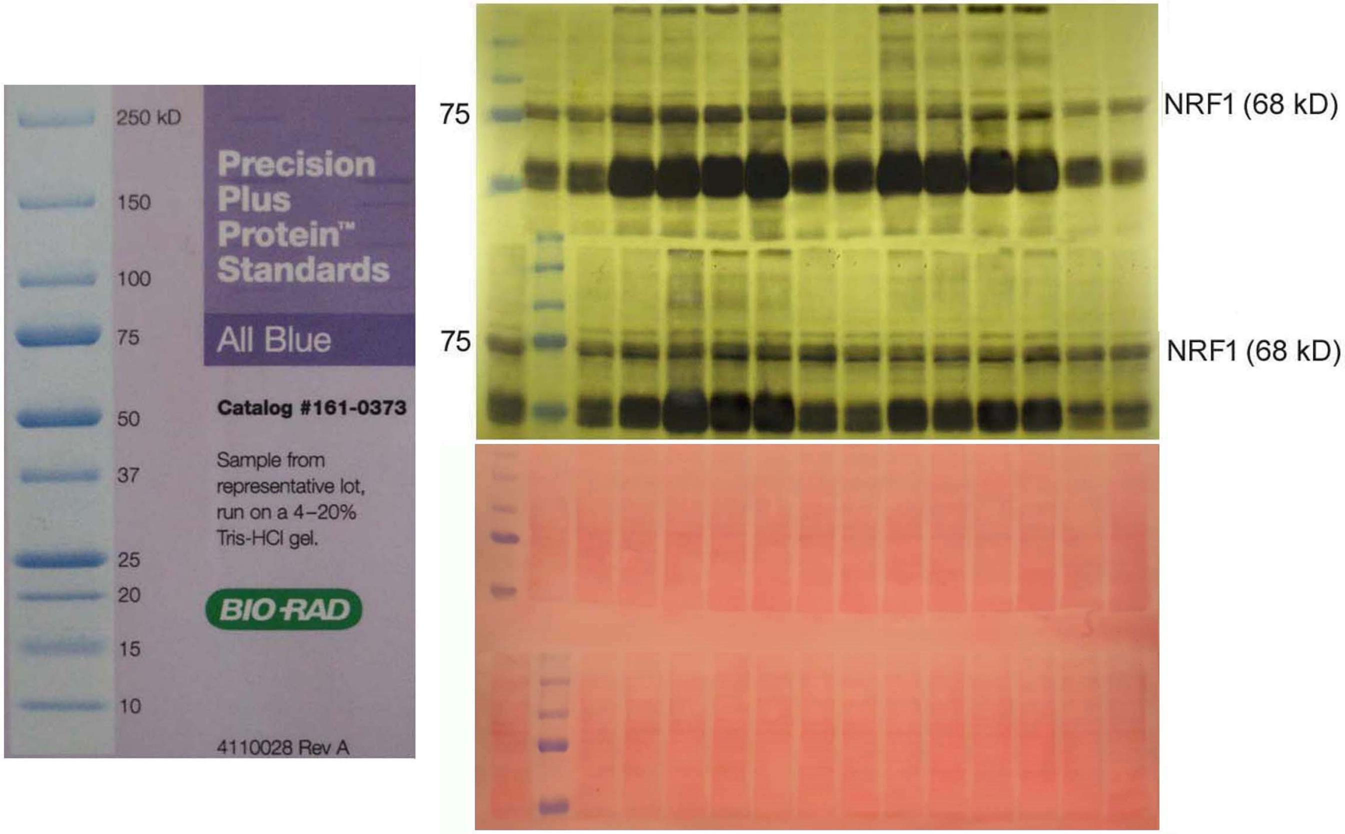

Supplementary Figure 9. Representative immunoblot image (Rg-film combined with nitrocellulose membrane) showing expression of NRF1 in the prefrontal cortex penumbra from rats with photochemically induced thrombosis (PT) ("Stroke + Saline" group), rats treated with Mexidol by i/p injection 2 h after PT and for the consecutive 6 days (1 injection per day) at a dose 100 mg/kg ("Stroke + Mexidol" group), and intact rats treated with a 7-day course of Mexidol, for 21 days of observation. **Top sequence of blots** (first gel; one animal corresponds to each blot), from left to right: **1, 2** - "Control" (rats received saline according to the same regimen as in the "Stroke+Mexidol" group); **3, 4** - "Stroke+Saline", 24 h after PT; **5, 6** - "Stroke+Mexidol", 24 h after PT; **7, 8** - intact rats receiving a single injection of Mexidol; **9, 10** - "Stroke+Saline", 3 days after PT; **11, 12** - "Stroke+Mexidol"; 3 days after PT; **13, 14** - intact rats receiving a 3-days course of Mexidol; **Bottom sequence of blots** (second gel one; animal corresponds to each blot), from left to right: **1, 2** - "Control"; **3, 4** - "Stroke+Saline", 7 days after PT; **5, 6** - "Stroke+Mexidol", 7 days after PT; **7, 8** - intact rats receiving a 7-days course of Mexidol; **9, 10** - "Stroke+Saline", 21 days after PT; **11, 12** - "Stroke+Mexidol", 14 days after withdrawal of Mexidol treatment; **13, 14** - intact rats, 14 days after withdrawal of Mexidol treatment.

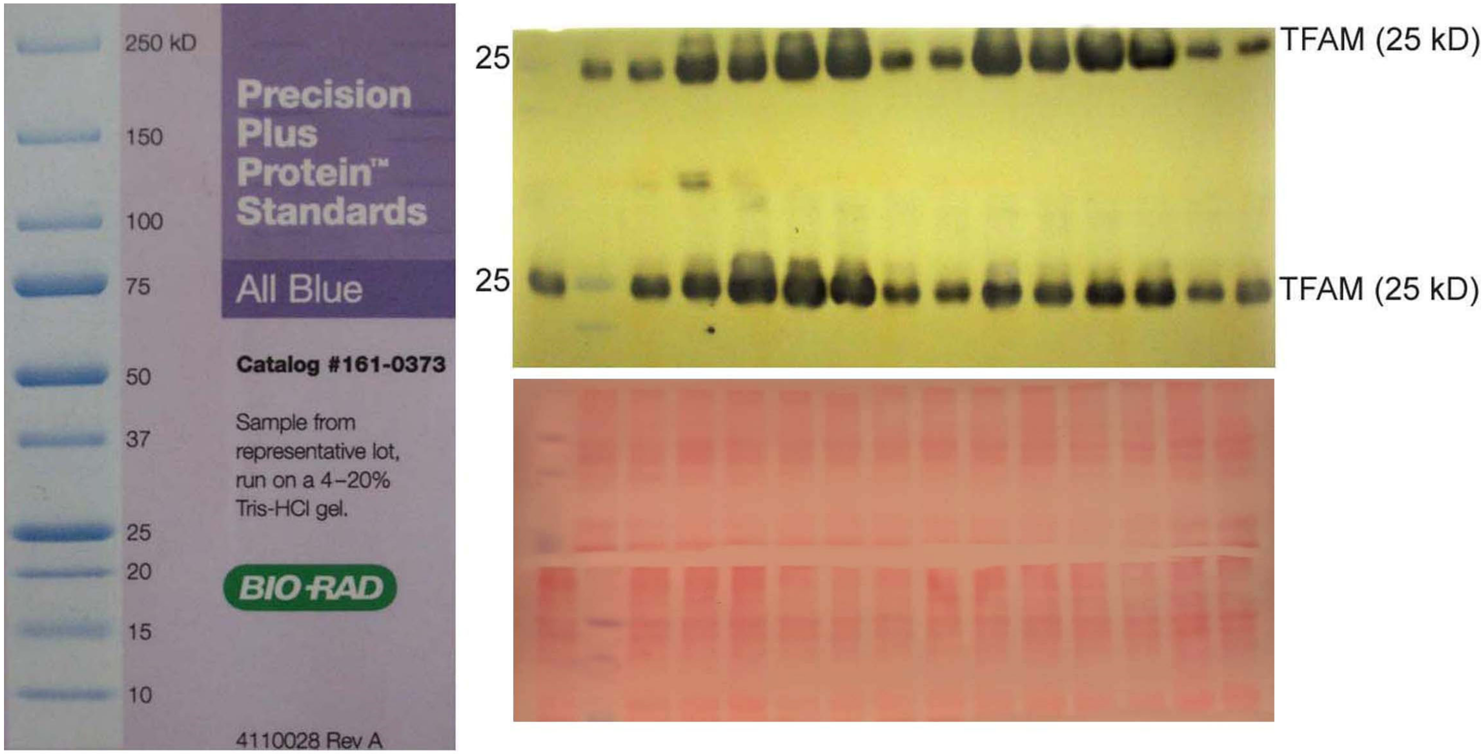

Supplementary Figure 10. Representative immunoblot image (Rg-film combined with nitrocellulose membrane) showing expression of TFAM in the prefrontal cortex penumbra from rats with photochemically induced thrombosis (PT) ("Stroke + Saline" group), rats treated with Mexidol by i/p injection 2 h after PT and for the consecutive 6 days (1 injection per day) at a dose 100 mg/kg ("Stroke + Mexidol" group), and intact rats treated with a 7-day course of Mexidol, for 21 days of observation. **Top sequence of blots** (first gel; one animal corresponds to each blot), from left to right: **1, 2** - "Control" (rats received saline according to the same regimen as in the "Stroke+Mexidol" group); **3, 4** - "Stroke+Saline", 24 h after PT; **5, 6** - "Stroke+Mexidol", 24 h after PT; **7, 8** - intact rats receiving a single injection of Mexidol; **9, 10** - "Stroke+Saline", 3 days after PT; **11, 12** - "Stroke+Mexidol"; 3 days after PT; **13, 14** - intact rats receiving a 3-days course of Mexidol; **Bottom sequence of blots** (second gel one; animal corresponds to each blot), from left to right: **1, 2** - "Control"; **3, 4** - "Stroke+Saline", 7 days after PT; **5, 6** - "Stroke+Mexidol", 7 days after PT; **7, 8** - intact rats receiving a 7-days course of Mexidol; **9, 10** - "Stroke+Saline", 21 days after PT; **11, 12** - "Stroke+Mexidol", 14 days after withdrawal of Mexidol treatment; **13, 14** - intact rats, 14 days after withdrawal of Mexidol treatment.

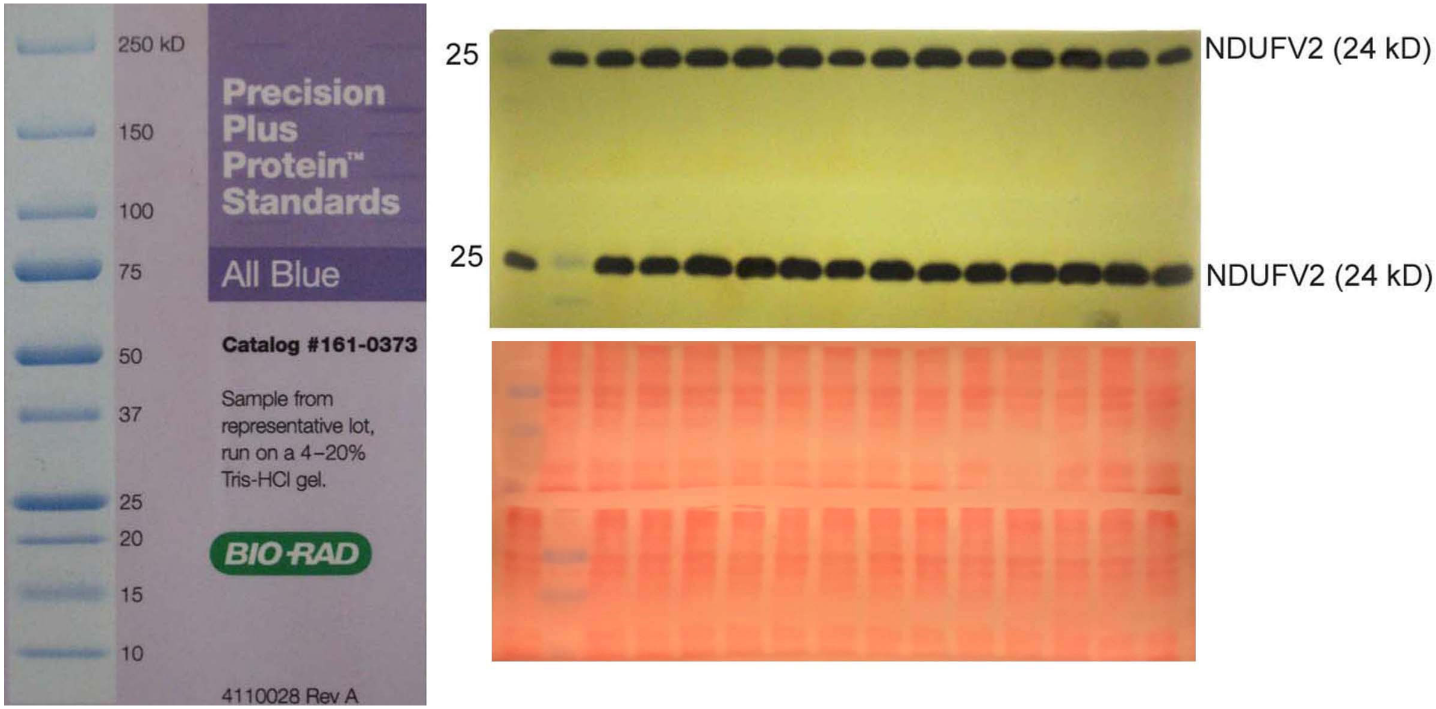

Supplementary Figure 11. Representative immunoblot image (Rg-film combined with nitrocellulose membrane) showing expression of NDUFV2 in the prefrontal cortex penumbra from rats with photochemically induced thrombosis (PT) ("Stroke + Saline" group), rats treated with Mexidol by i/p injection 2 h after PT and for the consecutive 6 days (1 injection per day) at a dose 100 mg/kg ("Stroke + Mexidol" group), and intact rats treated with a 7-day course of Mexidol, for 21 days of observation. **Top sequence of blots** (first gel; one animal corresponds to each blot), from left to right: **1, 2** - "Control" (rats received saline according to the same regimen as in the "Stroke+Mexidol" group); **3, 4** - "Stroke+Saline", 24 h after PT; **5, 6** - "Stroke+Mexidol", 24 h after PT; **7, 8** - intact rats receiving a single injection of Mexidol; **9, 10** - "Stroke+Saline", 3 days after PT; **11, 12** - "Stroke+Mexidol"; 3 days after PT; **13, 14** - intact rats receiving a 3-days course of Mexidol; **Bottom sequence of blots** (second gel one; animal corresponds to each blot), from left to right: **1, 2** - "Control"; **3, 4** - "Stroke+Saline", 7 days after PT; **5, 6** - "Stroke+Mexidol", 7 days after PT; **7, 8** - intact rats receiving a 7-days course of Mexidol; **9, 10** - "Stroke+Saline", 21 days after PT; **11, 12** - "Stroke+Mexidol", 14 days after withdrawal of Mexidol treatment; **13, 14** - intact rats, 14 days after withdrawal of Mexidol treatment.

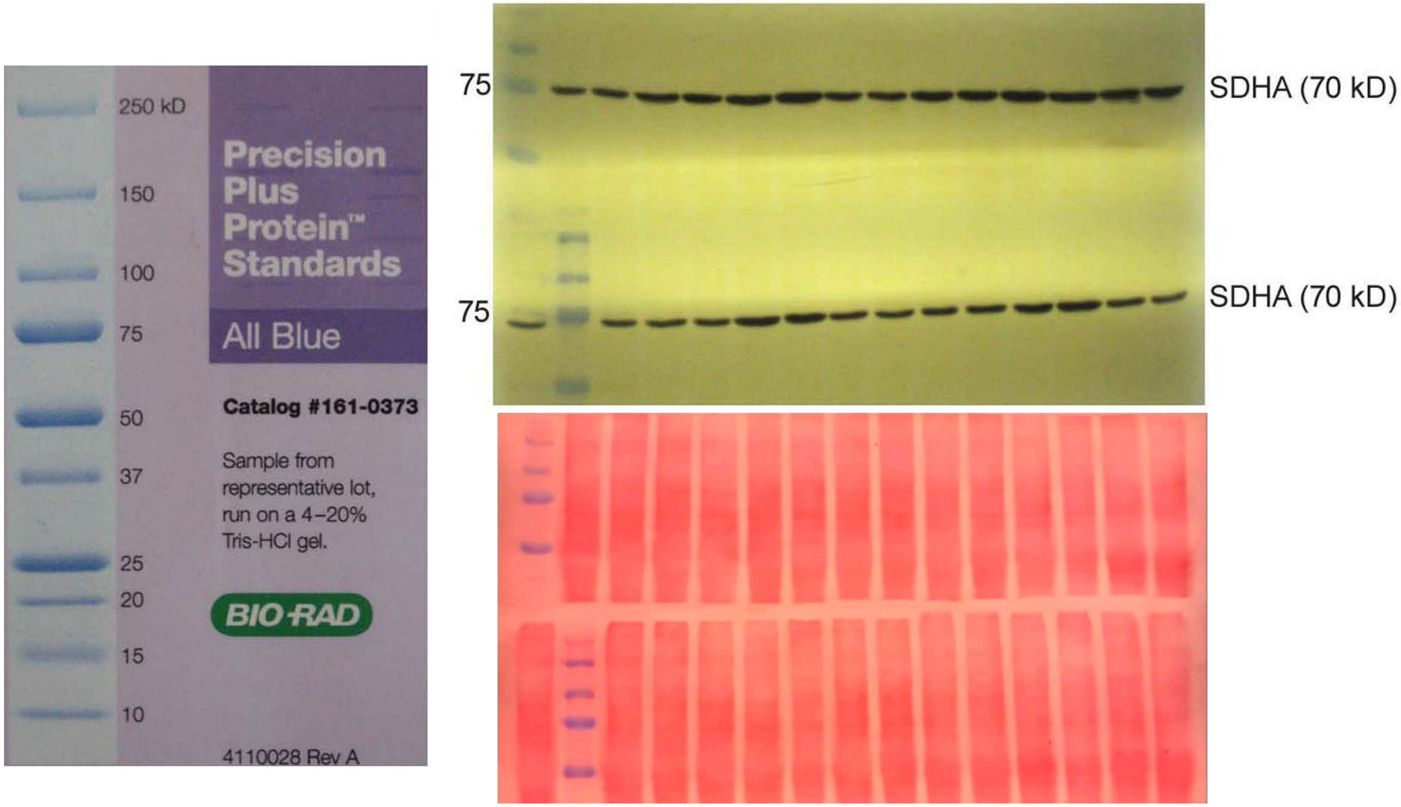

Supplementary Figure 12. Representative immunoblot image (Rg-film combined with nitrocellulose membrane) showing expression of SDHA in the prefrontal cortex penumbra from rats with photochemically induced thrombosis (PT) (“Stroke + Saline” group), rats treated with Mexidol by i/p injection 2 h after PT and for the consecutive 6 days (1 injection per day) at a dose 100 mg/kg (“Stroke + Mexidol” group), and intact rats treated with a 7-day course of Mexidol, for 21 days of observation. **Top sequence of blots** (first gel; one animal corresponds to each blot), from left to right: **1, 2** - “Control” (rats received saline according to the same regimen as in the “Stroke+Mexidol” group); **3, 4** - “Stroke+Saline”, 24 h after PT; **5, 6** - “Stroke+Mexidol”, 24 h after PT; **7, 8** - intact rats receiving a single injection of Mexidol; **9, 10** - “Stroke+Saline”, 3 days after PT; **11, 12** - “Stroke+Mexidol”; 3 days after PT; **13, 14** - intact rats receiving a 3-days course of Mexidol; **Bottom sequence of blots** (second gel one; animal corresponds to each blot), from left to right: **1, 2** - “Control”; **3, 4** - “Stroke+Saline”, 7 days after PT; **5, 6** - “Stroke+Mexidol”, 7 days after PT; **7, 8** - intact rats receiving a 7-days course of Mexidol; **9, 10** - “Stroke+Saline”, 21 days after PT; **11, 12** - “Stroke+Mexidol”, 14 days after withdrawal of Mexidol treatment; **13, 14** - intact rats, 14 days after withdrawal of Mexidol treatment.

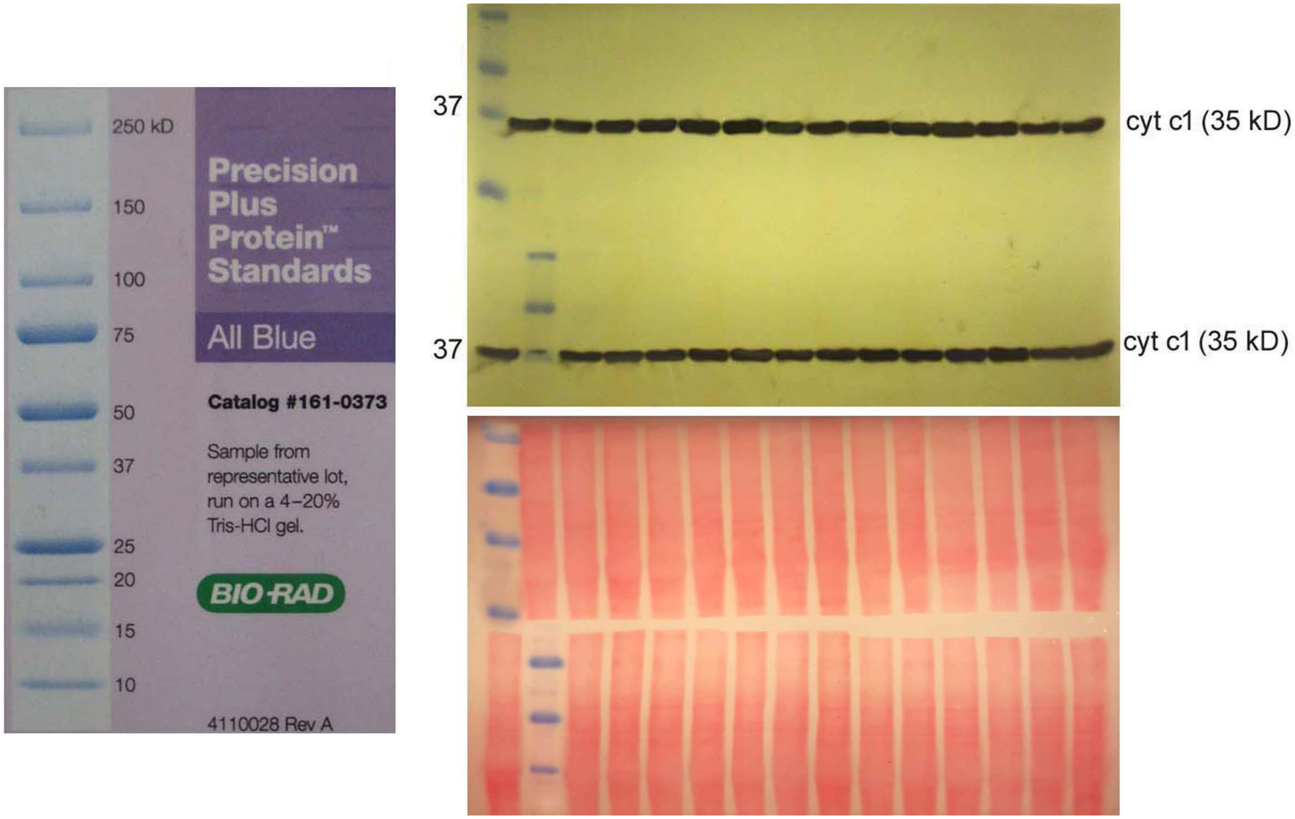

Supplementary Figure 13. Representative immunoblot image (Rg-film combined with nitrocellulose membrane) showing expression of cyt c1 in the prefrontal cortex penumbra from rats with photochemically induced thrombosis (PT) (“Stroke + Saline” group), rats treated with Mexidol by i/p injection 2 h after PT and for the consecutive 6 days (1 injection per day) at a dose 100 mg/kg (“Stroke + Mexidol” group), and intact rats treated with a 7-day course of Mexidol, for 21 days of observation. **Top sequence of blots** (first gel; one animal corresponds to each blot), from left to right: **1, 2** - “Control” (rats received saline according to the same regimen as in the “Stroke+Mexidol” group); **3, 4** - “Stroke+Saline”, 24 h after PT; **5, 6** - “Stroke+Mexidol”, 24 h after PT; **7, 8** - intact rats receiving a single injection of Mexidol; **9, 10** - “Stroke+Saline”, 3 days after PT; **11, 12** - “Stroke+Mexidol”; 3 days after PT; **13, 14** - intact rats receiving a 3-days course of Mexidol; **Bottom sequence of blots** (second gel one; animal corresponds to each blot), from left to right: **1, 2** - “Control”; **3, 4** - “Stroke+Saline”, 7 days after PT; **5, 6** - “Stroke+Mexidol”, 7 days after PT; **7, 8** - intact rats receiving a 7-days course of Mexidol; **9, 10** - “Stroke+Saline”, 21 days after PT; **11, 12** - “Stroke+Mexidol”, 14 days after withdrawal of Mexidol treatment; **13, 14** - intact rats, 14 days after withdrawal of Mexidol treatment.

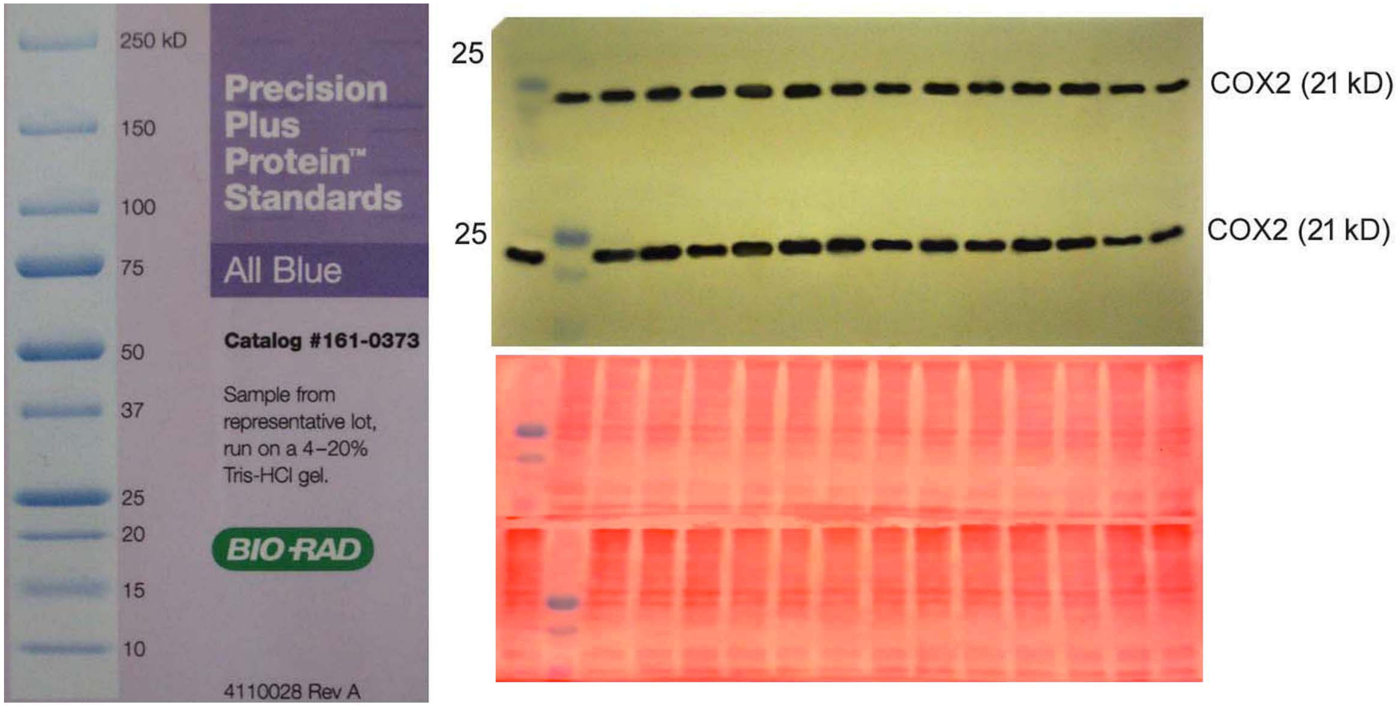

Supplementary Figure 14. Representative immunoblot image (Rg-film combined with nitrocellulose membrane) showing expression of COX2 in the prefrontal cortex penumbra from rats with photochemically induced thrombosis (PT) (“Stroke + Saline” group), rats treated with Mexidol by i/p injection 2 h after PT and for the consecutive 6 days (1 injection per day) at a dose 100 mg/kg (“Stroke + Mexidol” group), and intact rats treated with a 7-day course of Mexidol, for 21 days of observation. **Top sequence of blots** (first gel; one animal corresponds to each blot), from left to right: **1, 2** - “Control” (rats received saline according to the same regimen as in the “Stroke+Mexidol” group); **3, 4** - “Stroke+Saline”, 24 h after PT; **5, 6** - “Stroke+Mexidol”, 24 h after PT; **7, 8** - intact rats receiving a single injection of Mexidol; **9, 10** - “Stroke+Saline”, 3 days after PT; **11, 12** - “Stroke+Mexidol”; 3 days after PT; **13, 14** - intact rats receiving a 3-days course of Mexidol; **Bottom sequence of blots** (second gel one; animal corresponds to each blot), from left to right: **1, 2** - “Control”; **3, 4** - “Stroke+Saline”, 7 days after PT; **5, 6** - “Stroke+Mexidol”, 7 days after PT; **7, 8** - intact rats receiving a 7-days course of Mexidol; **9, 10** - “Stroke+Saline”, 21 days after PT; **11, 12** - “Stroke+Mexidol”, 14 days after withdrawal of Mexidol treatment; **13, 14** - intact rats, 14 days after withdrawal of Mexidol treatment.

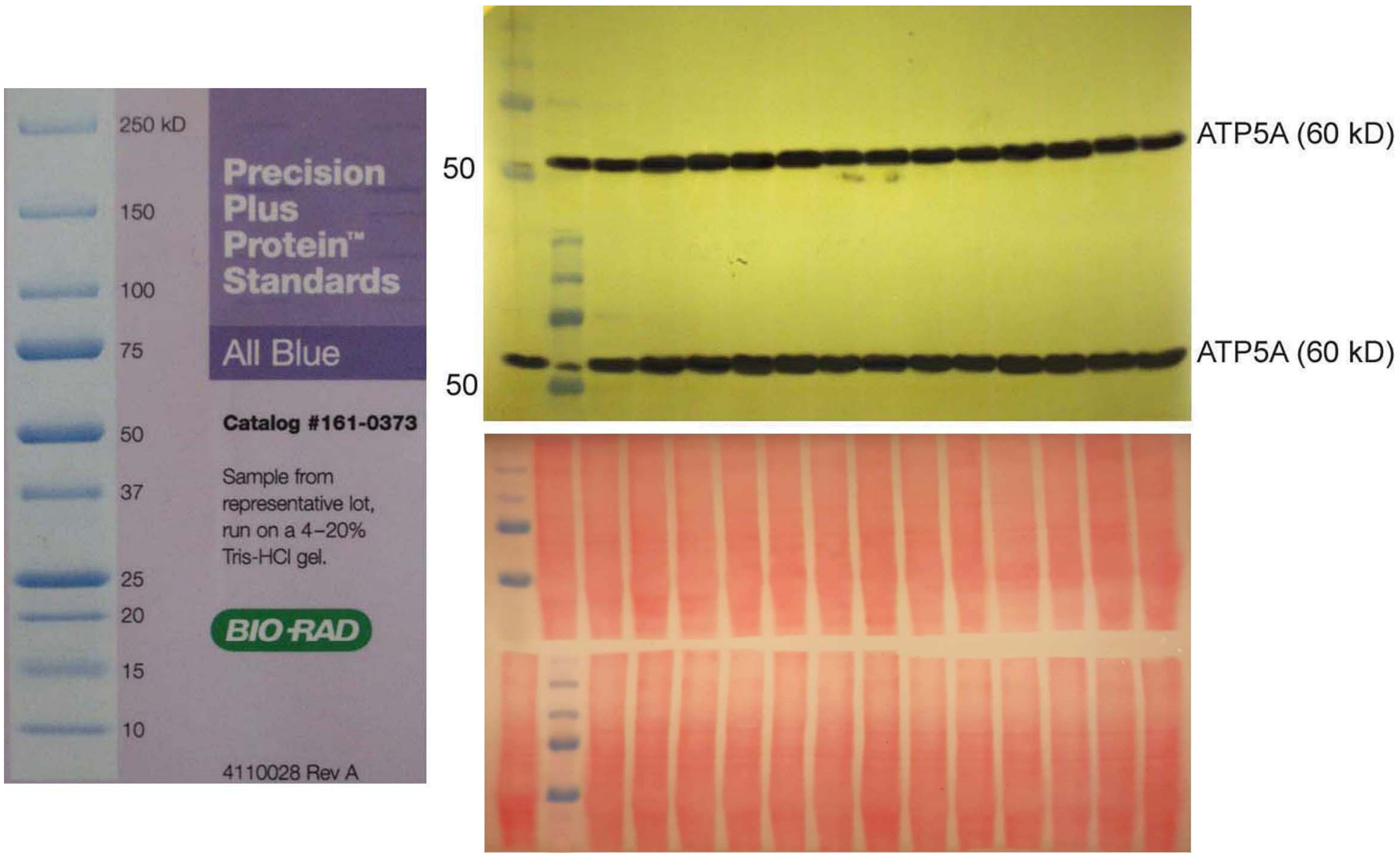

Supplementary Figure 15. Representative immunoblot image (Rg-film combined with nitrocellulose membrane) showing expression of ATP5A in the prefrontal cortex penumbra from rats with photochemically induced thrombosis (PT) ("Stroke + Saline" group), rats treated with Mexidol by i/p injection 2 h after PT and for the consecutive 6 days (1 injection per day) at a dose 100 mg/kg ("Stroke + Mexidol" group), and intact rats treated with a 7-day course of Mexidol, for 21 days of observation. **Top sequence of blots** (first gel; one animal corresponds to each blot), from left to right: **1, 2** - "Control" (rats received saline according to the same regimen as in the "Stroke+Mexidol" group); **3, 4** - "Stroke+Saline", 24 h after PT; **5, 6** - "Stroke+Mexidol", 24 h after PT; **7, 8** - intact rats receiving a single injection of Mexidol; **9, 10** - "Stroke+Saline", 3 days after PT; **11, 12** - "Stroke+Mexidol"; 3 days after PT; **13, 14** - intact rats receiving a 3-days course of Mexidol; **Bottom sequence of blots** (second gel one; animal corresponds to each blot), from left to right: **1, 2** - "Control"; **3, 4** - "Stroke+Saline", 7 days after PT; **5, 6** - "Stroke+Mexidol", 7 days after PT; **7, 8** - intact rats receiving a 7-days course of Mexidol; **9, 10** - "Stroke+Saline", 21 days after PT; **11, 12** - "Stroke+Mexidol", 14 days after withdrawal of Mexidol treatment; **13, 14** - intact rats, 14 days after withdrawal of Mexidol treatment.

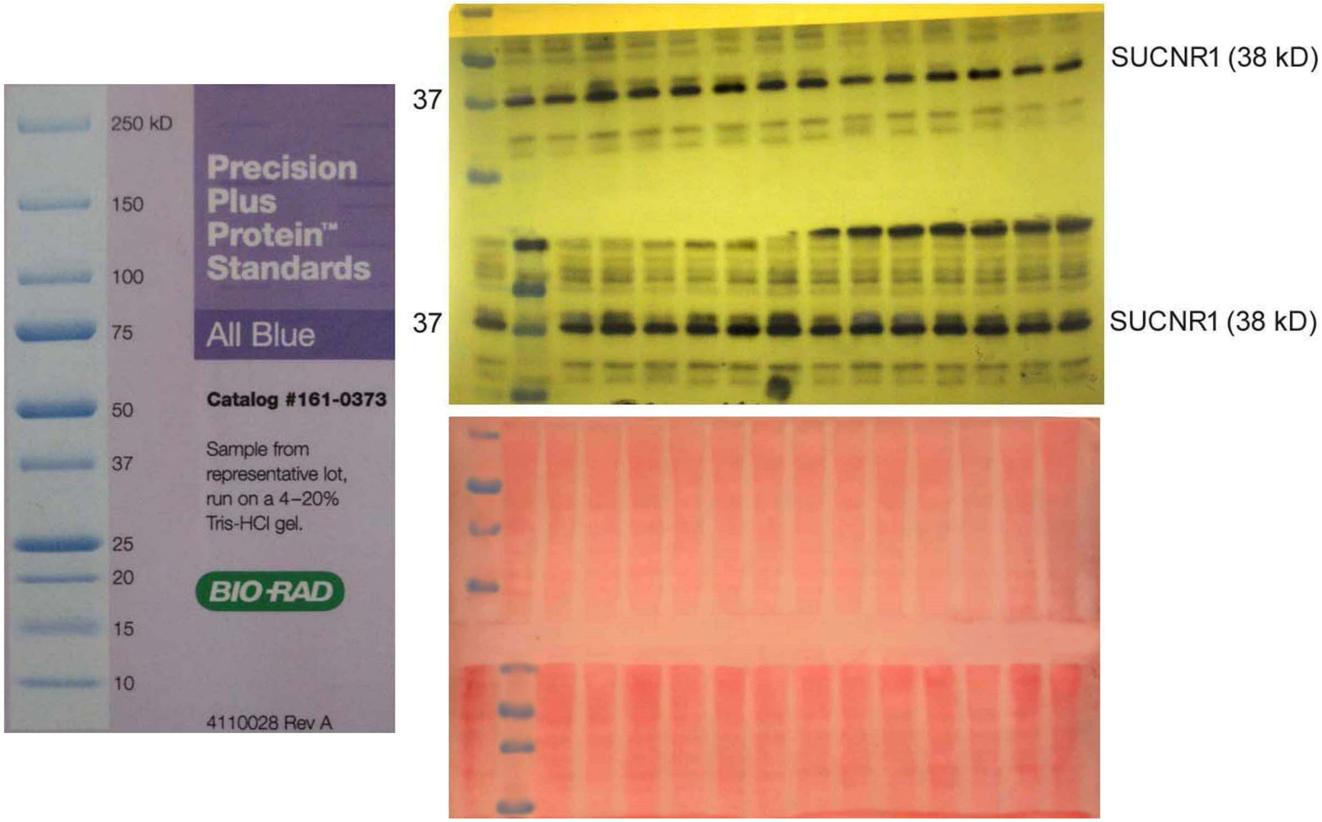

Supplementary Figure 16. Representative immunoblot image (Rg-film combined with nitrocellulose membrane) showing expression of SUCNR1 in the prefrontal cortex penumbra from rats with photochemically induced thrombosis (PT) ("Stroke + Saline" group), rats treated with Mexidol by i/p injection 2 h after PT and for the consecutive 6 days (1 injection per day) at a dose 100 mg/kg ("Stroke + Mexidol" group), and intact rats treated with a 7-day course of Mexidol, for 21 days of observation. **Top sequence of blots** (first gel; one animal corresponds to each blot), from left to right: **1, 2** - "Control" (rats received saline according to the same regimen as in the "Stroke+Mexidol" group); **3, 4** - "Stroke+Saline", 24 h after PT; **5, 6** - "Stroke+Mexidol", 24 h after PT; **7, 8** - intact rats receiving a single injection of Mexidol; **9, 10** - "Stroke+Saline", 3 days after PT; **11, 12** - "Stroke+Mexidol"; 3 days after PT; **13, 14** - intact rats receiving a 3-days course of Mexidol; **Bottom sequence of blots** (second gel one; animal corresponds to each blot), from left to right: **1, 2** - "Control"; **3, 4** - "Stroke+Saline", 7 days after PT; **5, 6** - "Stroke+Mexidol", 7 days after PT; **7, 8** - intact rats receiving a 7-days course of Mexidol; **9, 10** - "Stroke+Saline", 21 days after PT; **11, 12** - "Stroke+Mexidol", 14 days after withdrawal of Mexidol treatment; **13, 14** - intact rats, 14 days after withdrawal of Mexidol treatment.

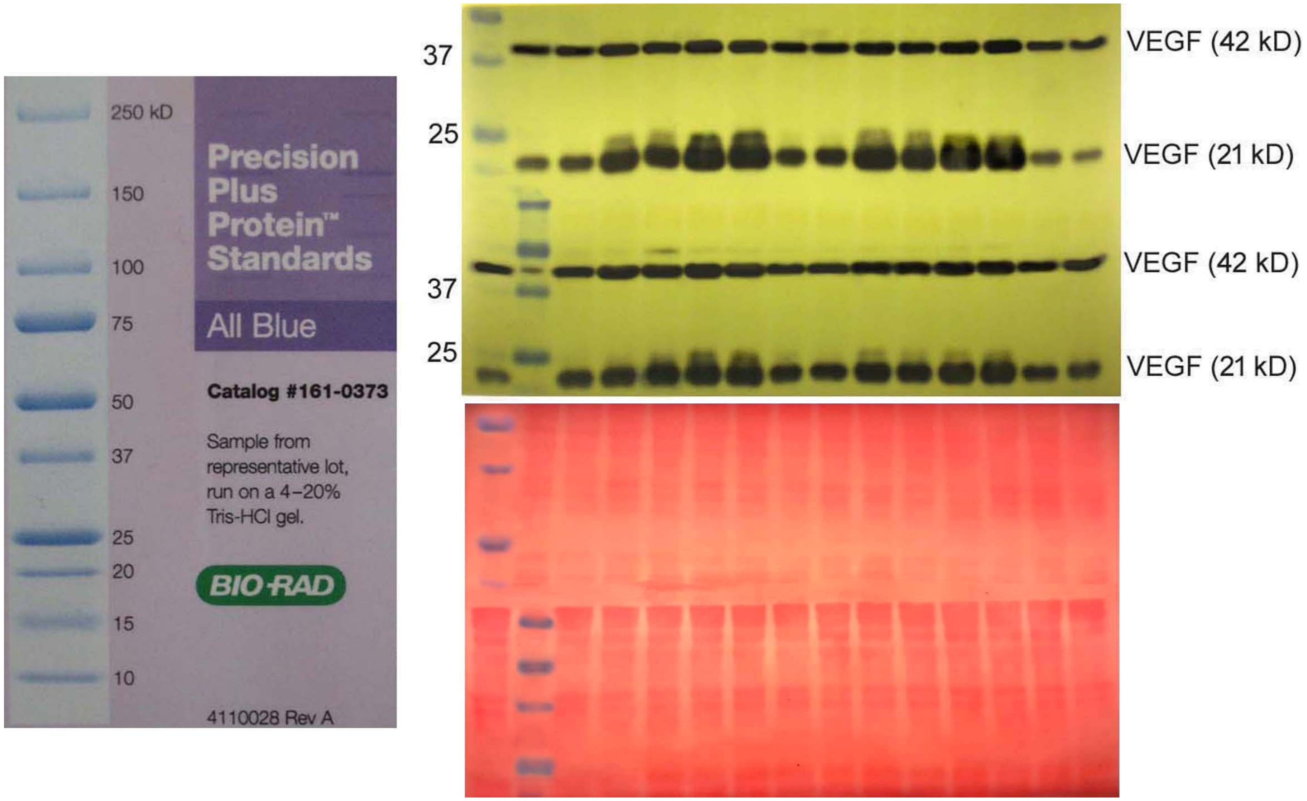

Supplementary Figure 17. Representative immunoblot image (Rg-film combined with nitrocellulose membrane) showing expression of VEGF in the prefrontal cortex penumbra from rats with photochemically induced thrombosis (PT) (“Stroke + Saline” group), rats treated with Mexidol by i/p injection 2 h after PT and for the consecutive 6 days (1 injection per day) at a dose 100 mg/kg (“Stroke + Mexidol” group), and intact rats treated with a 7-day course of Mexidol, for 21 days of observation. **Top sequence of blots** (first gel; one animal corresponds to each blot), from left to right: **1, 2** - “Control” (rats received saline according to the same regimen as in the “Stroke+Mexidol” group); **3, 4** - “Stroke+Saline”, 24 h after PT; **5, 6** - “Stroke+Mexidol”, 24 h after PT; **7, 8** - intact rats receiving a single injection of Mexidol; **9, 10** - “Stroke+Saline”, 3 days after PT; **11, 12** - “Stroke+Mexidol”; 3 days after PT; **13, 14** - intact rats receiving a 3-days course of Mexidol; **Bottom sequence of blots** (second gel one; animal corresponds to each blot), from left to right: **1, 2** - “Control”; **3, 4** - “Stroke+Saline”, 7 days after PT; **5, 6** - “Stroke+Mexidol”, 7 days after PT; **7, 8** - intact rats receiving a 7-days course of Mexidol; **9, 10** - “Stroke+Saline”, 21 days after PT; **11, 12** - “Stroke+Mexidol”, 14 days after withdrawal of Mexidol treatment; **13, 14** - intact rats, 14 days after withdrawal of Mexidol treatment.

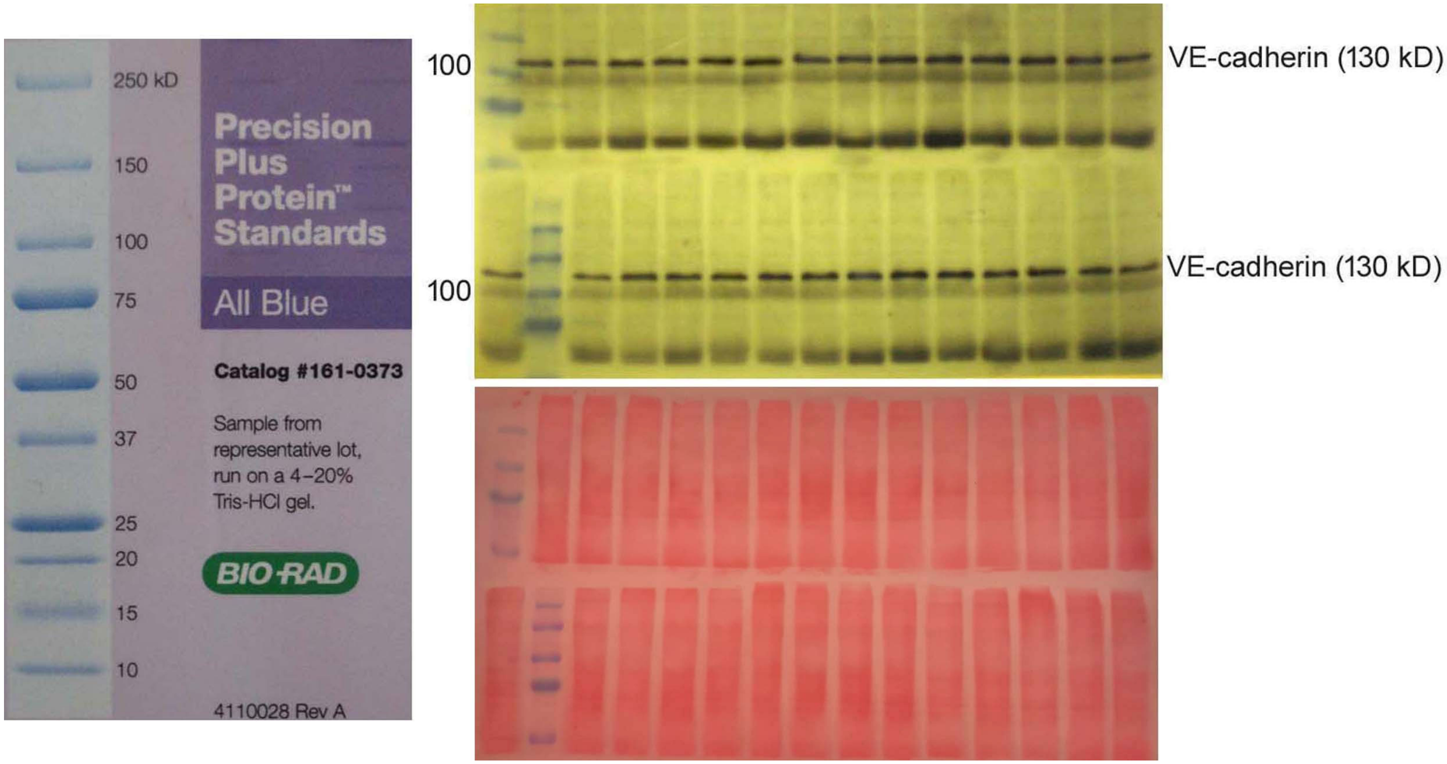

Supplementary Figure 18. Representative immunoblot image (Rg-film combined with nitrocellulose membrane) showing expression of VE-cadherin in the prefrontal cortex penumbra from rats with photochemically induced thrombosis (PT) ("Stroke + Saline" group), rats treated with Mexidol by i/p injection 2 h after PT and for the consecutive 6 days (1 injection per day) at a dose 100 mg/kg ("Stroke + Mexidol" group), and intact rats treated with a 7-day course of Mexidol, for 21 days of observation. **Top sequence of blots** (first gel; one animal corresponds to each blot), from left to right: **1, 2** - "Control" (rats received saline according to the same regimen as in the "Stroke+Mexidol" group); **3, 4** - "Stroke+Saline", 24 h after PT; **5, 6** - "Stroke+Mexidol", 24 h after PT; **7, 8** - intact rats receiving a single injection of Mexidol; **9, 10** - "Stroke+Saline", 3 days after PT; **11, 12** - "Stroke+Mexidol"; 3 days after PT; **13, 14** - intact rats receiving a 3-days course of Mexidol; **Bottom sequence of blots** (second gel one; animal corresponds to each blot), from left to right: **1, 2** - "Control"; **3, 4** - "Stroke+Saline", 7 days after PT; **5, 6** - "Stroke+Mexidol", 7 days after PT; **7, 8** - intact rats receiving a 7-days course of Mexidol; **9, 10** - "Stroke+Saline", 21 days after PT; **11, 12** - "Stroke+Mexidol", 14 days after withdrawal of Mexidol treatment; **13, 14** - intact rats, 14 days after withdrawal of Mexidol treatment.

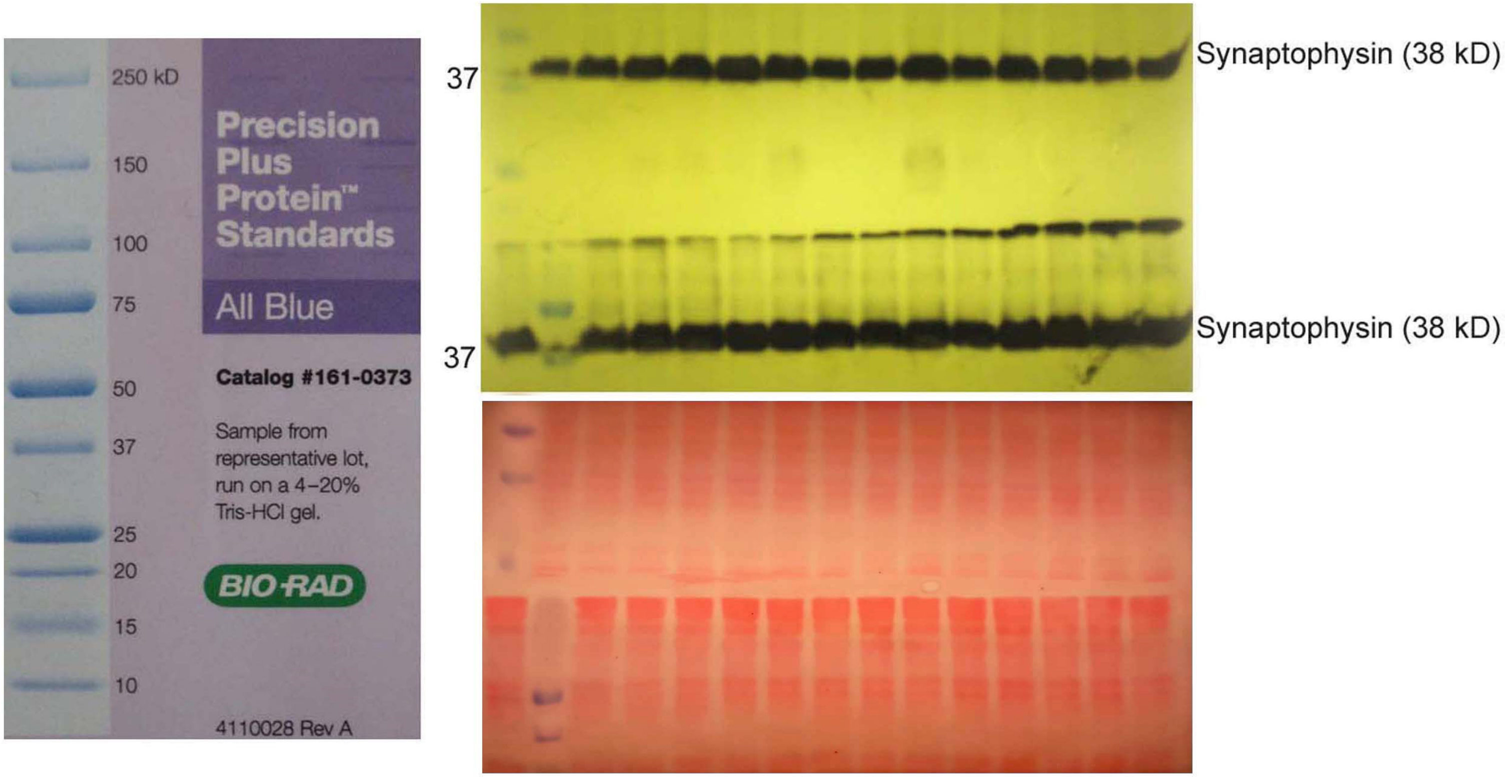

Supplementary Figure 19. Representative immunoblot image (Rg-film combined with nitrocellulose membrane) showing expression of Synaptophysin in the prefrontal cortex penumbra from rats with photochemically induced thrombosis (PT) ("Stroke + Saline" group), rats treated with Mexidol by i/p injection 2 h after PT and for the consecutive 6 days (1 injection per day) at a dose 100 mg/kg ("Stroke + Mexidol" group), and intact rats treated with a 7-day course of Mexidol, for 21 days of observation. **Top sequence of blots** (first gel; one animal corresponds to each blot), from left to right: **1, 2** - "Control" (rats received saline according to the same regimen as in the "Stroke+Mexidol" group); **3, 4** - "Stroke+Saline", 24 h after PT; **5, 6** - "Stroke+Mexidol", 24 h after PT; **7, 8** - intact rats receiving a single injection of Mexidol; **9, 10** - "Stroke+Saline", 3 days after PT; **11, 12** - "Stroke+Mexidol"; 3 days after PT; **13, 14** - intact rats receiving a 3-days course of Mexidol; **Bottom sequence of blots** (second gel one; animal corresponds to each blot), from left to right: **1, 2** - "Control"; **3, 4** - "Stroke+Saline", 7 days after PT; **5, 6** - "Stroke+Mexidol", 7 days after PT; **7, 8** - intact rats receiving a 7-days course of Mexidol; **9, 10** - "Stroke+Saline", 21 days after PT; **11, 12** - "Stroke+Mexidol", 14 days after withdrawal of Mexidol treatment; **13, 14** - intact rats, 14 days after withdrawal of Mexidol treatment.

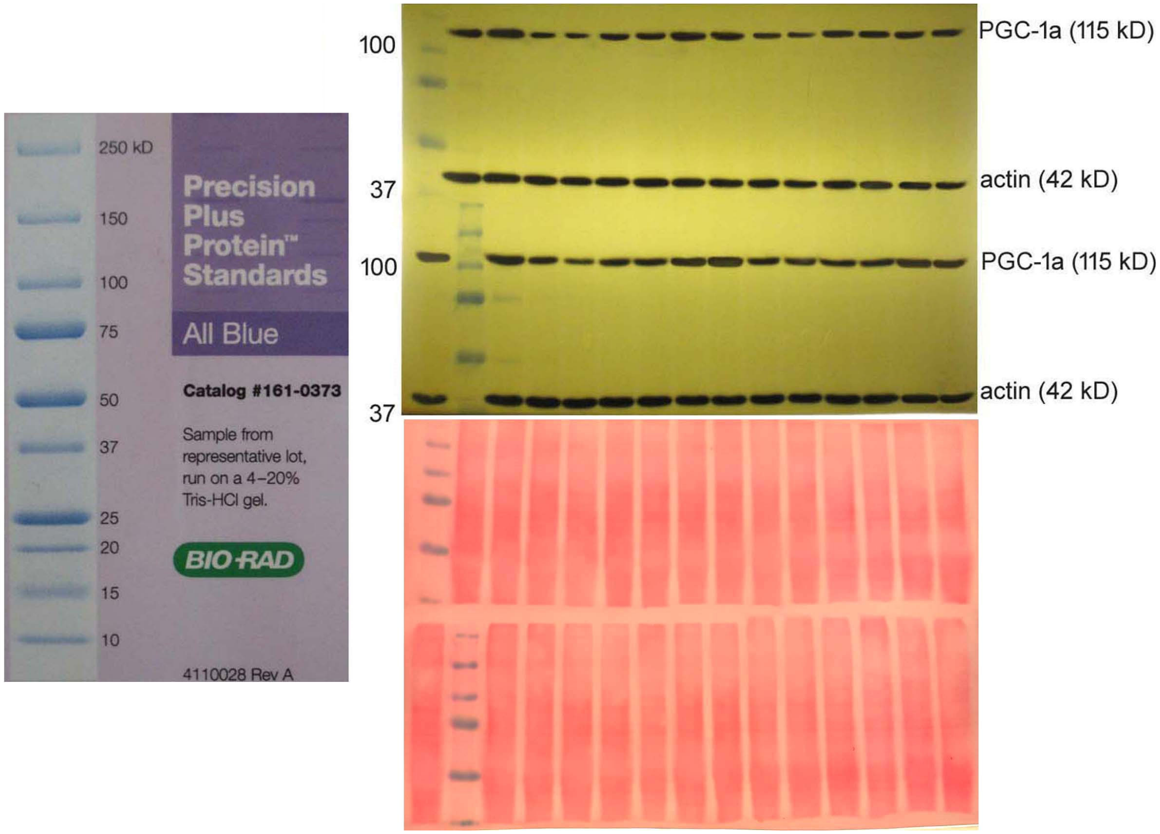

Supplementary Figure 20. Representative immunoblot image (Rg-film combined with nitrocellulose membrane) showing expression of PGC-1α in the prefrontal cortex penumbra from rats with photochemically induced thrombosis (PT) (“Stroke + Saline” group), rats treated with Semax by i/n administration 2 h after PT and for the consecutive 6 days (1 instillation per day) at a dose 25 µg/kg (“Stroke + Semax” group), and intact rats treated with a 7-day course of Semax, for 21 days of observation. **Top sequence of blots** (first gel; one animal corresponds to each blot), from left to right: **1, 2** - “Control” (rats received saline); **3, 4** - “Stroke+Saline”, 24 h after PT; **5, 6** - “Stroke+Semax”, 24 h after PT; **7, 8** - intact rats receiving a single injection of Semax; **9, 10** - “Stroke+Saline”, 3 days after PT; **11, 12** - “Stroke+Semax”; 3 days after PT; **13, 14** - intact rats receiving a 3-days course of Semax; **Bottom sequence of blots** (second gel one; animal corresponds to each blot), from left to right: **1, 2** - “Control”; **3, 4** - “Stroke+Saline”, 7 days after PT; **5, 6** - “Stroke+Semax”, 7 days after PT; **7, 8** - intact rats receiving a 7-days course of Semax; **9, 10** - “Stroke+Saline”, 21 days after PT; **11, 12** - “Stroke+Semax”, 14 days after withdrawal of Semax treatment; **13, 14** - intact rats, 14 days after withdrawal of Semax treatment.

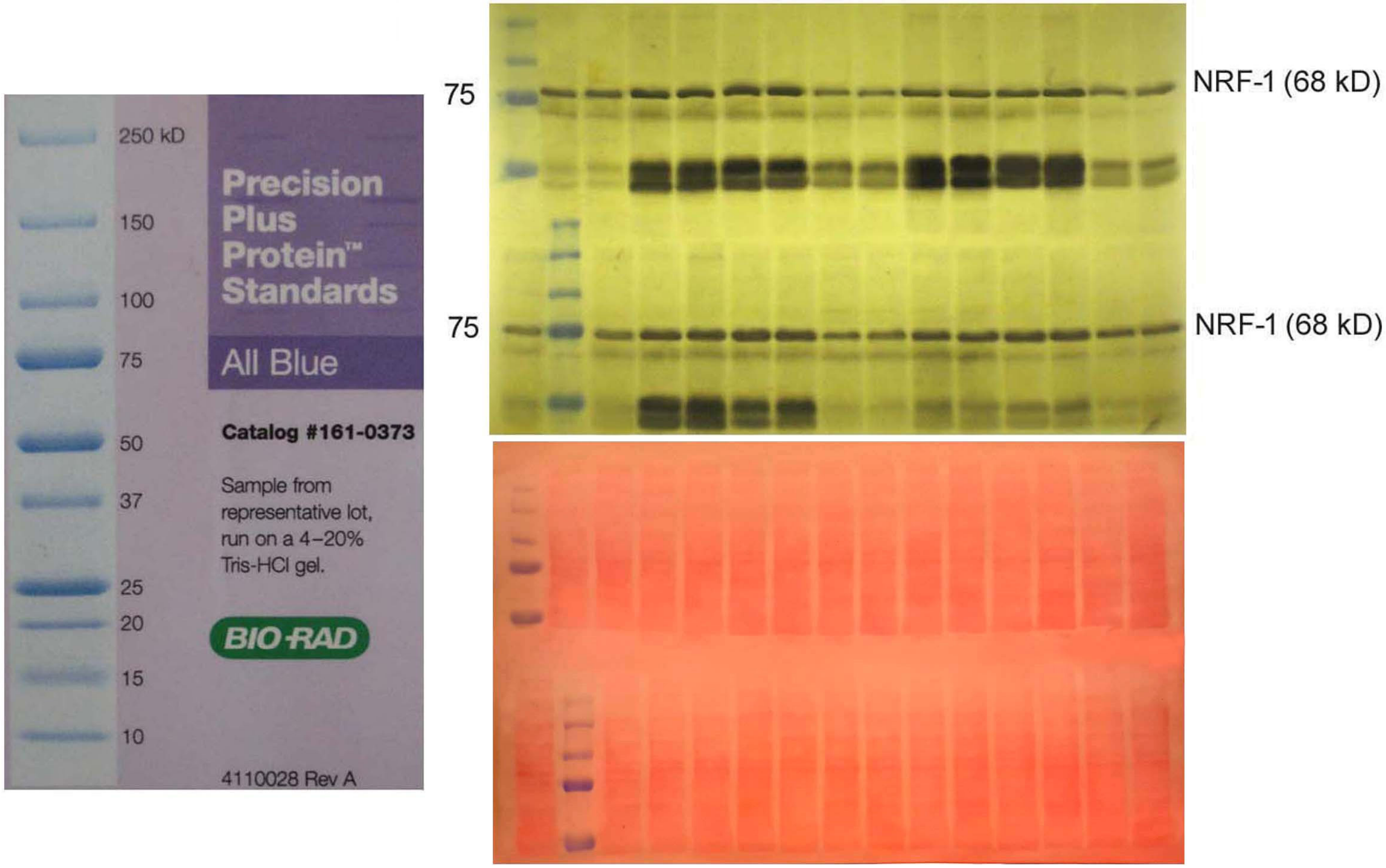

Supplementary Figure 21. Representative immunoblot image (Rg-film combined with nitrocellulose membrane) showing expression of NRF1 in the prefrontal cortex penumbra from rats with photochemically induced thrombosis (PT) (“Stroke + Saline” group), rats treated with Semax by i/n administration 2 h after PT and for the consecutive 6 days (1 instillation per day) at a dose 25 μg/kg (“Stroke + Semax” group), and intact rats treated with a 7-day course of Semax, for 21 days of observation. **Top sequence of blots** (first gel; one animal corresponds to each blot), from left to right: **1, 2** - “Control” (rats received saline); **3, 4** - “Stroke+Saline”, 24 h after PT; **5, 6** - “Stroke+Semax”, 24 h after PT; **7, 8** - intact rats receiving a single injection of Semax; **9, 10** - “Stroke+Saline”, 3 days after PT; **11, 12** - “Stroke+Semax”; 3 days after PT; **13, 14** - intact rats receiving a 3-days course of Semax; **Bottom sequence of blots** (second gel one; animal corresponds to each blot), from left to right: **1, 2** - “Control”; **3, 4** - “Stroke+Saline”, 7 days after PT; **5, 6** - “Stroke+Semax”, 7 days after PT; **7, 8** - intact rats receiving a 7-days course of Semax; **9, 10** - “Stroke+Saline”, 21 days after PT; **11, 12** - “Stroke+Semax”, 14 days after withdrawal of Semax treatment; **13, 14** - intact rats, 14 days after withdrawal of Semax treatment.

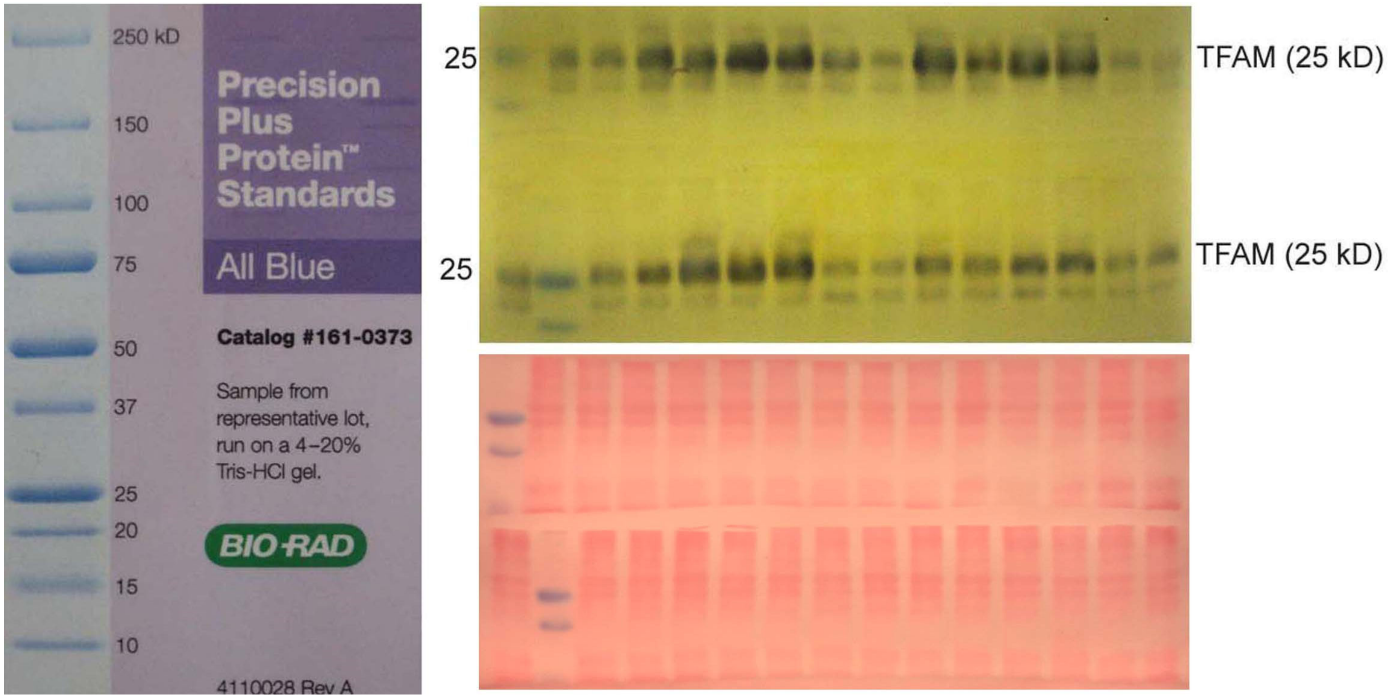

Supplementary Figure 22. Representative immunoblot image (Rg-film combined with nitrocellulose membrane) showing expression of TFAM in the prefrontal cortex penumbra from rats with photochemically induced thrombosis (PT) ("Stroke + Saline" group), rats treated with Semax by i/n administration 2 h after PT and for the consecutive 6 days (1 instillation per day) at a dose 25 μg/kg ("Stroke + Semax" group), and intact rats treated with a 7-day course of Semax, for 21 days of observation. **Top sequence of blots** (first gel; one animal corresponds to each blot), from left to right: **1, 2** - "Control" (rats received saline); **3, 4** - "Stroke+Saline", 24 h after PT; **5, 6** - "Stroke+Semax", 24 h after PT; **7, 8** - intact rats receiving a single injection of Semax; **9, 10** - "Stroke+Saline", 3 days after PT; **11, 12** - "Stroke+Semax"; 3 days after PT; **13, 14** - intact rats receiving a 3-days course of Semax; **Bottom sequence of blots** (second gel one; animal corresponds to each blot), from left to right: **1, 2** - "Control"; **3, 4** - "Stroke+Saline", 7 days after PT; **5, 6** - "Stroke+Semax", 7 days after PT; **7, 8** - intact rats receiving a 7-days course of Semax; **9, 10** - "Stroke+Saline", 21 days after PT; **11, 12** - "Stroke+Semax", 14 days after withdrawal of Semax treatment; **13, 14** - intact rats, 14 days after withdrawal of Semax treatment.

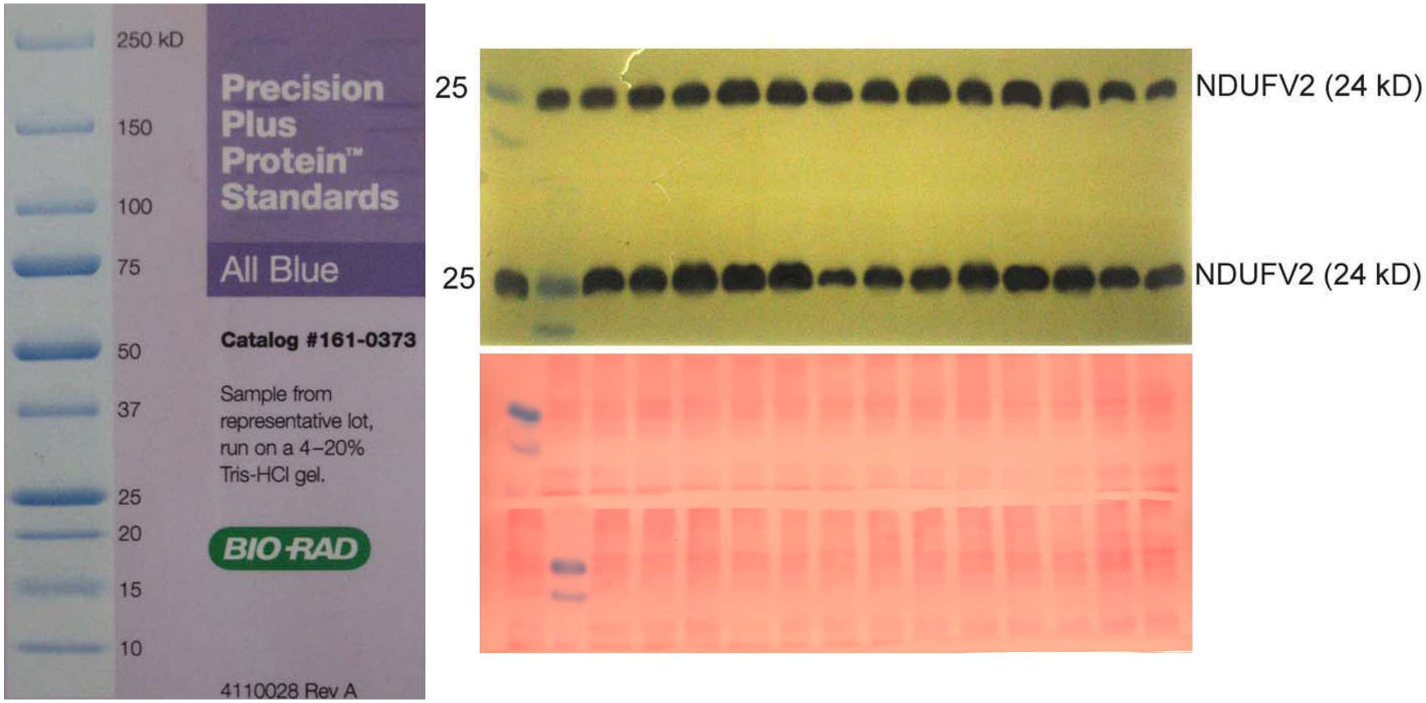

Supplementary Figure 23. Representative immunoblot image (Rg-film combined with nitrocellulose membrane) showing expression of NDUFV2 in the prefrontal cortex penumbra from rats with photochemically induced thrombosis (PT) (“Stroke + Saline” group), rats treated with Semax by i/n administration 2 h after PT and for the consecutive 6 days (1 instillation per day) at a dose 25 µg/kg (“Stroke + Semax” group), and intact rats treated with a 7-day course of Semax, for 21 days of observation. **Top sequence of blots** (first gel; one animal corresponds to each blot), from left to right: **1, 2** - “Control” (rats received saline); **3, 4** - “Stroke+Saline”, 24 h after PT; **5, 6** - “Stroke+Semax”, 24 h after PT; **7, 8** - intact rats receiving a single injection of Semax; **9, 10** - “Stroke+Saline”, 3 days after PT; **11, 12** - “Stroke+Semax”; 3 days after PT; **13, 14** - intact rats receiving a 3-days course of Semax; **Bottom sequence of blots** (second gel one; animal corresponds to each blot), from left to right: **1, 2** - “Control”; **3, 4** - “Stroke+Saline”, 7 days after PT; **5, 6** - “Stroke+Semax”, 7 days after PT; **7, 8** - intact rats receiving a 7-days course of Semax; **9, 10** - “Stroke+Saline”, 21 days after PT; **11, 12** - “Stroke+Semax”, 14 days after withdrawal of Semax treatment; **13, 14** - intact rats, 14 days after withdrawal of Semax treatment.

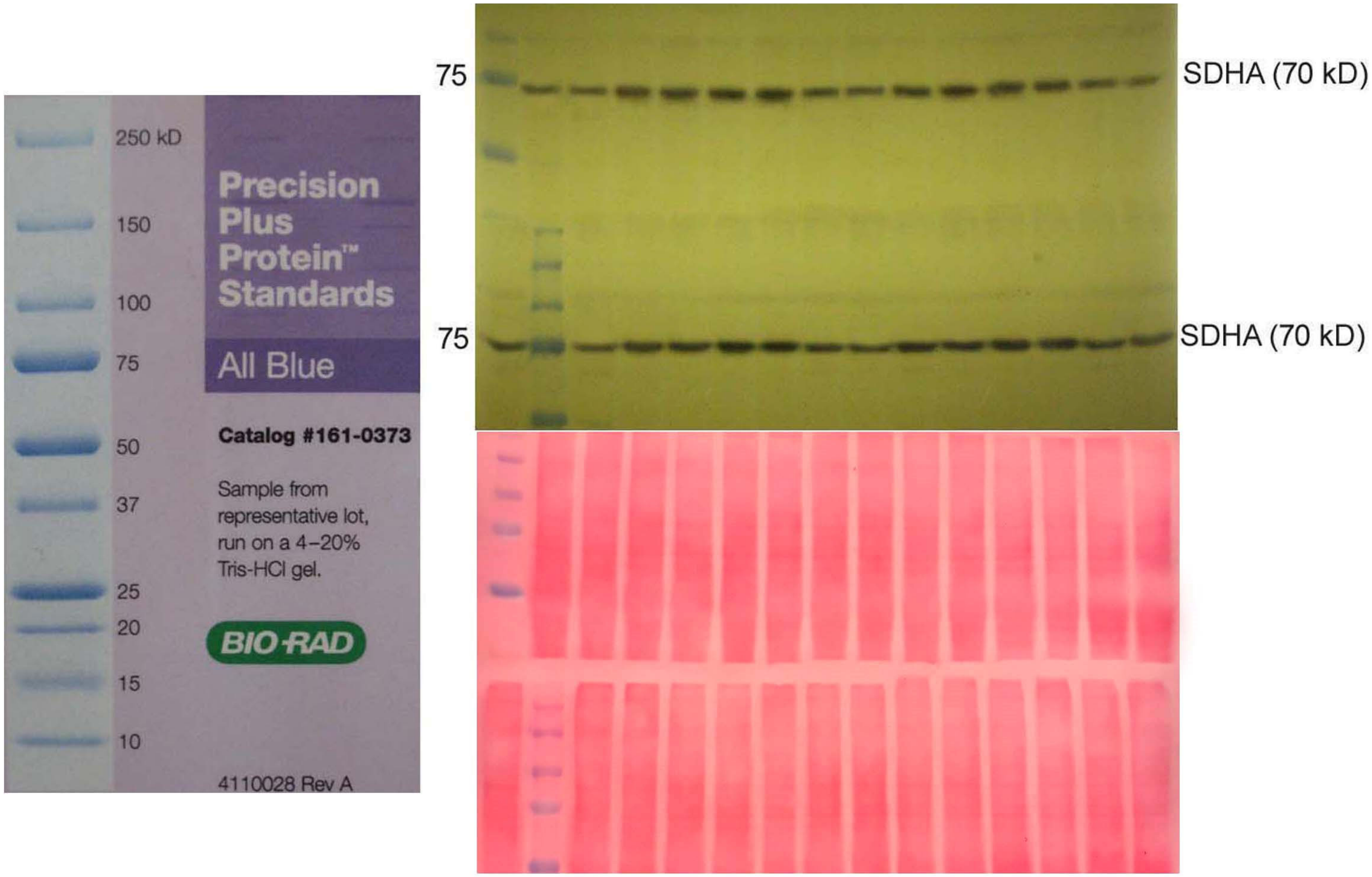

Supplementary Figure 24. Representative immunoblot image (Rg-film combined with nitrocellulose membrane) showing expression of SDHA in the prefrontal cortex penumbra from rats with photochemically induced thrombosis (PT) ("Stroke + Saline" group), rats treated with Semax by i/n administration 2 h after PT and for the consecutive 6 days (1 instillation per day) at a dose 25 µg/kg ("Stroke + Semax" group), and intact rats treated with a 7-day course of Semax, for 21 days of observation. **Top sequence of blots** (first gel; one animal corresponds to each blot), from left to right: **1, 2** - "Control" (rats received saline); **3, 4** - "Stroke+Saline", 24 h after PT; **5, 6** - "Stroke+Semax", 24 h after PT; **7, 8** - intact rats receiving a single injection of Semax; **9, 10** - "Stroke+Saline", 3 days after PT; **11, 12** - "Stroke+Semax"; 3 days after PT; **13, 14** - intact rats receiving a 3-days course of Semax; **Bottom sequence of blots** (second gel one; animal corresponds to each blot), from left to right: **1, 2** - "Control"; **3, 4** - "Stroke+Saline", 7 days after PT; **5, 6** - "Stroke+Semax", 7 days after PT; **7, 8** - intact rats receiving a 7-days course of Semax; **9, 10** - "Stroke+Saline", 21 days after PT; **11, 12** - "Stroke+Semax", 14 days after withdrawal of Semax treatment; **13, 14** - intact rats, 14 days after withdrawal of Semax treatment.

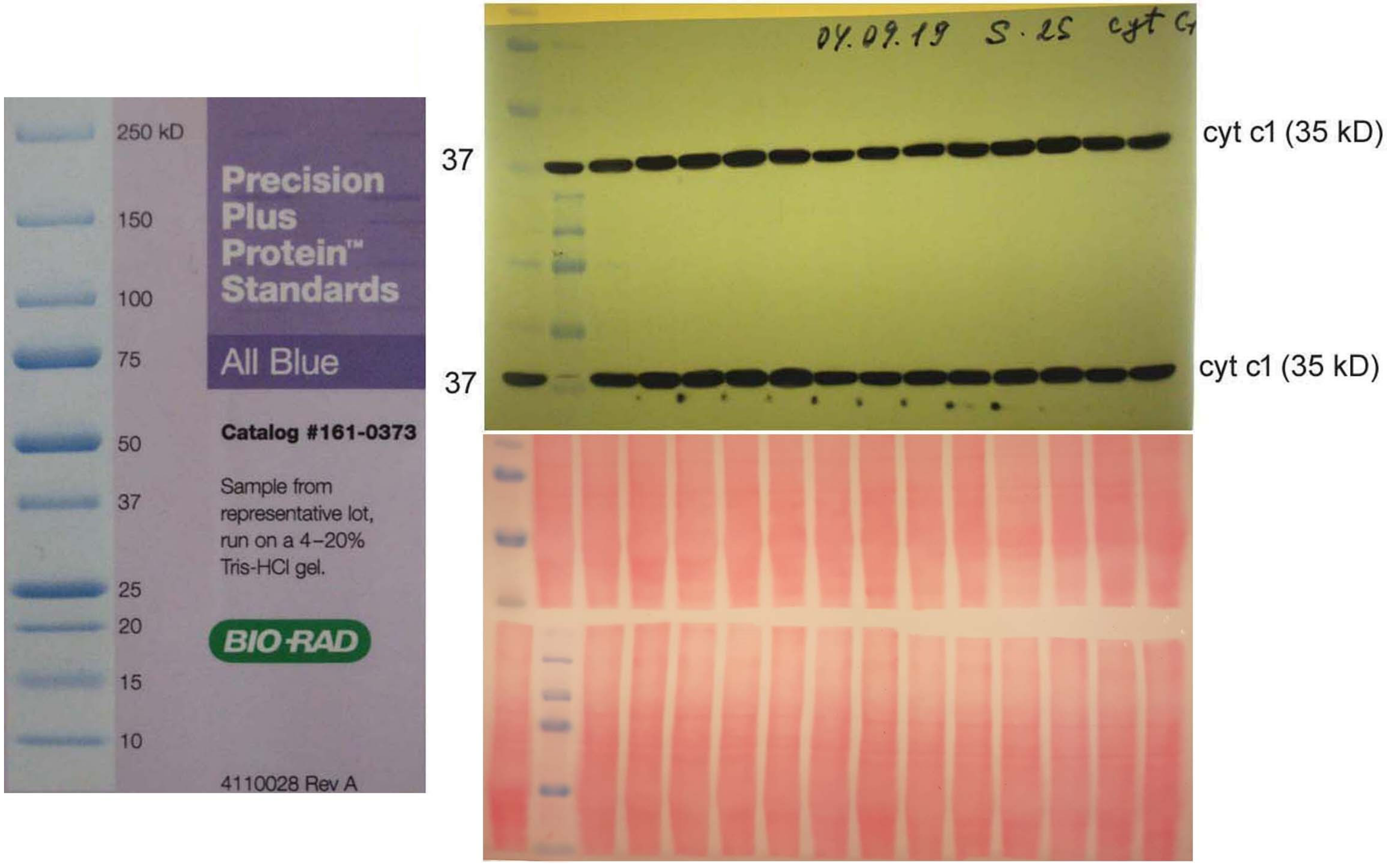

Supplementary Figure 25. Representative immunoblot image (Rg-film combined with nitrocellulose membrane) showing expression of cyt c1 in the prefrontal cortex penumbra from rats with photochemically induced thrombosis (PT) ("Stroke + Saline" group), rats treated with Semax by i/n administration 2 h after PT and for the consecutive 6 days (1 instillation per day) at a dose 25 µg/kg ("Stroke + Semax" group), and intact rats treated with a 7-day course of Semax, for 21 days of observation. **Top sequence of blots** (first gel; one animal corresponds to each blot), from left to right: **1, 2** - "Control" (rats received saline); **3, 4** - "Stroke+Saline", 24 h after PT; **5, 6** - "Stroke+Semax", 24 h after PT; **7, 8** - intact rats receiving a single injection of Semax; **9, 10** - "Stroke+Saline", 3 days after PT; **11, 12** - "Stroke+Semax"; 3 days after PT; **13, 14** - intact rats receiving a 3-days course of Semax; **Bottom sequence of blots** (second gel one; animal corresponds to each blot), from left to right: **1, 2** - "Control"; **3, 4** - "Stroke+Saline", 7 days after PT; **5, 6** - "Stroke+Semax", 7 days after PT; **7, 8** - intact rats receiving a 7-days course of Semax; **9, 10** - "Stroke+Saline", 21 days after PT; **11, 12** - "Stroke+Semax", 14 days after withdrawal of Semax treatment; **13, 14** - intact rats, 14 days after withdrawal of Semax treatment.

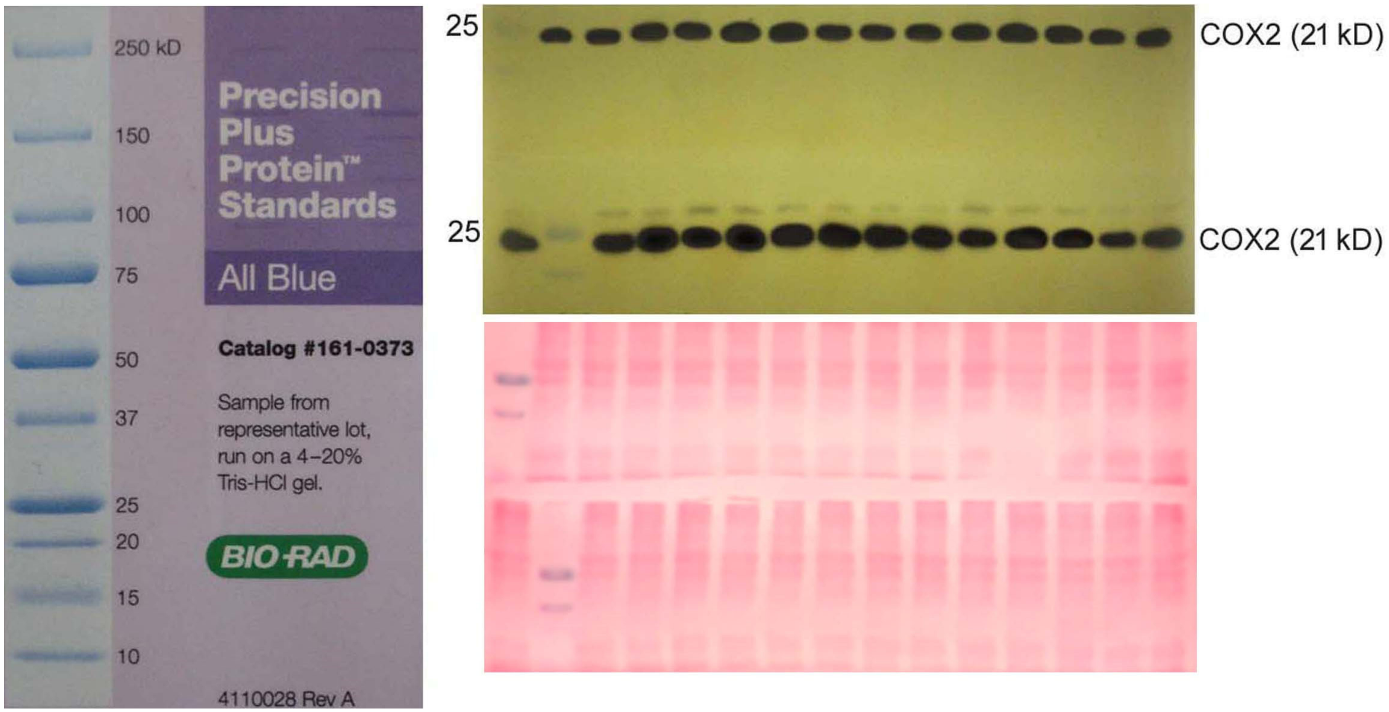

Supplementary Figure 26. Representative immunoblot image (Rg-film combined with nitrocellulose membrane) showing expression of COX2 in the prefrontal cortex penumbra from rats with photochemically induced thrombosis (PT) (“Stroke + Saline” group), rats treated with Semax by i/n administration 2 h after PT and for the consecutive 6 days (1 instillation per day) at a dose 25 µg/kg (“Stroke + Semax” group), and intact rats treated with a 7-day course of Semax, for 21 days of observation. **Top sequence of blots** (first gel; one animal corresponds to each blot), from left to right: **1, 2** - “Control” (rats received saline); **3, 4** - “Stroke+Saline”, 24 h after PT; **5, 6** - “Stroke+Semax”, 24 h after PT; **7, 8** - intact rats receiving a single injection of Semax; **9, 10** - “Stroke+Saline”, 3 days after PT; **11, 12** - “Stroke+Semax”; 3 days after PT; **13, 14** - intact rats receiving a 3-days course of Semax; **Bottom sequence of blots** (second gel one; animal corresponds to each blot), from left to right: **1, 2** - “Control”; **3, 4** - “Stroke+Saline”, 7 days after PT; **5, 6** - “Stroke+Semax”, 7 days after PT; **7, 8** - intact rats receiving a 7-days course of Semax; **9, 10** - “Stroke+Saline”, 21 days after PT; **11, 12** - “Stroke+Semax”, 14 days after withdrawal of Semax treatment; **13, 14** - intact rats, 14 days after withdrawal of Semax treatment.

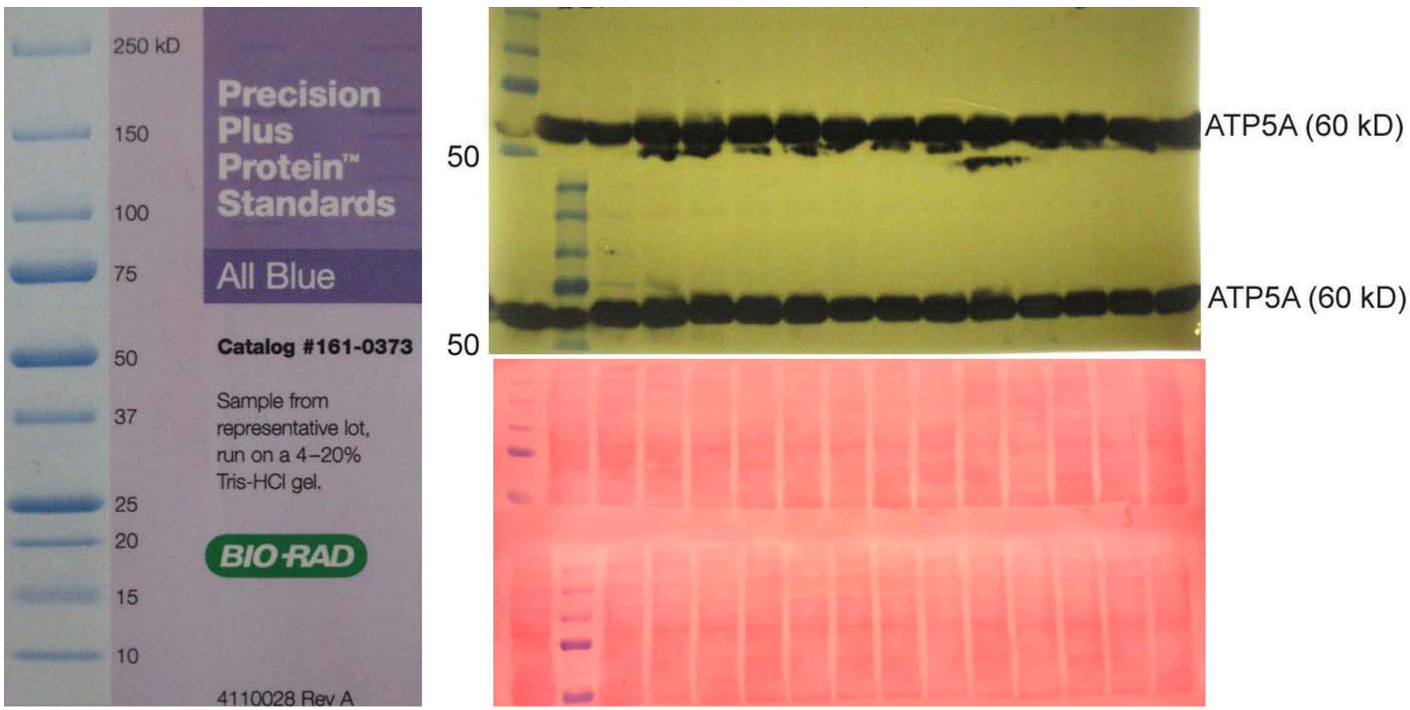

Supplementary Figure 27. Representative immunoblot image (Rg-film combined with nitrocellulose membrane) showing expression of ATP5A in the prefrontal cortex penumbra from rats with photochemically induced thrombosis (PT) (“Stroke + Saline” group), rats treated with Semax by i/n administration 2 h after PT and for the consecutive 6 days (1 instillation per day) at a dose 25 μg/kg (“Stroke + Semax” group), and intact rats treated with a 7-day course of Semax, for 21 days of observation. **Top sequence of blots** (first gel; one animal corresponds to each blot), from left to right: **1, 2** - “Control” (rats received saline); **3, 4** - “Stroke+Saline”, 24 h after PT; **5, 6** - “Stroke+Semax”, 24 h after PT; **7, 8** - intact rats receiving a single injection of Semax; **9, 10** - “Stroke+Saline”, 3 days after PT; **11, 12** - “Stroke+Semax”; 3 days after PT; **13, 14** - intact rats receiving a 3-days course of Semax; **Bottom sequence of blots** (second gel one; animal corresponds to each blot), from left to right: **1, 2** - “Control”; **3, 4** - “Stroke+Saline”, 7 days after PT; **5, 6** - “Stroke+Semax”, 7 days after PT; **7, 8** - intact rats receiving a 7-days course of Semax; **9, 10** - “Stroke+Saline”, 21 days after PT; **11, 12** - “Stroke+Semax”, 14 days after withdrawal of Semax treatment; **13, 14** - intact rats, 14 days after withdrawal of Semax treatment.

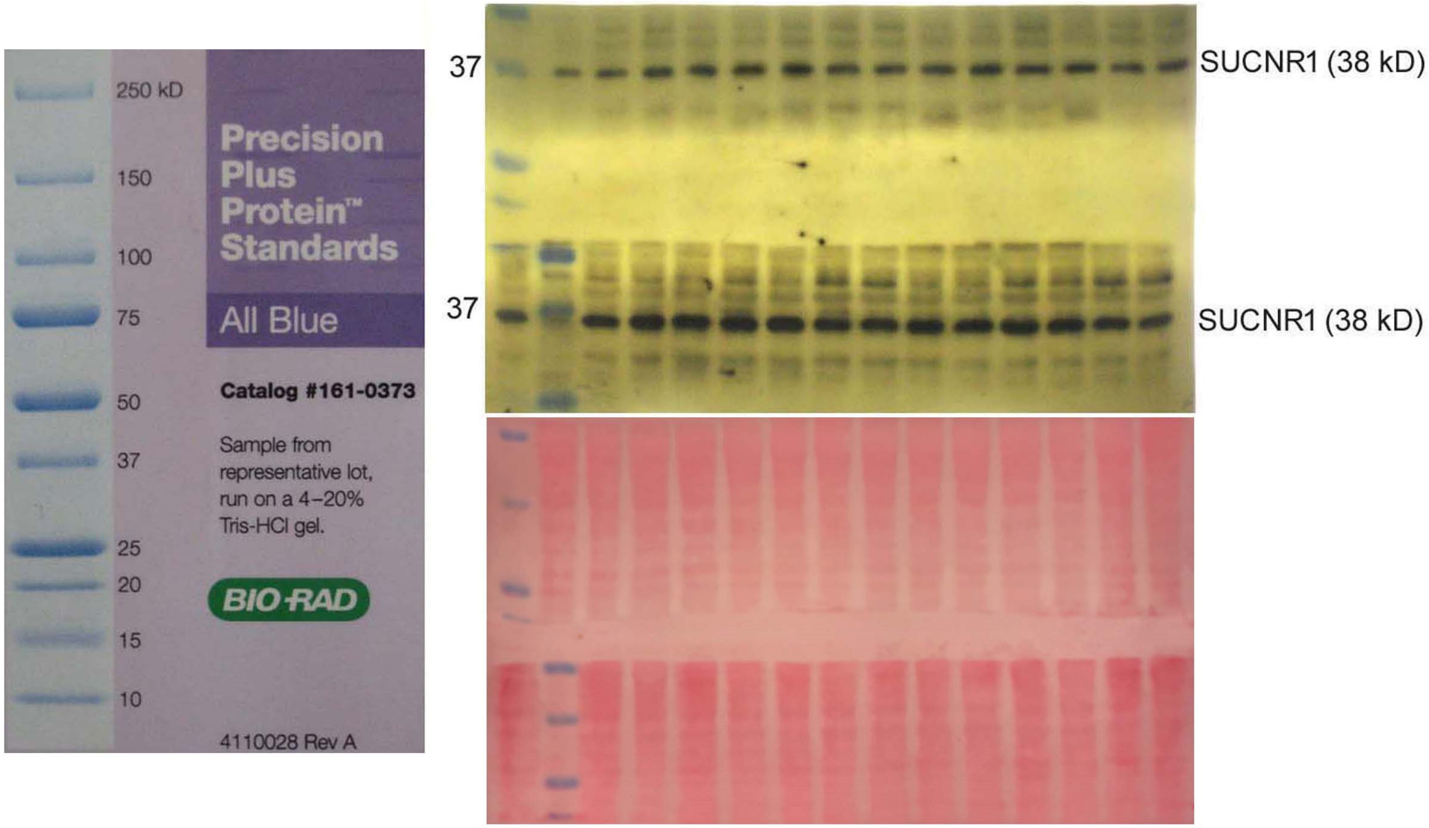

Supplementary Figure 28. Representative immunoblot image (Rg-film combined with nitrocellulose membrane) showing expression of SUCNR1 in the prefrontal cortex penumbra from rats with photochemically induced thrombosis (PT) ("Stroke + Saline" group), rats treated with Semax by i/n administration 2 h after PT and for the consecutive 6 days (1 instillation per day) at a dose 25 μg/kg ("Stroke + Semax" group), and intact rats treated with a 7-day course of Semax, for 21 days of observation. **Top sequence of blots** (first gel; one animal corresponds to each blot), from left to right: **1, 2** - "Control" (rats received saline); **3, 4** - "Stroke+Saline", 24 h after PT; **5, 6** - "Stroke+Semax", 24 h after PT; **7, 8** - intact rats receiving a single injection of Semax; **9, 10** - "Stroke+Saline", 3 days after PT; **11, 12** - "Stroke+Semax"; 3 days after PT; **13, 14** - intact rats receiving a 3-days course of Semax; **Bottom sequence of blots** (second gel one; animal corresponds to each blot), from left to right: **1, 2** - "Control"; **3, 4** - "Stroke+Saline", 7 days after PT; **5, 6** - "Stroke+Semax", 7 days after PT; **7, 8** - intact rats receiving a 7-days course of Semax; **9, 10** - "Stroke+Saline", 21 days after PT; **11, 12** - "Stroke+Semax", 14 days after withdrawal of Semax treatment; **13, 14** - intact rats, 14 days after withdrawal of Semax treatment.

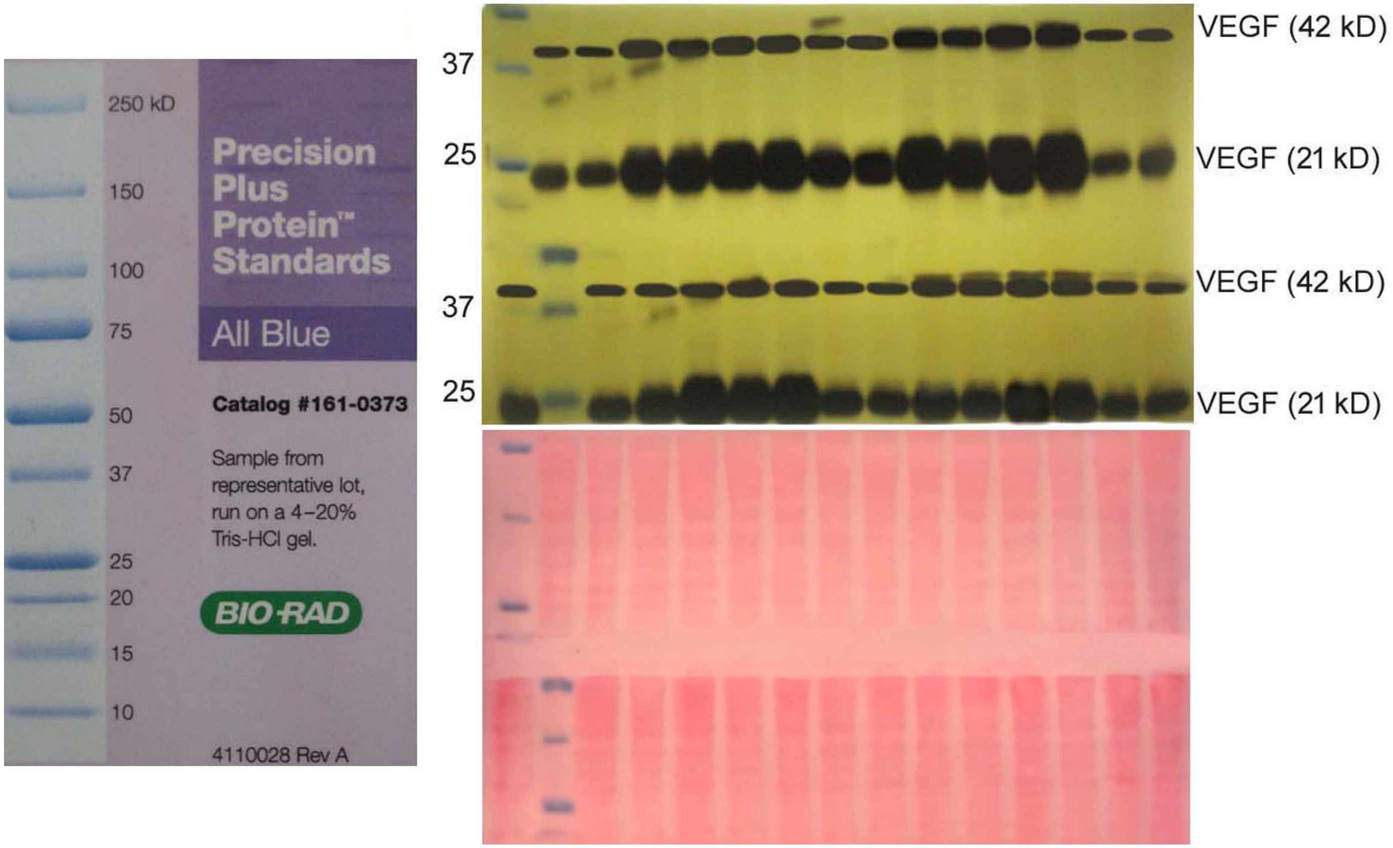

Supplementary Figure 29. Representative immunoblot image (Rg-film combined with nitrocellulose membrane) showing expression of VEGF in the prefrontal cortex penumbra from rats with photochemically induced thrombosis (PT) (“Stroke + Saline” group), rats treated with Semax by i/n administration 2 h after PT and for the consecutive 6 days (1 instillation per day) at a dose 25 µg/kg (“Stroke + Semax” group), and intact rats treated with a 7-day course of Semax, for 21 days of observation. **Top sequence of blots** (first gel; one animal corresponds to each blot), from left to right: **1, 2** - “Control” (rats received saline); **3, 4** - “Stroke+Saline”, 24 h after PT; **5, 6** - “Stroke+Semax”, 24 h after PT; **7, 8** - intact rats receiving a single injection of Semax; **9, 10** - “Stroke+Saline”, 3 days after PT; **11, 12** - “Stroke+Semax”; 3 days after PT; **13, 14** - intact rats receiving a 3-days course of Semax; **Bottom sequence of blots** (second gel one; animal corresponds to each blot), from left to right: **1, 2** - “Control”; **3, 4** - “Stroke+Saline”, 7 days after PT; **5, 6** - “Stroke+Semax”, 7 days after PT; **7, 8** - intact rats receiving a 7-days course of Semax; **9, 10** - “Stroke+Saline”, 21 days after PT; **11, 12** - “Stroke+Semax”, 14 days after withdrawal of Semax treatment; **13, 14** - intact rats, 14 days after withdrawal of Semax treatment.

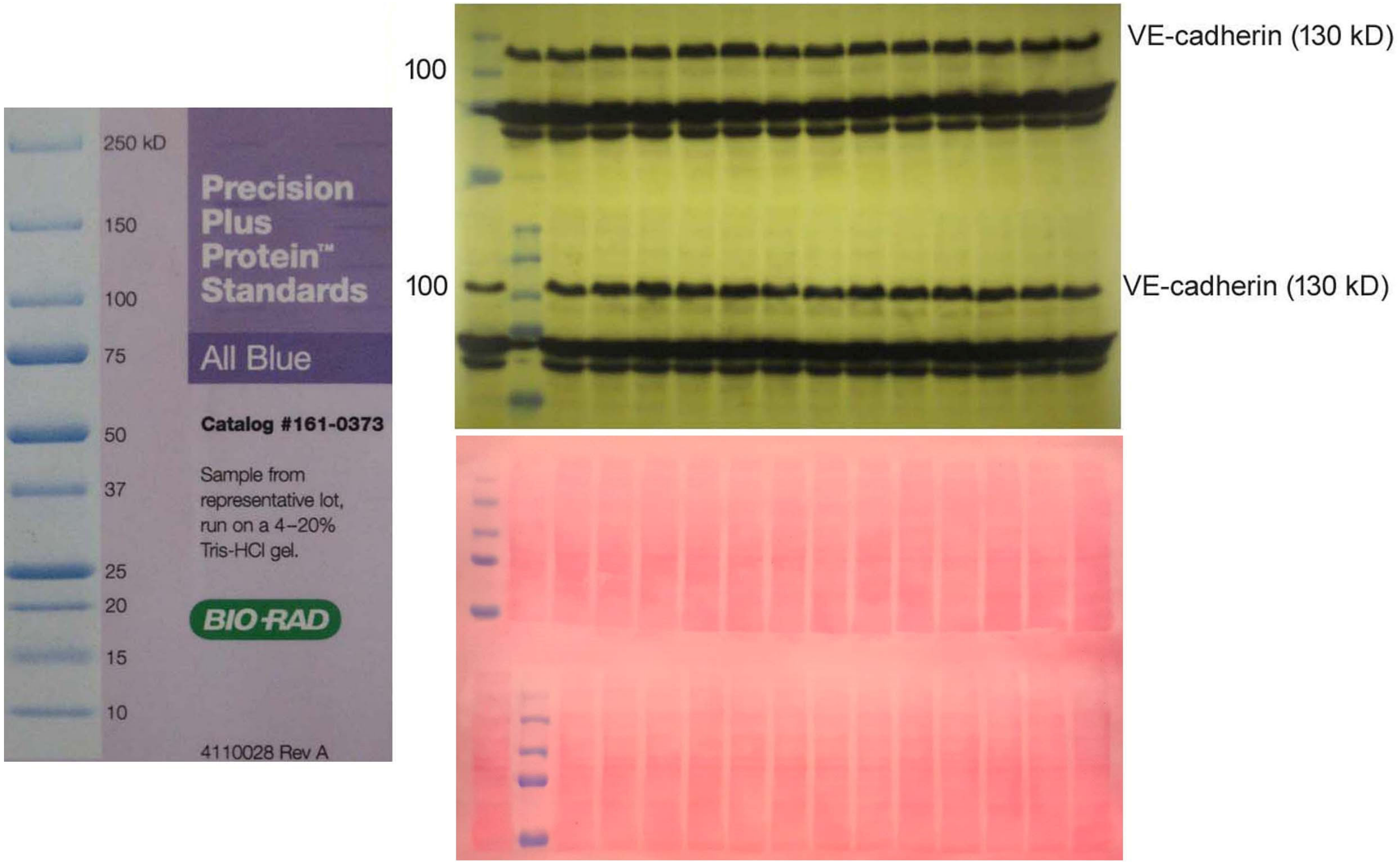

Supplementary Figure 30. Representative immunoblot image (Rg-film combined with nitrocellulose membrane) showing expression of VE-cadherin in the prefrontal cortex penumbra from rats with photochemically induced thrombosis (PT) ("Stroke + Saline" group), rats treated with Semax by i/n administration 2 h after PT and for the consecutive 6 days (1 instillation per day) at a dose 25 μg/kg ("Stroke + Semax" group), and intact rats treated with a 7-day course of Semax, for 21 days of observation. **Top sequence of blots** (first gel; one animal corresponds to each blot), from left to right: **1, 2** - "Control" (rats received saline); **3, 4** - "Stroke+Saline", 24 h after PT; **5, 6** - "Stroke+Semax", 24 h after PT; **7, 8** - intact rats receiving a single injection of Semax; **9, 10** - "Stroke+Saline", 3 days after PT; **11, 12** - "Stroke+Semax"; 3 days after PT; **13, 14** - intact rats receiving a 3-days course of Semax; **Bottom sequence of blots** (second gel one; animal corresponds to each blot), from left to right: **1, 2** - "Control"; **3, 4** - "Stroke+Saline", 7 days after PT; **5, 6** - "Stroke+Semax", 7 days after PT; **7, 8** - intact rats receiving a 7-days course of Semax; **9, 10** - "Stroke+Saline", 21 days after PT; **11, 12** - "Stroke+Semax", 14 days after withdrawal of Semax treatment; **13, 14** - intact rats, 14 days after withdrawal of Semax treatment.

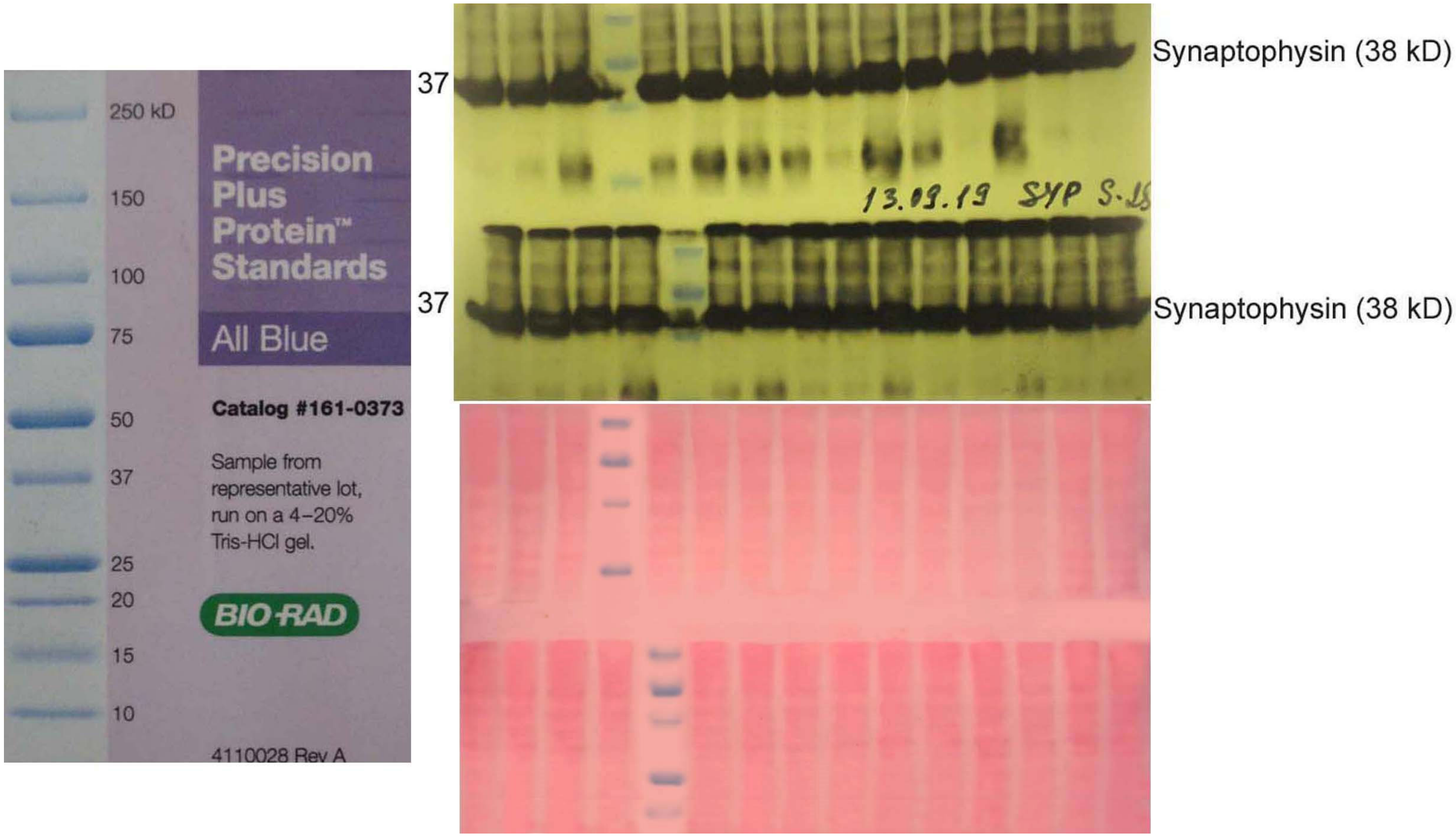

Supplementary Figure 31. Representative immunoblot image (Rg-film combined with nitrocellulose membrane) showing expression of Synaptophysin in the prefrontal cortex penumbra from rats with photochemically induced thrombosis (PT) (“Stroke + Saline” group), rats treated with Semax by i/n administration 2 h after PT and for the consecutive 6 days (1 instillation per day) at a dose 25 µg/kg (“Stroke + Semax” group), and intact rats treated with a 7-day course of Semax, for 21 days of observation. **Top sequence of blots** (first gel; one animal corresponds to each blot), from left to right: **1, 2** - “Control” (rats received saline); **3, 4** - “Stroke+Saline”, 24 h after PT; **5, 6** - “Stroke+Semax”, 24 h after PT; **7, 8** - intact rats receiving a single injection of Semax; **9, 10** - “Stroke+Saline”, 3 days after PT; **11, 12** - “Stroke+Semax”; 3 days after PT; **13, 14** - intact rats receiving a 3-days course of Semax; **Bottom sequence of blots** (second gel one; animal corresponds to each blot), from left to right: **1, 2** - “Control”; **3, 4** - “Stroke+Saline”, 7 days after PT; **5, 6** - “Stroke+Semax”, 7 days after PT; **7, 8** - intact rats receiving a 7-days course of Semax; **9, 10** - “Stroke+Saline”, 21 days after PT; **11, 12** - “Stroke+Semax”, 14 days after withdrawal of Semax treatment; **13, 14** - intact rats, 14 days after withdrawal of Semax treatment.
